# Animal degradation of microbial storage polyhydroxyalkanoates

**DOI:** 10.1038/s41559-026-03153-8

**Published:** 2026-08-13

**Authors:** Caroline Zeidler, Harald R. Gruber-Vodicka, Dolma Michellod, Grace D’Angelo, Stefan Becker, Henry Berndt, Juliane Wippler, Marlene Violette, Manuel Kleiner, Alba Porta-Fidalgo, Rebekka S. Janke, Kristina Weinert, Manuel Liebeke, Nicole Dubilier, Maggie Sogin

**Affiliations:** 1 Max Planck Institute for Marine Microbiology, Bremen, Germany; 2 Zoological Institute, University of Kiel, Kiel, Germany; 3 MARUM Center for Marine Environmental Sciences, University of Bremen, Bremen, Germany; 4 Department of Plant and Microbial Biology, North Carolina State University, Raleigh, NC USA; 5 Institute of Human Nutrition and Food Science, Department of Metabolomics, University of Kiel, Kiel, Germany; 6 Department of Molecular Cell Biology, University of California at Merced, Merced, CA USA

**Keywords:** Ecology, Microbiology

## Abstract

A wide range of microorganisms produce storage biopolymer polyhydroxyalkanoates (sPHAs) as carbon and energy reserves. However, only bacteria and fungi are known to degrade microbial sPHAs, using enzymes called polyhydroxyalkanoate depolymerases (PHADs). Here we show that some animals also have PHADs that can degrade sPHAs. We discovered a PHAD in the gutless oligochaete *Olavius algarvensis*, a marine worm that gains nutrition by digesting bacterial symbionts, including a dominant symbiont in which sPHAs account for up to 42% of cellular carbon stores. Enzyme assays, combined with mass spectrometry, confirmed that heterologously expressed *O. algarvensis* PHAD degraded sPHAs into hydroxyalkanoate monomers that can enter conserved metabolic pathways. Imaging of mRNA showed that PHAD was expressed in the oligochaete epidermis, the site of symbiont digestion. We further identified PHADs in more than 66 gut-bearing animal species from nine phyla and 19 protist species from three major supergroups, suggesting that the last common ancestor of metazoans possessed PHADs. Functional assays confirmed that PHADs from phylogenetically distant animal lineages spanning aquatic and terrestrial environments degrade sPHAs. These findings reveal a previously unrecognized pathway by which protists and animals can access microbial carbon reserves, with broad relevance given the widespread occurrence of sPHAs across ecosystems.

## Abstract (web summary)

This study shows that the degradation of microbial storage polyhydroxyalkanoates is not just restricted to microorganisms by finding that some animals also have the enzymes required to break down these biopolymers.

## Main

Storage polyhydroxyalkanoates (sPHAs) are natural polyesters produced by numerous bacteria and halophilic archaea that occur in a wide range of ecosystems, including soils, activated sludge and marine sediments^1–8^. Microorganisms synthesize these storage and energy compounds when carbon is available but other conditions for growth, such as oxygen or essential nutrients such as nitrogen and phosphate, are limiting. These sPHAs accumulate intracellularly as granules and can account for up to 90% of the dry weight of the microorganism^2,4,8–14^. When growth conditions become amenable again, microorganisms degrade sPHAs into their monomers, dimers or a mix of hydroxyalkanoate oligomers. These breakdown products are then degraded intracellularly to CO_2_, H_2_O and CH_4_, and in doing so produce reducing equivalents to fuel growth^15–22^. Considering how widespread sPHA-synthesizing microorganisms are in terrestrial and aquatic environments^1–4^, sPHAs probably contribute to carbon cycling by serving as a reservoir and a resource for carbon^16–21,23,24^.

Although many microorganisms accumulate high-molecular-weight sPHAs as carbon and energy reserves, animals and other eukaryotes produce only low-molecular-weight PHAs, commonly referred to as complexed PHAs (cPHAs) or oligoPHAs. These low-molecular-weight PHAs differ fundamentally in structure and function from sPHAs: they consist of relatively few hydroxyalkanoate units (typically 30–200) and are often covalently bound to proteins or associated with cellular membranes. Rather than serving as storage compounds, eukaryotic cPHAs are thought to play regulatory or structural roles within the cell^25–28^.

Until now, only bacteria, fungi and a few protists were thought to have the enzymatic machinery required to fully degrade sPHAs. The degradation of sPHA is catalysed by polyhydroxyalkanoate depolymerases (PHADs), carboxylesterases of the α/β-hydrolase superfamily which act either intracellularly or extracellularly (EC 3.1.1.75 and EC 3.1.1.76)^15,17–21,23,24^. Although widespread in bacteria and some archaea, the only eukaryotes assumed to have PHADs are fungi and two protist species^15,29–32^. In fungi, PHADs are widely distributed across major fungal groups, and experimental evidence confirms their ability to degrade sPHAs in at least 41 strains (mostly deuteromycetes; fungi imperfecti)^33^. Structural insight into eukaryotic PHADs is limited to a single fungal enzyme from *Penicillium funiculosum*, for which the crystal structure has been resolved^34^. Only sequence information is available for two protist species, *Acanthamoeba castellanii* and *Dictyostelium discoideum*, which have PHADs^31^. In animals, PHAD-like sequences have been annotated in metazoan genomes, but their origin and functional capacity to degrade sPHAs have not been experimentally shown.

Given the high carbon and energy contents of sPHAs, animals that feed on microorganisms directly or indirectly by ingesting soils, sediments or plankton would benefit from the ability to degrade sPHA. Here we show that many animals, as well as a far broader diversity of protists than previously recognized, encode PHADs in their genomes. We initially discovered an animal PHAD in the gutless marine oligochaete, *Olavius algarvensis*. These animals lack a mouth and gut, and depend entirely on their symbionts for their nutrition^35–38^. Imaging analyses and protein expression data indicate that, as in all other gutless oligochaetes, *O. algarvensis* acquires nutrients through phagolysosomal digestion of its symbionts within epidermal cells just below the symbiont-containing region^39–41^. The dominant symbiont in these hosts in terms of relative abundance and biomass is ‘*Candidatus* Thiosymbion algarvensis’ (hereafter *Ca.* T. algarvensis)^38^. This sulfur-oxidizing chemoautotrophic symbiont synthesizes large amounts of sPHA (0.7 µmol of carbon per worm), stored as a random copolymer of polyhydroxybutyrate (PHB), polyhydroxyvalerate (PHV) and polyhydroxymethylvalerate (PHMV), which together account for up to 42% of the carbon in the symbiont and 14% of the total carbon in the holobiont^42^.

After identifying a PHAD in *O. algarvensis*, we experimentally demonstrated its ability to degrade sPHAs, and revealed its expression using transcriptomics, proteomics and mRNA imaging analyses. We then expanded our analyses across eukaryotes, identifying PHAD homologues in other gutless oligochaetes, in animals with fully functional digestive systems from nine distantly related phyla and in protists from three major supergroups. Together, our results indicate that microbial sPHAs represent an important and previously underappreciated nutritional resource for a wide range of protists and animals.

## Results and discussion

### The gutless marine worm *O. algarvensis* encodes and expresses a PHAD

**Fig. 1:**
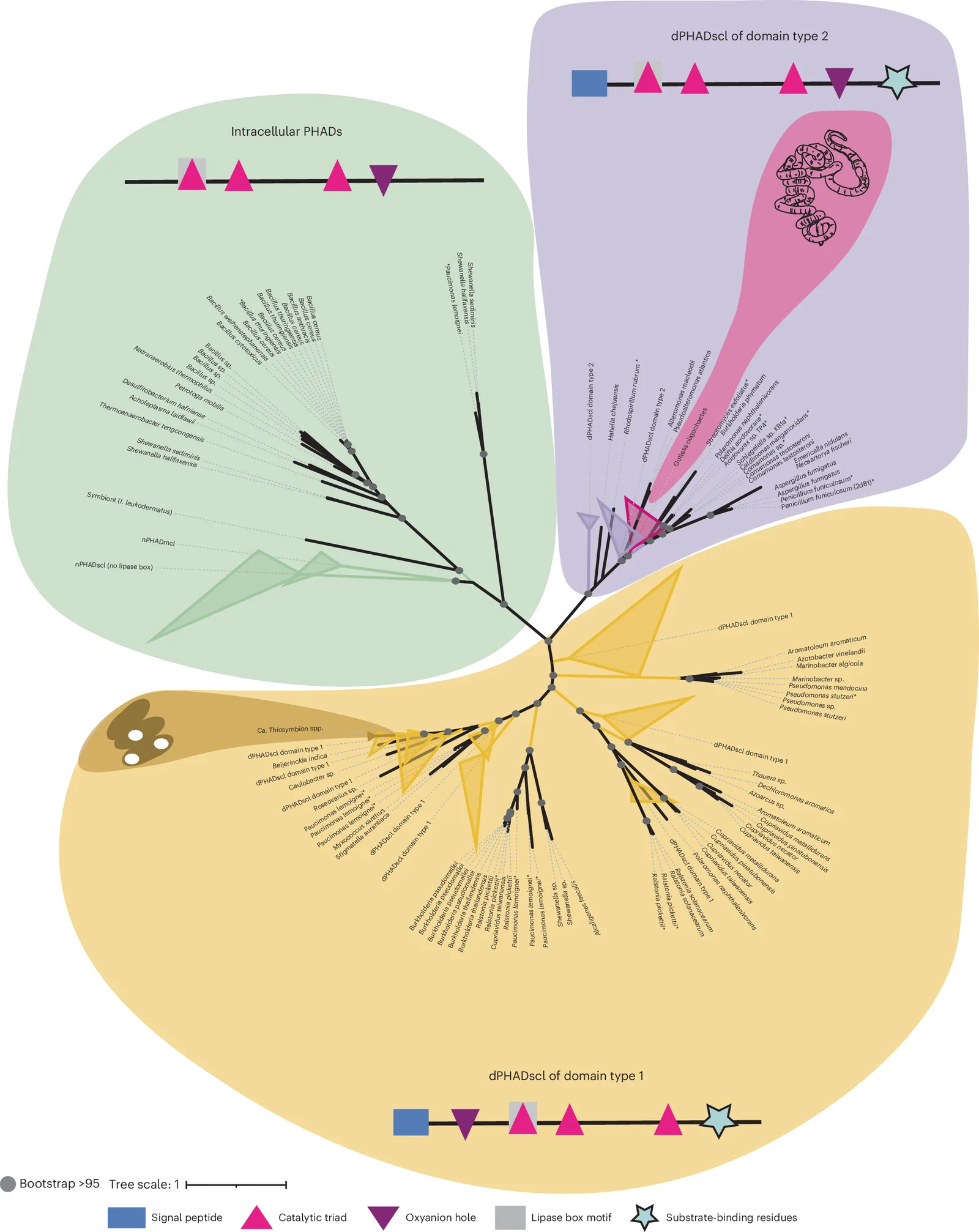
The PHADs of *O. algarvensis* and its symbiont *Ca.* T. algarvensis are phylogenetically distinct. The two enzymes fall into two different clades of extracellular PHADs (dPHADs). Comparative analyses of PHADs from gutless oligochaetes, their symbionts and the PHAD engineering database (DED) revealed three major clades: domain type 1 dPHADs (yellow), domain type 2 dPHADs (purple) and intracellular PHADs (green). Extracellular PHADs degrade denatured sPHAs outside the cell and the oxyanion hole (purple triangle) is positioned either before (domain type 1) or after (domain type 2) the catalytic triad (pink triangle). Intracellular PHADs degrade native sPHAs inside the cell, lack a signal peptide (blue box) and possess an undescribed substrate-binding site (cyan star). *O. algarvensis* PHADs group with domain type 2 dPHADs (pink clade), whereas those of their symbiont group with domain type 1 dPHADs (dark yellow clade). Experimentally validated PHAD sequences are marked with an asterisk (*). The tree was calculated using IQ-TREE with ultrafast bootstrap support (1,000 replicates; tree scale ranges from 0 to 1) and was based on an amino acid alignment.

The *O. algarvensis* PHAD gene, *phaZ*, spans a 19,000-base pair (bp) region in the genome of the animal and consists of ten exons separated by nine introns, confirming its eukaryotic origin^43^ (Supplementary Fig. 1). Phylogenetic analyses revealed that the PHAD of *O. algarvensis* is evolutionarily distinct from that of its symbiont *Ca.* T. algarvensis, with the two enzymes falling into distant clades representing different PHAD domain types (Fig. 1). Both enzymes group with extracellular PHADs predicted to degrade denatured, high-molecular-weight sPHAs composed of thousands of short-chain hydroxyalkanoate monomers (dPHADscl). The host enzyme most closely resembles dPHADscl of domain type 2, characterized by a lipase box preceding the oxyanion hole^15^, whereas the symbiont enzyme aligns with domain type 1, in which the order is reversed (Fig. 1). The clear phylogenetic separation of the animal and symbiont PHADs into different clades of PHAD domain types indicates that the host enzyme evolved independently of its symbiont’s counterpart.

To place both enzymes in the context of experimentally validated PHADs, we aligned host and symbiont PHADs to their closest biochemically characterized homologues in the PHAD Engineering Database (DED)^44^. The symbiont PHAD shared highest sequence similarity to domain type 1 dPHADscl from the bacterium *Ralstonia pickettii* (identity, 11.4%; coverage, 52.8%; similarity, 62%), while the host PHAD most closely matched domain type 2 dPHADscl from the fungus *P. funiculosum* (identity, 32.7%; coverage, 89.8%; similarity, 51%; Supplementary Table 1). These domain-type assignments are consistent with their respective phylogenetic placements (Fig. 1).

**Fig. 2:**
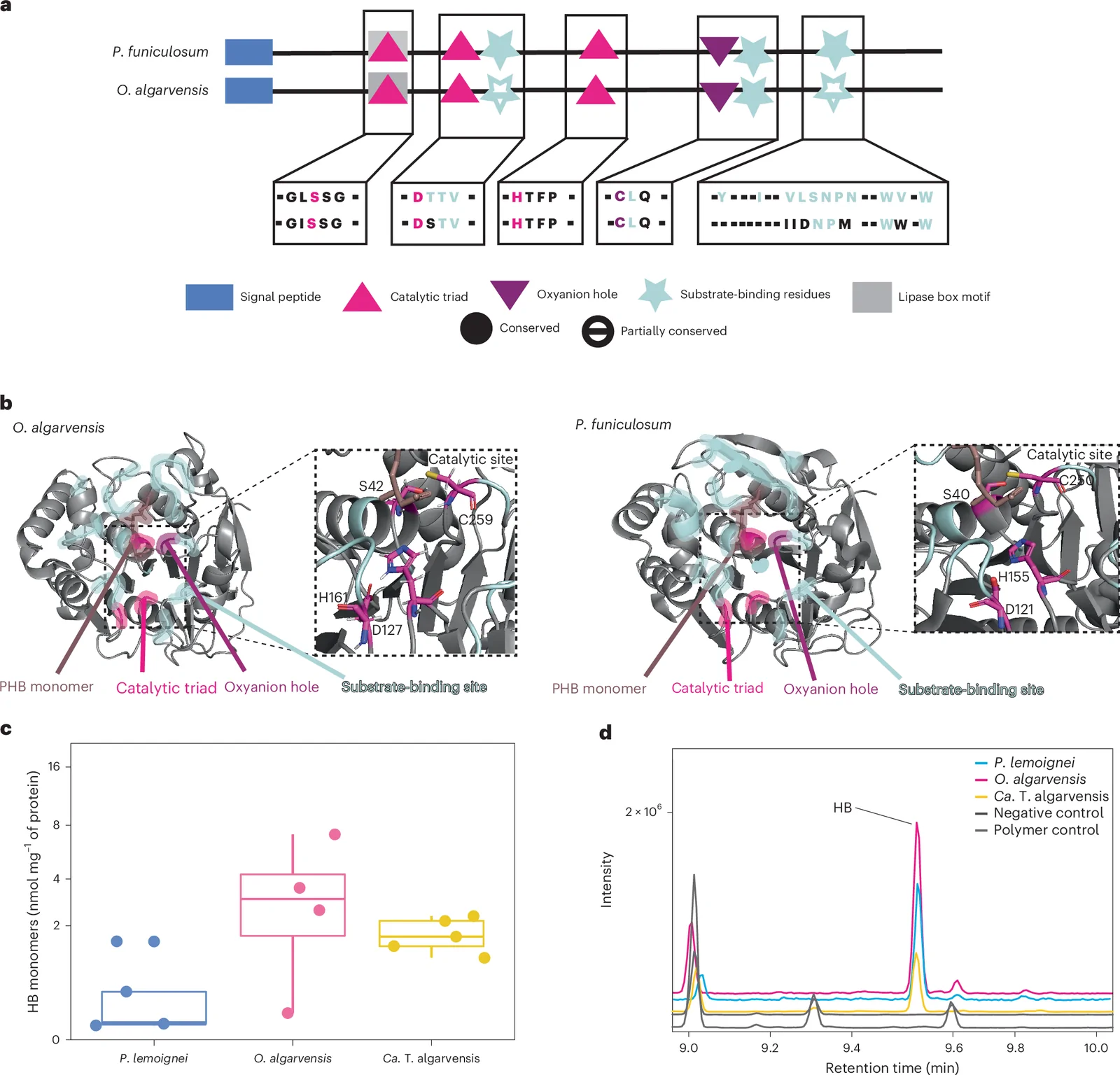
*O. algarvensis* PHAD degrades PHB into hydroxybutyrate (HB) monomers. **a**, A comparison between the primary structures of the *phaZ* gene between *O. algarvensis* and the fungus *P. funiculosum*. The *O. algarvensis* protein spans 333 amino acids; the *P. funiculosum* PHAD has 318 amino acids. The signal peptide spanning the first 20 amino acids (blue square), the catalytic triad including the catalytic serine residue (S42, D127, H167; pink triangles) located in the lipase box (grey box) and the C259 residue of oxyanion hole (purple triangle) are fully conserved between the two enzymes (filled symbols). The substrate-binding domain is only partially conserved (partially filled light-blue star) and shares 29.2% identity to that of the fungal homologue. All other residues in the primary structure are represented by the black line. **b**, The AlphaFold2-predicted tertiary structure of the *O. algarvensis* PHAD (left) and the crystal structure of the homologue from *P. funiculosum* (right) show strong similarity based on the superposition of the two enzymes using PyMol (PDB 2d81; r.m.s.d. 0.773 Å; r.m.s.d. catalytic domain 0.045–0.822 Å; r.m.s.d. substrate-binding domain 0.471 Å). The insets show the catalytic triad (dark pink) that surrounds the PHB (brown) based on the prediction of the model (oxyanion hole in purple; substrate-binding domain in light cyan). **c**,**d**, GC–MS analyses of PHAD assays using PHB. **c**, Boxplots show the concentration of HB monomers resulting from enzymatic degradation of PHB. The *O. algarvensis* PHAD (pink; *n* = 4 biological replicates from one heterologous expression, upper whisker = 7.1 nmol mg^−1^ of protein; lower whisker = 0.2 nmol mg^−1^ of protein; Q1 = 1.4 nmol mg^−1^ of protein; Q3 = 5.3 nmol mg^−1^ of protein; median = 3.1 nmol mg^−1^ protein) produces more HB monomers than its symbiont (*Ca.* T. algarvensis, yellow; *n* = 5 biological replicates from one heterologous expression, upper whisker = 2.3 nmol mg^−1^ of protein; lower whisker = 1.1 nmol mg^−1^ of protein; Q1 = 1.4 nmol mg^−1^ of protein; Q3 = 2.2 nmol mg^−1^ of protein; median = 1.7 nmol mg^−1^ of protein) and the bacterial PHAD used as a positive control (*P. lemoignei*, blue; *n* = 5 biological replicates from one heterologous expression, upper whisker = 0.5 nmol mg^−1^ of protein, lower whisker = 0.01 nmol mg^−1^ of protein; Q1 = 0.03 nmol mg^−1^ of protein; Q3 = 0.5 nmol mg^−1^ of protein; median = 0.03 nmol mg^−1^ of protein). **d**, Total ion chromatograms show the relative peak heights of the HB monomers in representative assays (9.56 min). No HB was detected in assays using a heat-inactivated PHAD (dark grey) or controls with only the polymers; light grey, Supplementary Fig. 3).

We next compared the AlphaFold2-predicted structure of the *O. algarvensis* PHAD with the experimentally validated homologue exhibiting the highest sequence identity within its domain. The only structurally characterized eukaryotic enzyme within the domain type 2 clade is the PHAD from the fungus *P. funiculosum* (PDB 2d81)^34^, which shares 31.2% amino acid identity with the *O. algarvensis* PHAD (see Supplementary Texts 1 for the symbiont PHAD predicted structure and 2 for the host PHAD predicted structure). Structural comparison between the two enzymes revealed that the catalytic triad and oxyanion hole responsible for the nucleophilic attack on PHA are conserved and spatially aligned (Fig. 2 and Supplementary Text 2). A predicted signal peptide at the N terminus of the *O. algarvensis* PHAD suggests translocation to secretory pathways, consistent with secretion into the phagolysosomes where symbionts are digested and their sPHAs become accessible (Supplementary Table 2).

To assess whether the *O. algarvensis* PHAD is expressed, we analysed total and host-enriched mRNA libraries and proteomic samples. The PHAD was expressed in 12 out of 15 individuals (Supplementary Table 3), indicating that the enzyme is active in vivo. The three libraries without detectable transcripts originated from total RNA datasets, in which host transcripts are often under-represented because of the high abundance of bacterial and ribosomal RNA reads. We also detected the animal PHAD in the proteomes of *O. algarvensis* (*n* = 8; Supplementary Texts 3 and 8 and Supplementary Table 4).

We next tested whether the *O. algarvensis* PHAD can degrade denatured sPHAs. We chose denatured sPHA for our assays for the following reasons: within intact symbiont cells, sPHAs are probably amorphous rather than denatured, as observed in other bacteria^45^. However, the host cannot access or degrade sPHA within intact symbionts as this would require translocation of its PHAD across the bacterial envelope, a process not known for any animal enzyme. Consequently, PHA degradation can only occur once the host initiates phagolysosomal digestion of its symbionts. During this process, sPHAs are released from the symbiont cells and become denatured, as shown for other sPHA-containing bacteria that are lysed^15^. Such denatured sPHAs represent the canonical substrate for extracellular PHADs^15^, consistent with the phylogenetic placement of the gutless oligochaete PHADs within the dPHADscl clade, which comprises PHADs known to degrade denatured sPHAs (Fig. 1).

To experimentally test whether the *O. algarvensis* PHAD can degrade denatured sPHAs, we heterologously expressed it in *Escherichia coli*. Spot assays with the enzyme on plates containing denatured sPHA, as either PHB or a mixture of PHB with 3% PHV, showed clearance zones after 24 h. Gas chromatography–mass spectrometry (GC–MS) analyses of extracts from these zones confirmed that the *O. algarvensis* PHAD degraded both PHB and PHB/PHV into hydroxybutyrate (HB) monomers (Fig. 2 and Supplementary Figs. 2–4). The *O. algarvensis* enzyme degraded sPHAs at similar efficiencies to those of the well-characterized, extracellular PHAD from the bacterium *Paucimonas lemoignei*^46^, degrading 11% of the polymer compared with 14% by *P. lemoignei* (Fig. 2 and Supplementary Figs. 2–4; for calculations see Supplementary Table 5). Together, these results demonstrate that the animal PHAD is a functional homologue of microbial PHADs capable of degrading denatured sPHAs.

**Fig. 3:**
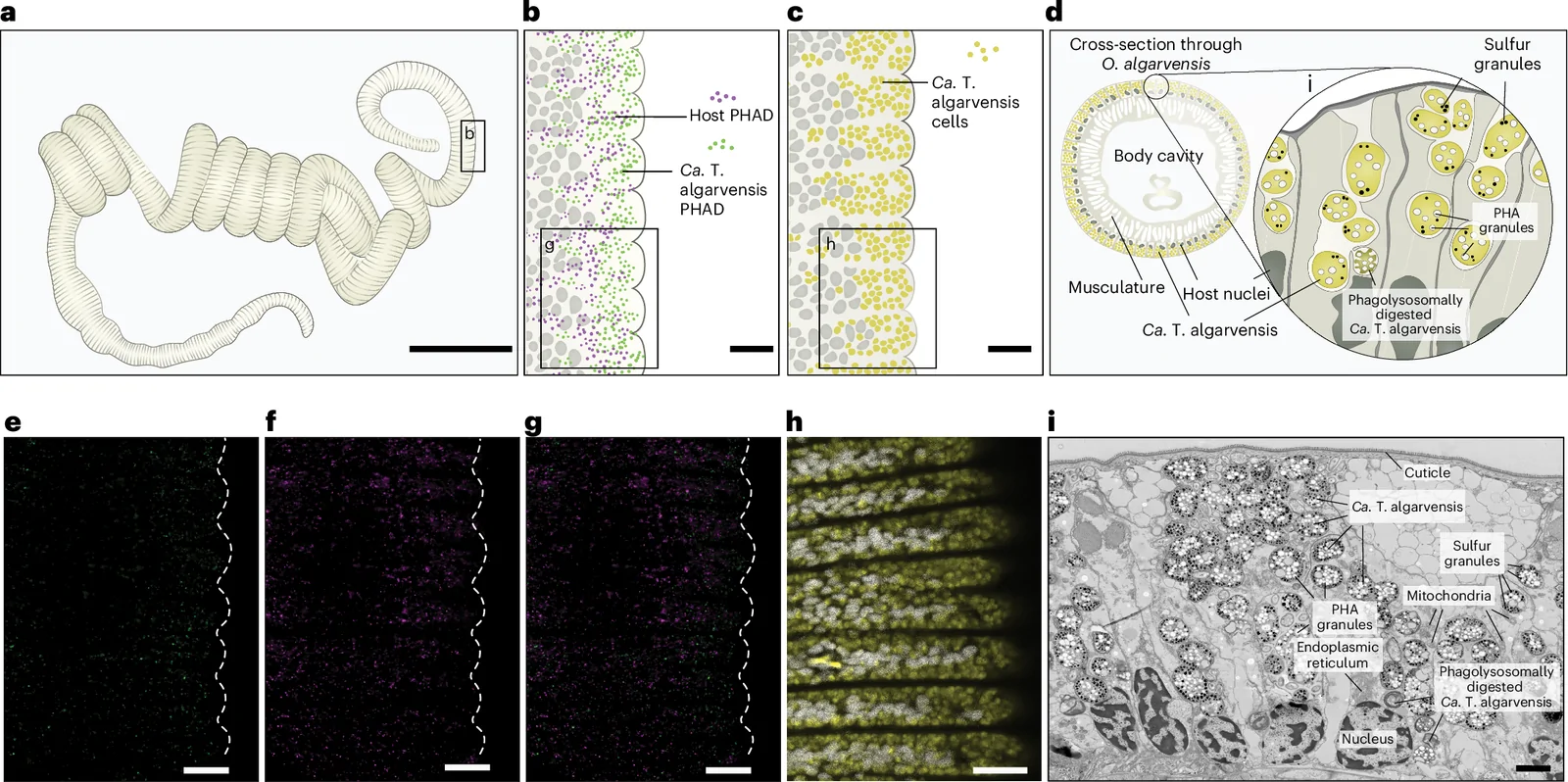
The PHAD of *O. algarvensis* is expressed in its epidermal cells where symbionts are digested through phagolysosomal activity. **a**, Schematic drawing of *O. algarvensis* highlighting the focal planes shown in **b**–**d**. **b**, Schematic overview of HCR-FISH showing PHAD transcripts from the symbiont *Ca.* T. algarvensis (green; **e**) and its host (pink; **f**). Symbiont and host signals do not overlap, as shown in the merged image (**g**). **c**, The symbionts (yellow), visualized by whole-mount 16S rRNA FISH (**h**), are located directly beneath the cuticle of the host (gammaproteobacterial probe GAM42a; host nuclei stained with DAPI in grey). **d**, Schematic of host epithelial cells showing symbionts and their phagolysosomal digestion based on the transmission electron microscopy image in **i** (representative image based on one worm). The symbionts contain two types of granules: large sulfur vesicles (light grey; S^0^ 212.7 nmol per average worm) and smaller, more numerous sPHA granules (black to white; 0.7 µmol of carbon per worm). **e**, *Ca.* T. algarvensis PHAD. **f**, Host PHAD. **g**, Merged host and symbiont PHAD. **h**, *Ca.* T. algarvensis 16S rRNA and host nuclei. **i**, *O. algarvensis* epithelial cells TEM. Scale bars: 6 mm (**a**), 20 µm (**b**,**c**,**e**–**h**), 2 µm (**i**). See Supplementary Figs. 5–7 for imaging replication (HCR-FISH *n* = 6 worms), controls and focal plane analyses.

To determine where the *O. algarvensis* PHAD is expressed, we performed whole-mount hybridization chain reaction-fluorescence in situ hybridization (HCR-FISH) using probes specific to the mRNA of both the host and symbiont PHADs. The *O. algarvensis* probe hybridized to transcripts in the epidermal cells directly beneath the cuticle of the worm, the site of phagolysosomal digestion of symbionts (Fig. 3e–g). In contrast, transcripts of the symbiont PHAD localized to the bacterial symbionts in the overlying symbiont layer (Fig. 3e,g,h). The absence of signal overlap confirms that the host and symbiont PHADs represent distinct transcripts expressed in different regions of the body wall of the worm. Because phagolysosomal digestion of symbionts occurs within the host epidermal cells (Fig. 3d,i)^39,47^, these results indicate that *O. algarvensis* expresses its PHAD specifically at the site of symbiont digestion.

### Genes for degrading sPHAs are common to gutless oligochaetes

Given that all gutless oligochaete species investigated to date have sPHA-synthesizing symbionts^48^, we hypothesized that other gutless oligochaetes also encode and express PHADs. We screened total and host-enriched mRNA libraries from 14 additional gutless oligochaete species representing the phylogenetic diversity of these hosts. PHAD transcripts were recovered from all species, with one to six homologues per species showing 25% to 80% amino acid identity to the *O. algarvensis* PHAD (Supplementary Fig. 8a, Supplementary Table 3 and Supplementary Data 1). All host PHAD sequences form a monophyletic clade and are phylogenetically distinct from their corresponding symbiont PHADs, confirming their independent evolutionary origins (Fig. 1).

As in *O. algarvensis*, the PHADs from other gutless oligochaetes contain a predicted signal peptide (eight out of nine species) and their catalytic and substrate-binding sites align with the crystal structure of *P. funiculosum* (Supplementary Text 2, Supplementary Fig. 8b and Supplementary Table 2). All ten gutless oligochaete species encode PHAD homologues, ranging from one to six per genome. In bacteria, several PHAD isoforms confer the ability to degrade different types of sPHA, such as PHB, PHV, PHMV, making them metabolically flexible^49–53^ (Supplementary Text 4). By analogy, gutless oligochaetes with several PHAD variants may use them to degrade diverse sPHA polymers synthesized by their symbionts^42^.

**Fig. 4:**
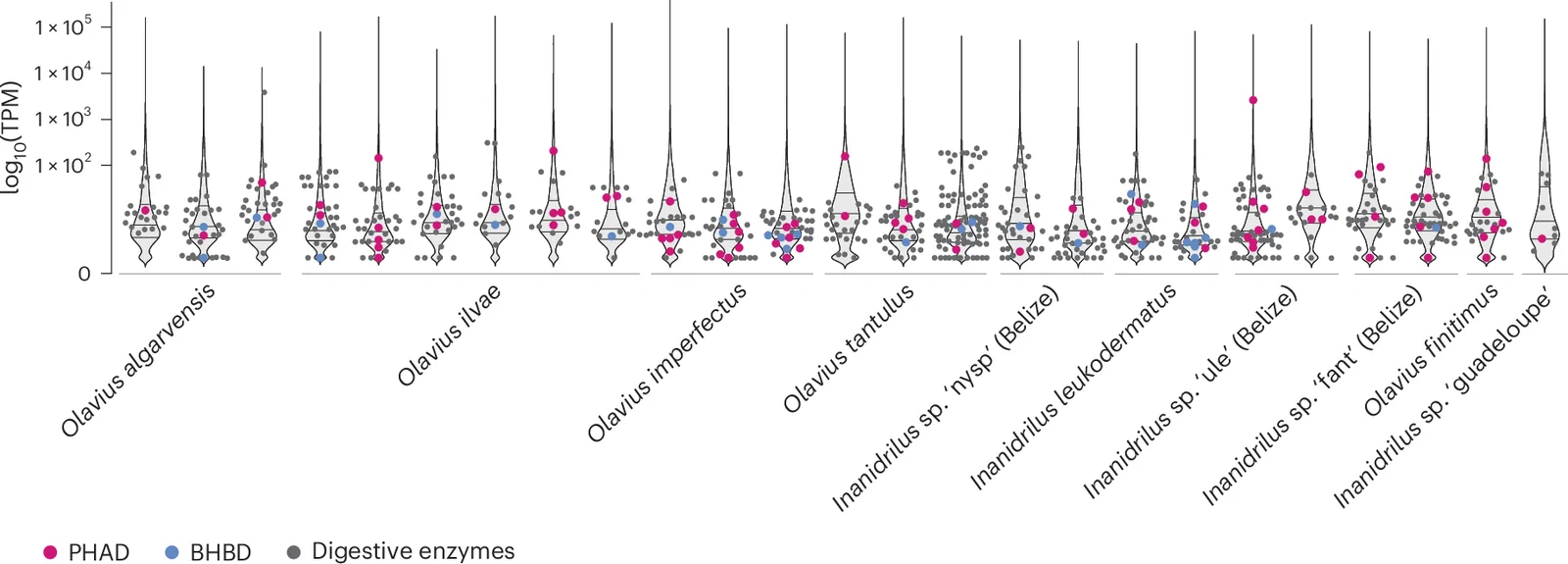
Gutless oligochaetes express enzymes required for sPHA degradation. We quantified PHAD and BHBD transcript abundances in the ten species of the genus *Olavius* and *Inanidrilus* for which we had host-enriched transcriptomes (the transcriptomes from the other five gutless oligochaete species with PHADs were from total RNA (host and symbiont) and could therefore not be used to quantify host transcripts). Violin plots show assembled host transcriptomes of single individuals from ten species (shown as normalized transcription (log_10_(TPM)) per individual worm), with PHAD transcripts shown in pink and BHBD transcripts in blue. PHAD and BHBD transcript abundances are similar to those of other digestive enzymes (grey points), with BHBD detected in eight and PHAD in all ten host species examined. To estimate transcript abundances, we assembled one reference assembly per species and mapped the raw reads of the libraries from single individuals to the reference assembly, to obtain normalized transcript abundances. TPM, transcripts per kilobase million values.

Finally, we compared the expression levels of PHADs and host enzymes involved in phagolysosomal digestion across ten species from diverse habitats (Fig. 4). PHADs and digestive enzymes showed comparable expression levels across all individuals and species in host-enriched transcriptomes, supporting the conclusion that symbiont-derived sPHAs represent a consistent and valuable source of nutrition for gutless oligochaetes. We also detected the expression of the β-hydroxybutyrate dehydrogenase (BHBD) gene needed for downstream sPHA catabolism in eight gutless oligochaete species (Fig. 4; more details for BHBD in the following section).

### Symbionts may not be able to metabolize the sPHAs they produce

Having shown that gutless oligochaetes express active PHADs that degrade symbiont-derived sPHAs, we next asked whether their *Thiosymbion* symbionts have the genes required for metabolizing the sPHAs they synthesize. All 15 *Thiosymbion* species encode a PHAD that clusters phylogenetically with those annotated as extracellular enzymes (Fig. 1 and Supplementary Data 1). Such extracellular PHADs are found in microorganisms that degrade sPHAs in their environment, while all microorganisms that synthesize sPHAs rely on intracellular PHADs to mobilize their storage compounds^15,54^.

We therefore interpret the apparent extracellular placement of the *Thiosymbion* PHADs as a case of phylogenetic misannotation based on sequence similarity rather than experimentally validated cellular localization. Consistent with intracellular PHADs that have been experimentally validated^15,44^, the *Thiosymbion* PHAD lacks a signal peptide and has a yet-unidentified substrate-binding domain. Importantly, the capacity of *Thiosymbion* to synthesize sPHAs provides strong functional evidence that their PHADs are intracellular, as all known sPHA-producing microorganisms require such an enzyme to control polymer accumulation^54–56^. Although our heterologous enzyme assays showed that the *Ca.* T. algarvensis PHAD degraded denatured sPHA in vitro, this has also been shown for other intracellular PHADs^57,58^ and is therefore consistent with, rather than indicative of, an extracellular function^15^.

The retention of an active PHAD in *Thiosymbion* raises the question whether the symbionts can further metabolize the products of sPHA depolymerization. To address this, we searched symbiont genomes and transcriptomes for enzymes required to process sPHA-derived monomers and oligomers. Across all 15 host species examined, we found no evidence for the three enzymes known to catalyse downstream oxidation of hydroxyalkanoate intermediates: BHBD (EC 1.1.1.30), hydroxybutyrate-dimer hydrolase (EC 3.1.1.22) and 3-hydroxybutyryl-CoA dehydrogenase (EC 1.1.1.157) (Supplementary Table 6 and Supplementary Text 5). At least one of these enzymes is required to oxidize sPHA-derived intermediates and generate reducing equivalents for energy metabolism, indicating that *Thiosymbion* lacks the capacity to use sPHAs as an energy source. While non-specific hydrolases could contribute to limited processing of primary PHAD products, the absence of the canonical catabolic pathway suggests that efficient use is unlikely.

The uniform absence of downstream sPHA catabolic genes in *Thiosymbion* from all 15 host species examined, spanning the phylogenetic diversity of gutless oligochaetes^48^, suggests that this gene loss occurred early in the evolution of the symbiosis and conferred a strong selective advantage. In contrast to their symbionts, eight host species for which we had host-enriched transcriptomes expressed a BHBD (Fig. 4), enabling them to metabolize sPHA-derived intermediates and capture the released carbon during phagolysosomal digestion (Supplementary Text 5). Together, these findings indicate that in gutless oligochaetes, selection favours symbionts that retain sPHA synthesis and intracellular PHAD activity, while lacking the downstream enzymatic machinery required to catabolize sPHA-derived carbon. This metabolic configuration channels the energetic value of sPHAs to the host while maintaining a healthy and productive symbiont population before digestion.

An additional non-energetic benefit of retaining symbiont PHADs is that partial sPHA depolymerization may release methyl-branched hydroxyalkanoate products that could protect metabolically active symbionts against oxidative stress^59^. Such a role would enhance symbiont stability and productivity, while conserving sPHA-derived carbon for the host. The retention of the intracellular PHAD from *Thiosymbion* could thus simultaneously support symbiont stress resistance and maximize host access to energy- and carbon-rich sPHAs. In summary, retention of PHAD in *Thiosymbion* appears to be selectively maintained: necessary to regulate sPHA content within symbiont cells and potentially to support symbiont stress resilience, while the loss of downstream pathways in the symbionts ensures exclusive host access to sPHA-derived carbon. This partitioning of metabolic functions provides a coherent evolutionary explanation for the observed gene complement and highlights how host control can shape symbiont metabolism to maximize host nutrient acquisition.

### Animals from nine phyla spanning major marine and terrestrial animal lineages encode PHADs

We finally asked if other animals besides gutless oligochaetes have PHADs, as these enzymes would provide a nutritional advantage to metazoans that feed on soil, sediments or other substrates rich in microbial sPHA. Our searches for homologues of the gutless oligochaete PHADs in public databases revealed 195 PHADs across 66 animal species spanning 9 metazoan phyla (Supplementary Table 7). We also expanded the known diversity of protist PHADs from 2 to 18 species across 3 supergroups and identified 48 homologues in total (for more details on the protist PHADs see Supplementary Text 6 and Supplementary Tables 8 and 9).

To assess whether these sequences represent bona fide PHADs rather than unrelated hydrolases, we aligned all predicted animal and protist enzymes to the only structurally characterized eukaryotic PHAD, from the fungus *P. funiculosum* (PDB 2d81)^34^. The newly identified PHADs exhibit complete conservation of the catalytic triad and oxyanion hole (100% coverage, 60–100% identity) and partial conservation of the substrate-binding site (52–93% coverage, 8–42% identity). Moreover, ~75% of the predicted animal PHADs contain a signal peptide, indicating that these enzymes are probably secreted (Supplementary Fig. 9 and Supplementary Tables 2 and 7).

We next tested whether the recovered animal sequences might belong to related α/β-hydrolase families such as cutinases or lipases, which share similar catalytic motifs but cannot completely hydrolyse sPHA^60,61^. To distinguish true PHADs from these enzymes, we performed phylogenetic analyses that included bacterial, fungal, protist and animal PHADs, cutinases and lipases. The resulting trees show that PHADs form a distinct clade separate from the other two enzyme classes (Supplementary Figs. 10 and 11). These phylogenetic differences are mirrored in the primary structure, where the catalytic triad and substrate-binding motifs of the PHADs do not align with those of cutinases or lipases (Supplementary Figs. 10 and 11).

**Fig. 5:**
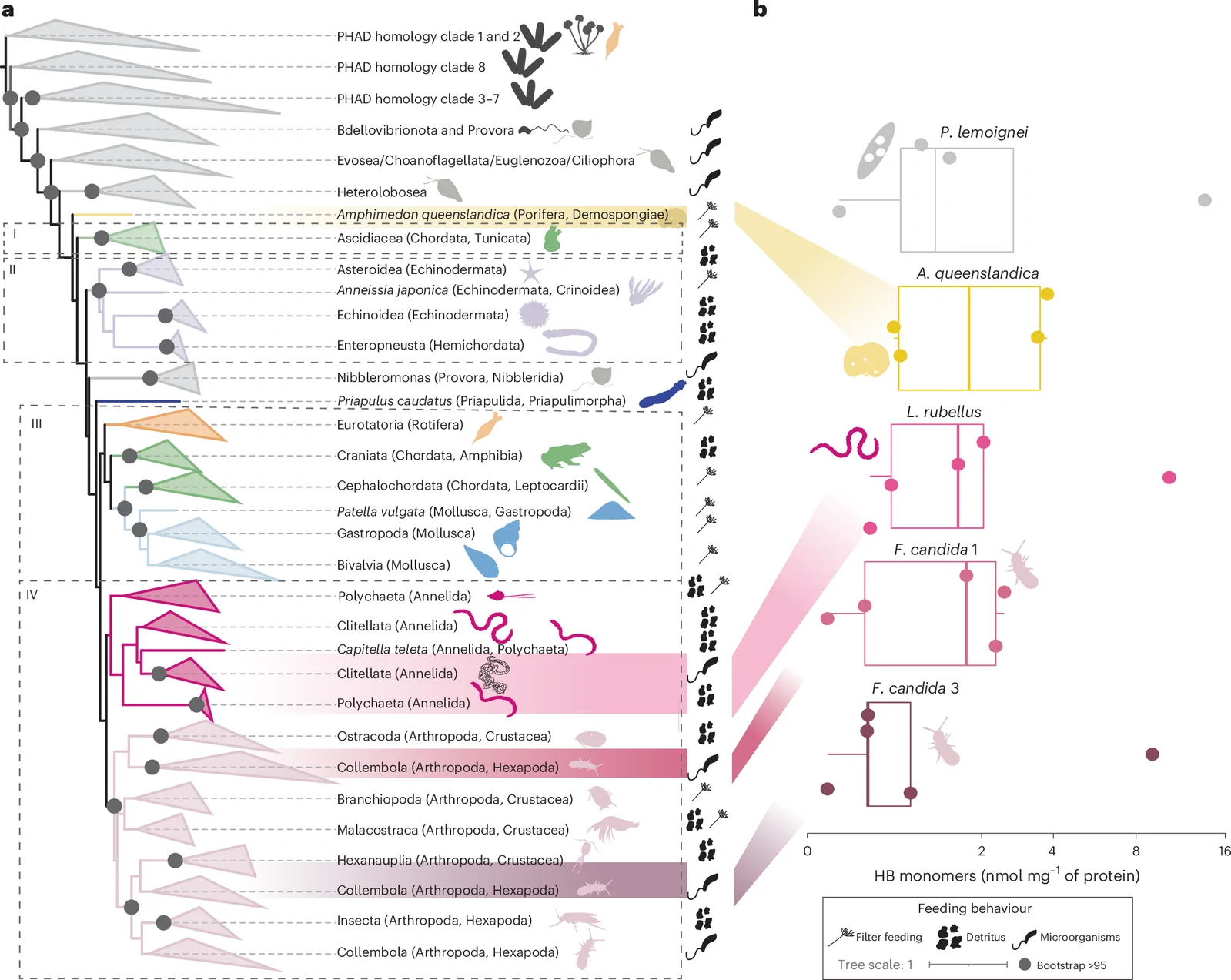
PHADs are widespread across animals. **a**, Maximum-likelihood tree of 208 PHAD sequences from animals spanning nine phyla, calculated using IQ-TREE with ultrafast bootstrap support (1,000 replicates; tree scale ranges from 0 to 1). The tree includes representatives from Porifera (yellow), Chordata (green), Hemichordata and Echinodermata (purple), Priapulida (dark blue), Rotifera (orange), Mollusca (blue), Annelida (pink) and Arthropoda (light pink); gutless oligochaete PHADs are highlighted in bright pink, forming four subclades (I to IV). Reconstruction of the feeding ecology of PHAD-bearing animals and protists indicates that these organisms may acquire sPHAs by consuming sPHA-producing microorganisms directly or indirectly through soil, sediment or detritus. Icons denote feeding behaviour and colour ranges correspond to the phylum of origin. Coloured bars mark sequences that showed PHAD activity in our assays. **b**, Boxplots show the concentrations of HB monomers, measured by GC–MS, produced in PHAD enzyme assays using PHB. All tested enzymes, including those from the sponge *A. queenslandica* (yellow; *n* = 4 biological replicates of one heterologous expression; upper whisker = 3.8 nmol mg^−1^ of protein, lower whisker = 0.6 nmol mg^−1^ of protein; Q1 = 0.6 nmol mg^−1^ of protein; Q3 = 3.6 nmol mg^−1^ of protein; median = 2.1 nmol mg^−1^ of protein), the springtail *F. candida* (light pink, two homologues; *n* = 5 biological replicates of one heterologous expression*; F. candida* 1: upper whisker = 2.5 nmol mg^−1^ of protein, lower whisker = 5.6 × 10^15^ nmol mg^−1^ of protein; Q1 = 0.32 nmol mg^−1^ of protein; Q3 = 2.3 nmol mg^−1^ of protein; median = 1.7 nmol mg^−1^ of protein, *F. candida* 3: upper whisker = 0.8 nmol mg^−1^ of protein, lower whisker = 6.1 × 10^−15^ nmol mg^−1^ of protein; Q1 = 0.3 nmol mg^−1^ of protein; Q3 = 0.8 nmol mg^−1^ of protein; median = 0.3 nmol mg^−1^ of protein) and the earthworm *L. rubellus* (pink; *n* = 5 biological replicates of one heterologous expression; upper whisker = 10.4 nmol mg^−1^ of protein, lower whisker = 0.4 nmol mg^−1^ of protein; Q1 = 0.9 nmol mg^−1^ of protein; Q3 = 6.2 nmol mg^−1^ of protein; median = 1.8 nmol mg^−1^ of protein), generated measurable HB monomers. No HB monomers were detected in the non-sense vector negative control or polymer blank samples (*n* = 5 biological replicates of one heterologous expression; Supplementary Figs. 12 and 13; assays done on PHB/PHV, Supplementary Fig. 14). As a positive control, we used the PHAD of the bacterium *P. lemoignei* (grey; *n* = 4 biological replicates of one heterologous expression; upper whisker = 13.7 nmol mg^−1^ of protein, lower whisker = 0.1 nmol mg^−1^ of protein; Q1 = 0.5 nmol mg^−1^ of protein; Q3 = 7.5 nmol mg^−1^ of protein; median = 1.2 nmol mg^−1^ of protein). Credit: Silhouettes in **a** from PhyloPic (http://phylopic.org/) under Creative Commons license CC0 1.0: *Bacillus subtilis*, *Retortamonas* and *Bdellovibrio bacteriovorus*, Guillaume Dera; *Rhizopus oryzae* and *Capitella capitata*, T. Michael Keesey; Philodinidae, Fernando Carezzano; *Diplonema papillatum*, Matus Valach; *A. queenslandica*, seung9park; *Ciona intestinalis*, Christoph Schomburg and Kurtis Wothe; *Tethyaster subinermis*, Birgit Lang; *Articulata*, Lauren Sumner-Rooney; *Ptychodera flava*, Albert Martí i Sabarí; *Strongylocentrotus purpuratus*, Christoph Schomburg; *Priapulus* and *Branchiostoma lanceolatum*, Yan Wong; *Rheobatrachus vitellinus*, Jose Carlos Arenas-Monroy; *Pyropelta* and *Tricolia pullus*, Tauana Cunha; *Choromytilus palliopunctatus*, Katie Collins; *Owenia*, Ludwik Gąsiorowski; *Lumbricus terrestris*, Carlo De Rito; *F. candida* and *Daphnia*, Kamil S. Jaron; *Myodocopa* and *Syncarida*, James Bernot; *Erebonectes*, Joanna Wolfe; *Bradysia coprophila*, Christina Hodson.

To functionally validate these predictions, we heterologously expressed PHADs from the sponge *Amphimedon queenslandica*, the earthworm *Lumbricus rubellus* and two PHADs from the springtail *Folsomia candida*. All four heterologously expressed enzymes degraded both PHB and the random copolymer PHB/PHV to HB monomers (Fig. 5 and Supplementary Figs. 12–14; for calculations see Supplementary Table 5). Together, these findings demonstrate that PHADs are broadly distributed across the animal kingdom and functionally capable of degrading sPHAs.

### Evolution and distribution of PHADs across eukaryotes

All metazoan PHADs form a clade with those from the two protist groups, with the protists forming early branching clades to the animal sequences (Fig. 5). There are two exceptions: PHAD homologues from two bdelloid rotifer species group with fungal and bacterial sequences (Supplementary Fig. 15). These sequences could have originated from recent horizontal gene transfer, which is common in bdelloid rotifers^62^, or represent contamination. The second exception are protist PHADs from *Nibbleromonas* sp. (Provora), which form a sister group to the animal subclades III and IV, but without support (bootstrap is 57.4%).

The phylogeny of metazoan PHADs largely mirrors organismal relationships at the phylum level and, in some cases, also at lower taxonomic levels (Fig. 5 and Supplementary Figs. 16–20). For example, molluscan PHADs form a monophyletic group with a clear separation between bivalves and gastropods, and chordate PHADs are likewise consistent with established phylogenetic relationships within Chordata (Supplementary Figs. 16 and 17). Deviations from organismal phylogeny are more frequent in phyla with species that encode multiple PHAD homologues, such as arthropods and annelids (Supplementary Figs. 18 and 19). These deviations from organismal relationships at both phylum and lower taxonomic levels may reflect ancient duplication and differential gene loss or poor resolution at deep nodes—limitations inherent to single-gene phylogenetic analyses^63,64^.

The sister-group relationship between protist and metazoan PHADs suggests that PHADs were already present in the last common ancestor of animals (Fig. 5). The deep-branching position of the sponge *A. queenslandica* PHAD, the only non-bilaterian sequence recovered, further supports this interpretation. The most likely explanation is that early animals inherited one or more PHAD homologues from protist ancestors, followed by diversification or loss in different lineages. Although the bootstrap support for the node uniting protist and metazoan PHADs is relatively low (43.8%), assuming parallel evolution would require several independent origins of similar PHADs, which seems less likely. Broader sampling of protist and non-bilaterian animal lineages will be needed to confirm this inference.

If PHADs were indeed inherited from protist ancestors, their persistence in diverse animal lineages probably reflects a continued advantage in exploiting sPHA-rich microbial food sources. All 81 animal species that have a PHAD probably acquire sPHA through their diet. Gutless oligochaetes digest their PHA-synthesizing symbionts, whereas other metazoans obtain PHA by feeding on sediment, soil or detritus, which are often rich in sPHA-producing microorganisms (Fig. 5 and Supplementary Table 7). For instance, earthworms ingest soil containing up to 4.3 µgC g^−1^ of native PHB^1^ and springtails (Collembola) feeding on microbe-rich detritus encode up to 14 PHAD homologues (for example, *F. candida*), potentially enabling degradation of diverse environmental sPHAs (Supplementary Text 4). Similarly, PHAD-harbouring protists such as Provora and bacteria from the phylum Bdellovibrionata (Supplementary Fig. 21) are predators of bacteria and would naturally encounter sPHA-producing prey^65–67^. These patterns collectively suggest that PHADs are common among organisms whose nutrition involves sPHA-rich microbial sources.

## Conclusions

Given the widespread occurrence of PHADs across protists and animals, their ability to degrade microbial sPHAs may represent an underappreciated process in global carbon cycling. However, data on sPHA concentrations and degradation rates in natural environments remain scarce, even though microorganisms from diverse habitats produce and use sPHA^1–5,9,29–32,68–70^. Moreover, intracellularly stored carbon compounds, such as sPHA, are often overlooked in estimates of microbial biomass, despite evidence that they contribute measurably to soil carbon pools^71^. Of the few studies available, reported concentrations of sPHA are in the range 1.2–4.3 µgC g^−1^ in soils^1,72^, equivalent to 0.001–0.16% of soil organic carbon^73,74^. In marine sediments, PHA concentrations were estimated at 140,000 ng g^−1^ of sediment in mangroves^75^, 140 ng g^−1^ of sediment in estuarine habitats^76^ and 2–60 ng g^−1^ of sediment in the deep sea^77^. However, these low values may underestimate true sPHA abundance, as most analytical methods are optimized for microbial cultures and fail to detect microbial produced sPHAs that occur in the environment^1,70^. In addition, rapid sPHA turnover in natural habitats would prevent its accumulation and therefore hinder detection. Importantly, degradation rates of sPHAs in terrestrial and aquatic environments remain completely unknown, as existing studies focus exclusively on industrial or experimental sPHA-based plastics, which differ structurally from naturally occurring sPHAs^78–83^.

In the Anthropocene, where plastic pollution has reached every ecosystem on Earth^84–87^, sPHAs stand out as one of the few bioplastics microorganisms both synthesize and degrade^88–91^. Our finding that animals also possess PHADs capable of degrading sPHAs incentivizes the use of sPHA in bioplastic production, which still represents less than 4% of the global bioplastic market^92^. For instance, earthworms, the most abundant annelids in soils globally^93^, are key soil decomposers^94^ and were shown to partially degrade the bioplastic polylactic acid^95^. Their newly recognized PHADs, as well as those of other soil inhabitants such as insects and aquatic invertebrates, indicate that these animals may be able to contribute to the full degradation of sPHA-based plastics.

In conclusion, our study identified PHADs as a new group of animal enzymes, and highlights how ubiquitous these proteins are in protists and animals from a wide range of environments. We further highlighted the value of exploring non-model organisms for expanding our knowledge of the functional diversity of eukaryotes. Future research should focus on developing a better understanding of sPHA synthesis and degradation in natural ecosystems, not only to better understand the contribution of these processes to the carbon cycle, but also to develop strategies for mitigating plastic pollution in natural ecosystems.

## Methods

### Sampling, extraction and sequencing

To generate the transcriptomes used in this study, scuba divers collected individual worms (*n* = 3–15) from 15 different gutless oligochaete species from marine sediments between 2015 and 2021 (Supplementary Table 3). The research was conducted in alignment with European Union guidelines for field-based research. The worms were sorted from the sediment and stored in RNAlater (Thermo Fisher Scientific) at −80 °C until nucleic acid extraction. We extracted RNA from individual worms using either Qiagen AllPrep DNA/RNA/Protein or DNA/RNA Mini Kits (Qiagen; Supplementary Table 3) with the following adjustments to the manufacturer’s protocol: β-mercaptoethanol was replaced by 20 µl of 2 M dithiothreitol and 1 µl of Reagent DX (Qiagen 19088), the bead mixture for tissue disruption consisting of a custom mixture of sterilized silicon beads (about 20 1.2-mm ZY-S Silibeads and about 5 2-mm ZY-Silibeads) and Matrix B silicon sand (MP Biosystems) and a FastPrep (MP Biomedicals) was used to disrupt tissues by bead beating (2 × 4 m s^−1^ for 40 s). Extracted RNA samples were eluted in 40 µl of DEPC-treated water and stored at −80 °C until library preparation.

Extracted RNA was sent to the Max Planck Genome Centre (Cologne, Germany) for library generation and sequencing. Host-enriched, polyA libraries were constructed using NEBNext Single Cell/Low Input RNA Library Prep Kit for Illumina (NEB). Total RNA libraries were generated with the NEBNext Ultra II Directional RNA Library Prep Kit for Illumina (NEB). All transcriptomic libraries were sequenced on an Illumina HiSeq3000 in paired-end read mode, resulting in 3.3 million to 3.5 million reads per library.

### Transcriptomic data processing

Raw transcriptomic reads were quality filtered to generate animal host and bacterial symbiont (total mRNA libraries only) enriched datasets from all gutless oligochaete species. BBDuk (v38.90; https://jgi.doe.gov/data-and-tools/software-tools/bbtools/) was used to trim reads from each dataset that contained adaptors, were shorter than 36 bp, had quality scores that dropped to zero and had *k*-mers less than 11 bp long at the sequence ends. The rRNA reads were removed from the quality-filtered data using SortMeRNA (v4.3.4)^96^. For *O. algarvensis* and *Olavius* *ilvae*, cleaned read sets were mapped to the partial host genome assemblies, which were assembled as reported in ref. ^97^, using BBMap (v 39.00)^98^ at 98% identity to separate host and symbiont reads with each transcriptomic dataset. For all other gutless oligochaetes, where we lacked access to high-quality genome assemblies, the quality-filtered transcriptomic reads were mapped at 98% identity to the known bacterial symbiont genomes to separate the reads into host and symbiont-enriched datasets (see Supplementary Text 7 for a description of how we obtained the symbiont genomes)^48^. Normalized reads were assembled de novo using Trinity (v2.5.1)^99^. The quality of the resulting assemblies were determined by calculating N50 and by assessing completeness using BUSCO (v 4.1.4)^100^. TransDecoder (v5.5.0; https://github.com/TransDecoder/TransDecoder/) was used to predict the protein-coding sequences from each transcriptomic assembly.

### PHAD and BHBD identification

Predicted *O. algarvensis* transcripts were searched against the PHAD engineering database^44^ using BLASTp (v 2.11.0; e-value of 1)^101^ to identify the PHAD sequence of the animal. The resulting sequence was subsequently used as reference to animal PHADs from all other host-enriched gutless oligochaete transcriptomes by comparing the predicted transcripts against the *O. algarvensis* PHAD using BLASTp. Resulting sequences with an e-value of <1 were compared with the National Center for Biotechnology Information (NCBI) non-redundant (nr) protein database^101,102^ to determine that the sequence had a closer match to other known eukaryotic proteins. We repeated this process to identify other gutless oligochaete PHAD sequences in the host-enriched transcriptome and bacterial PHAD sequences from the symbiont-enriched transcriptomic assemblies.

To determine if the gutless oligochaetes and their symbionts also transcribed the genes that use the products of sPHA degradation, we used HMMER (v3.1b2)^103^ to search the host- and symbiont-enriched transcriptome assemblies for a BHBD, beta-hydroxybutyrate oligomer hydrolase and 3-hydroxybutyryl-coenzyme A dehydrogenase (Supplementary Data 2). Transcriptome assemblies were compared against a custom database containing known bacterial genes involved in transforming the products of sPHA degradation. Sequences with matching hmmsearch scores <50 were subsequently cross- referenced to the nr-protein database on NCBI to confirm their identity.

To identify other animal and protist PHADs, we screened the publicly available databases: nr-protein database^101,102^, UNIPROT^104^, ENSEMBL^105^ and LumbriBASE^106^ for unannotated PHAD sequences. To extend the number of identified putative animal PHADs, we used a recursive screening approach to putatively identify distantly related animal PHADs. Briefly, the identified *O. algarvensis* PHAD sequence served as a query sequence to identify other unannotated PHAD sequences within both nr-protein database^101,102^ and UNIPROT^104^. The resulting sequence with the lowest identity to the seed was used to repeat the database search. This process was repeated for a total of ten cycles, at which point the search no longer identified animal PHADs. All putatively identified sequences were downloaded and dereplicated using dedupe.sh (v38.9) and aligned using the local pair alignment in MAFFT (v 7.407)^107^.

### Identification of the *phaZ* gene

To identify the gene sequence that encodes the PHAD in *O. algarvensis*, we used a TBLASTN (v 2.11.0)^101^ with the PHAD protein sequence as the query, to identify the *phaZ* gene in the *O. algarvensis* assembled genome. The intron and exon structure of the *phaZ* gene was predicted from the PHAD mRNA sequence using SPLIGN using low identity mode^108^.

### Gene expression analysis

We quantified gene expression in individual gutless oligochaetes from ten species to determine the relative abundance of PHAD transcripts in host-enriched transcriptomic libraries. First, using polyA transcriptomic libraries, we generated de novo co-assemblies from the host-enriched read set for each species independently using Trinity (v 2.5.1)^99^. Corresponding raw reads from individual libraries were then mapped to the species-specific co-assembly to generate read counts for each transcript using Kallisto (v 0.46.0)^109^. To annotate gutless oligochaete transcripts, we conducted a BLASTp search against a custom database consisting of either PHADs or genes involved in symbiont digestion previously identified in gutless oligochaetes transcriptome assemblies^39^. To compare the relative expression of the animal PHAD to other digestive enzymes and baseline transcription, we plotted the transcripts per kilobase million values of all expressed genes as violin plots on a log_10_ scale for each gutless oligochaete species.

### Metaprotemics analysis

The worms were prepared for metaproteomic analyses in four different ways. These included (1) single worms washed in 99% ethanol, analysed as three biological replicates (*n* = 3), (2) a pool of three worms washed in 99% ethanol (*n* = 3), (3) a pool of five worms washed in 99% ethanol (*n* = 3) and (4) untreated single worms analysed as three biological replicates (*n* = 3; Supplementary Table 4), resulting in a total of 12 metaproteomic samples analysed. Each worm was dissected to remove the head portion (‘tip’) from the body (‘trunk’) just above the genital pad region^110^ to collect metaproteomic datasets from symbiont-depleted (tip) and symbiont-enriched (trunk) regions. The individual tip and trunk samples were flash frozen in liquid nitrogen and stored at −80 °C before protein digestion. Samples were digested following the filter-aided sample preparation protocol adapted from ref. ^111^ (Supplementary Text 8). The extracted peptides were then measured by one-dimensional liquid chromatography coupled to mass spectrometry (MS). A custom database was generated using a *O. algarvensis* symbiosis protein sequence database (PRIDE PXD007510)^112^, supplemented with sequence annotations predicted in this study and protein sequences from the common repository of adventitious proteins (cRAP), which lists common contaminates in metaproteomics. Mass spectrometry/mass spectrometry (MS/MS) spectra were searched against this database using the Sequest HT node in Proteome Discoverer (v 2.5; trypsin (full), maximum two missed cleavages, 10 ppm precursor mass tolerance, 0.1 Da fragment mass tolerance and maximum of three equal dynamic modifications per peptide: oxidation on N (+15.995 Da), carbamidomethyl on C (+57.021 Da) and acetylation on the protein N terminus (+42.011 Da)).

### Primary structure analysis

To identify conserved and variable regions of the catalytic and substrate-binding domains of PHAD, we compared the recovered animal and protist PHAD amino acid sequences to the fungal homologue from *P. funiculosum* (PDB 2d80; 2d81). Sequences were aligned using a local pair alignment in MAFFT (v7.407)^107^. SignalP (v 6.0)^113^ was used to predict the signal peptides for each of the recovered enzymes. New animal and protist sequences that contained the complete catalytic site were predicted to be eukaryotic PHADs and were kept for downstream analyses.

### Homology modelling

The structure of each of the gutless oligochaete and selected metazoan enzymes was predicted using the monomer prediction function within AlphaFold2 (v2.1.1)^114,115^. PyMOL (v 2.4.0) was then used to visualize the predicted local distance difference test (pLDDT) calculated during the AlphaFold2 prediction. All models had pLDDT values >80, indicating a strong prediction. We aligned the AlphaFold2 models to the crystal structure of the PHAD from the fungus *P. funiculosum* (PDB 2d81) and calculated the root-mean-square deviation (r.m.s.d.) to determine the structural similarities between the animal and fungal homologues. Lastly, we predicted the fitting of a PHB in the catalytic site using the coordinates of the fungal PHAD.

### Phylogenetic reconstruction

To resolve the phylogeny of PHADs, the *O. algarvensis* host and symbiont PHADs were aligned with PHADs in the PHAD engineering database^44^ using the local pair alignment in MAFFT^107^ (v 7.407). The aligned sequences were used to calculate a maximum-likelihood tree with ultrafast bootstrap support values using IQ-TREE^116^ and visualized in iTOL (v 5 and 6)^117^. On the basis of the grouping of the PHADs from *O. algarvensis* and other gutless oligochaetes that have extracellular PHADs predicted to degrade short-chain PHAs from domain type 2, we aligned recovered animal and protist PHADs with the extracellular PHADs of domain type 2 from the PHAD engineering database using a local pair alignment in MAFFT (v 7.407)^107^. The alignment was used to calculate a maximum-likelihood tree with ultrafast bootstrap support values using IQ-TREE (v 1.6.12)^116^.

### Heterologous gene expression, enzyme purification and assays

To determine if the animal PHAD degraded sPHA, we expressed *O. algarvensis* PHAD and a homologue from *Paucimons lemoignei* (accession: P52090), which served as a positive control, in *E. coli*. The sequence was submitted to GenScript for delivery in a standard expression vector (pet28a(+)) with the sequences of interest inserted between the restriction sites NheI/XhoI, which resulted in a N-terminal hexahistidine taq in the recombinant protein. We transformed the *O. algarvensis* PHAD vector by heat shock in *E. coli* BL21 chemo competent cells (DE2; Thermo Fisher); the *P. lemoignei* vector was transformed into BL21 rosetta competent cells (DE3; Merck). To determine if we successfully inserted the PHAD expression vectors in *E. coli*, the *E. coli* clones were sent for plasmid extraction and Sanger sequencing (Microsynth AG). The obtained plasmid sequences were aligned with the PHAD sequences of *O. algarvensis* and *P. lemoignei* using MAFFT (v 7.407)^107^. Enzyme overexpression and purification were performed following the protocol of ref. ^118^. In short, after successful transformation, a preculture of transformed *E. coli* cells was prepared in 3 ml of LB medium and 1 ml of the preculture used to inoculate 1 l of LB medium. The cells were grown for 8 h at 37 °C, 1 ml of 1 M IPTG added, incubated overnight at 20 °C and harvested by centrifuging at 4,500*g* for 25 min at 4 °C. Proteins were extracted from the harvested cells by adding 10 ml of sucrose stock solution (250 g sucrose and 6 g of Tris in 1 l Milli-Q water), 5 mg of lysozyme, 30 ml of deoxycholate solution (10 g of sodium deoxycholate, 10 ml of Triton X-100, 5.8 g of NaCl, 2.4 g of Tris in 1 l Milli-Q water), 0.5 ml of DNase stock solution (50 mg of DNase I in 35 ml of immobilized metal affinity chromatography (IMAC) buffer which consisted of 29.2 g of NaCl, 2.4 g of Tris and 1.7 g of imidazole and 15 ml of glycerol), followed by vigorous shaking. Samples were then centrifuged at 16,000*g* for 45 min at 4 °C to separate soluble from insoluble protein fractions. Because the *O. algarvensis* PHAD was expressed in inclusion bodies, we included a refolding step (refolding buffer 20 mM Tris-HCl pH 8.0 and 2 M urea; washing buffer 20 mM Tris-HCl, 300 mM NaCl, 1 mM EDTA, 1% Triton X-100, 1 M urea, pH 8.0)^119^ and froze the sample overnight to extract the enzyme from the inclusions. The enzyme was then purified by IMAC using the pooled eluent fractions corresponding to the protein of interest. We exchanged the buffer by overnight dialysis using 6–8 kDa dialysis bags against SEC buffer (20 mM Tris, 0.5 M NaCl, pH 7.5) at 4 °C and stirred at 150 rpm before concentrating the enzyme by ultrafiltration.

For the animal PHADs from *A. queenslandica*, *F. candida*, *L. rubellus* and the symbiont PHAD, we used a cell-free expression system to heterologously produce the enzymes. Coding sequences were synthesized and cloned by GenScript into the standard expression vector pET23a(+), inserted between the restriction sites NheI and XhoI. Protein expression was carried out using the PURExpress Cell-Free Expression Kit with the PURExpress Disulfide Bond Enhancer (New England Biolabs), following the manufacturer’s instructions. Each reaction contained 250 ng of plasmid template and was incubated for 3 h.

To determine if the heterologously expressed animal PHAD degraded sPHA, we used a spot assay with polymer plates containing 0.5 mg ml^−1^ of the homopolymer PHB (Merck) and the copolymer PHB/PHV (Merck)^120^. The assay plates were prepared by adding either a stable suspension of PHB or PHB/PHV in 100 mM Tris-HCl (pH 8.5) to a 1.4% agar gel. A total 10 µl of the purified enzymes and heat-denatured 1:1 enzyme mix (negative control) or for the cell-free expression mix a non-sense vector were spotted on to the PHB and PHB/PHV plates. Enzyme activity was determined if a clearance zone formed after incubation at 37 °C for 16 h or 24 h.

To identify the degradation products of the animal PHADs, 50 mg of the clearance zone or the spot of the negative control were excised from the agar plate and transferred onto a glass wool filter (Carl Roth; 11 µm). To account for autohydrolysis during the assays, we also cut 50 mg of the polymer from the same assay plates (no-enzyme polymer control). The agar plug was liquefied by centrifugation at 15,000*g* for 1 min at 20 °C. The glass wool was then washed with 70% methanol and centrifuged again under the same conditions. Both extracts were pooled and concentrated in a vacuum concentrator after addition of internal standards (35 μl of mixed ribitol (0.0714 mg ml^−1^) and 5-α-cholestane (0.0286 mg ml^−1^); Sigma-Aldrich). The same procedure was performed for HB standards.

Dried samples were derivatized by adding 80 µl of methoxyamine hydrochloride dissolved in pyridine (20 mg ml^−1^) and incubating for 90 min at 37 °C with constant rotation (1,350 rpm). Pyridine was then removed under a gentle N_2_ stream for 35 min at 37 °C while shaking at 250 rpm. Subsequently, 100 µl of N,O-bis(trimethylsilyl)trifluoroacetamide was added to each sample, vortexed briefly and incubated for 30 min at 37 °C with constant rotation (1,350 rpm). Finally, 100 µl of the derivatized extract was transferred into GC–MS vials for data acquisition.

The metabolic profiles of each derivatized sample and standard were obtained on an Agilent 7890B GC coupled to an Agilent 5977 A single quadrupole mass selective detector. The injector temperature was set at 290 °C. Using an Agilent 7693 autosampler, 1 μl was injected in splitless mode through a GC inlet liner (ultrainert, splitless, single taper, glass wool, Agilent) onto a DB-5MS column (30 m × 0.25 mm, film thickness 0.25 μm; including 10-m DuraGuard column, Agilent). Metabolite separation on the column was achieved with an initial oven temperature of 60 °C followed by a ramp of 20 °C min^−1^ until 325 °C was reached, which was then held for 2 min. Helium carrier gas was used at a constant flow rate of 1 ml min^−1^. The mass spectra were obtained by electron ionization mode at 70 eV across the mass range of 50–600 *m*/*z* and a scan rate of two scans per second. The retention time was locked using standard mixture of fatty acid methyl esters (Sigma-Aldrich).

HB peaks were identified with the Mass Hunter Suite (Agilent Mass Hunter Quantitative Analysis v 10.2; Agilent Mass Hunter Qualitative Analysis v 10.0) through comparison to the NIST database and HB identification by the NIST database; identification was confirmed by comparing peak retention times and mass spectra to HB standards run on the same instrument. We further used the Mass Hunter Suite to quantify HB peaks. The HB peaks were normalized by the internal standards. We used mzXML (MsConvert)^121^ and XCMS (v.2.99.6)^122^ to plot chromatograms and mass spectra of the raw data in R (4.0.3) for which we used the *m*/*z* of 248.

### PHAD and symbiont imaging

To localize host and symbiont PHAD expression in *O. algarvensis*, we fixed individual worms after sampling them from their natural habitat in 4% paraformaldehyde (PFA) in phosphate buffered saline (PBS) for 4 h at 4 °C. Samples were stored in methanol at −20 °C before HCR-FISH following ref. ^123^. For the whole-mount hybridizations, we used custom-designed HCR-FISH probes that distinguished between the host and symbiont PHAD transcripts (Supplementary Table 10). We modified the protocol from ref. ^123^ as follows. Fixed worms were first dissected into ~5-mm pieces to allow better handling and rehydrated in PBS. To enable penetration of the probes, the tissues were digested with 0.05 mg ml^−1^ of proteinase K for 5 min at 37 °C. The reaction was stopped by washing the fragments twice for 5 min in 2 mg ml^−1^ of glycine in PBS with 0.01% Tween-20 on ice and subsequently refixing the fragments in 4% PFA for 60 min at room temperature. For the hybridization we used a 30% hybridization buffer (30% formamide, 5× sodium chloride sodium citrate (Invitrogen), 9 mM citric acid, 0.1% Tween-20, 50 µg ml^−1^ of heparin, 1× Denhardt’s solution, 10% dextran sulfate) in which we pre-incubated the worm pieces for 15 min on ice, 5 min at room temperature and 30 min at 37 °C. The HCR-FISH probes for host and symbiont PHADs were added at a final concentration of 8 nmol to the hybridization buffer. We performed pre-amplification by incubating the samples 2 × 30 min in amplification buffer (5× concentrated saline sodium citrate (Invitrogen) buffer (3.0 M NaCl and 0.3 M sodium citrate, pH 7.0; Invitrogen), 0.1% Tween-20, 10% dextran sulfate) at room temperature before adding the hairpins to a final concentration of 30 pmol. The samples were then incubated overnight at room temperature. A B1 initiator sequence for the hairpin with the fluorophore 546 was used for the host PHAD, while a B3 initiator sequence for the hairpin with the fluorophore 647 was used for the symbiont PHAD. The samples were counterstained in 2 µM DAPI, mounted in Electron Microscopy Sciences Citifluor and visualized as single planes using confocal microscopy (Zeiss LSM 780 with Airyscan and ELYRA PS.1; ZEN v 2.3).

To localize *Ca.* T. algarvensis within host tissues, we used multilabelled-FISH on individual fixed worms prepared for HCR-FISH, with a gammaproteobacterial specific probe (Gam42a)^124^. Fixed worms were first dissected into ~5-mm pieces and the pieces rehydrated in PBS and permeabilized and refixed as described above for the HCR-FISH protocol. The worm pieces were hybridized in a buffer containing 900 mM NaCl, 20 mM Tris-HCl (pH 7.5), 0.01% sodium dodecyl sulfate (SDS) and 30% formamide to which the Gam42a probe was added at a concentration of 8.4 pmol. The hybridization was carried out at 46 °C overnight after two prehybridization step in just the hybridization buffer without the probe for 10 min at room temperature and 3 h at 46 °C. Samples were then washed in hybridization buffer at 46 °C for 10 min and 30 min and then at 48 °C two times in a wash buffer containing 112 mM NaCl, 20 mM Tris-HCl (pH 8), 5 mM EDTA (pH 8) and 0.01% SDS. The samples were then counterstained in 2 µM DAPI, mounted in Electron Microscopy Sciences Citifluor and visualized as single planes using confocal microscopy (Zeiss LSM 780 with Airyscan and ELYRA PS.1; Zen v 2.3).

High-voltage transmission electron microscopy of *O. algarvensis* epithelial cells was performed on individual worms that were preserved in 2% PFA/0.25% glutaraldehyde. After fixation, worms were dehydrated in ascending ethanol series and then embedded in LR White resin (Electron Microscopy Sciences). Embedded worms were cut into ultrathin sections on a Leica UC7 ultramicrotome (Ultracut UC7 Leica Microsystem). The sections were subsequently mounted on gold plates and contrasted with uranyl acetate (Science Services) for 20 min and with 2% Reynold’s lead citrate for 6 min. The contrasted sections were imaged at 10 kV in high vacuum mode at a magnification of ×10.767, a horizontal field width of 38.5 µm and a working distance of 9.5 mm with a Quanta FEG 250 scanning electron microscope (FEI Company) equipped with a scanning transmission electron microscope detector using the xT microscope control software (version 6.2.6.3123).

### Reporting summary

Further information on research design is available in the Nature Portfolio Reporting Summary linked to this article.

## Supplementary information

Supplementary Information Supplementary Text 1–9 and Figs. 1–24.

Reporting Summary

Peer Review File

Supplementary Tables Supplementary Tables 1–10.

Supplementary Data 1 PHAD sequences of host and symbionts.

Supplementary Data 2 Reference list of PHA degradation sequences.

## Data Availability

The sequencing data for this study have been deposited in the European Nucleotide Archive (ENA) at EMBL-EBI under accession numbers PRJEB72699 (total RNA libraries), PRJEB52460 (PolyA libraries)^97^, PRJEB94555 (Illumina reads)^48^ and PRJEB52678 (PacBio reads)^97^. The PHAD protein alignment and the original tree file is available via figshare at https://doi.org/10.6084/m9.figshare.26005156 (ref. ^125^). The protein models are available via figshare at https://doi.org/10.6084/m9.figshare.26005165 (ref. ^126^) for gutless oligochaetes and at https://doi.org/10.6084/m9.figshare.26003857 (ref. ^127^) for animals. Imaging data are available via figshare at https://doi.org/10.6084/m9.figshare.26005162 (ref. ^128^) and enzyme assay results are available via figshare at https://doi.org/10.6084/m9.figshare.26003860 (ref. ^129^). Calculations for the enzyme assays can be found in Supplementary Table 5. The mass spectrometry proteomics data have been deposited to the ProteomeXchange Consortium via the PRIDE partner repository with the dataset identifier PXD072931.
