# Supplementary material for: Animal degradation of microbial storage polyhydroxyalkanoates
Source: Nat Ecol Evol. 2026 Aug 13;10(10):1823–37. doi: 10.1038/s41559-026-03153-8 (PMC13642032; doi:10.1038/s41559-026-03153-8)
Supplement: Supplementary file 1 [file 41559_2026_3153_MOESM1_ESM.pdf]

nature ecology & evolution

Supplementary information https://doi.org/10.1038/s41559-026-03153-8

Animal degradation of microbial storage polyhydroxyalkanoates

In the format provided by the authors and unedited

Supplementary Text

Supplementary Text 1 | Primary structure and functional analysis of the *Thiosymbion algarvensis* PHAD

To further investigate the predicted function of the PHAD encoded by the symbiont *Thiosymbion algarvensis*, we performed a detailed primary structure and functional comparison with experimentally validated PHADs. A search against the PHAD Engineering Database (DED) revealed that the *T. algarven*sis PHAD most closely aligned with the experimentally validated domain type 1 dPHADscl from the bacterium *Ralstonia pickettii* (identity: 11.4%, coverage: 52.8%, similarity: 62%). Within the catalytic site, the *T. algarvensis* PHAD contains the same catalytic serine and asparagine residues but lacks the histidine residue present in *R. pickettii.* The oxyanion hole is conserved between the two enzymes and positioned before the lipase box.

While the catalytic sites of the two PHADs are conserved, regions involved in substrate interaction differ substantially. The *T. algarvensis* PHAD lacks the fibronectin-type-III–like linker domain present in *R. pickettii* (A370–S427), which connects the catalytic domain to the substrate-binding domain[1]. In addition, there was no detectable sequence similarity between the substrate-binding domains of the *R. pickettii* and *T. algarvensis* PHADs. This divergence suggests that the *T. algarvensis* PHAD either engages with PHA through a distinct binding mechanism or has a substrate-binding site that has not yet been described. Besides differences in the substrate-binding sites, the two PHADs also differ in their predicted secretion. While the *R. pickettii* PHAD has an N-terminal signal peptide spanning the first 27 amino acids, no signal peptide was predicted in the *T. algarvensis* homolog. Domain predictions of the *T. algarvensis* PHAD suggest that both the N-terminal region of the enzyme and its catalytic triad are located inside of the cell. These regions are followed by a short (10 amino acid) transmembrane domain and a C-terminal region exposed to the exterior of the cell. Together, the predicted localization and sequence structure supports a membrane-associated localization of the *T. algarvensis* PHAD rather than secretion as an extracellular PHAD.

To test whether the *T. algarvensis* PHAD is catalytically active, we conducted heterologous expression assays using the symbiont enzyme. The PHAD degraded both polyhydroxybutyrate (PHB) and the random copolymer PHB/polyhydroxyvalerate (PHV) into hydroxybutyrate (HB) monomers (Figure 2; Supplementary Figures 2, 13, and 22), confirming that it is a functional PHAD. In spot assays on PHB and PHB/PHV, the symbiont

PHAD produced 0.47–6.55 nmol HB mg protein^{-1}, compared to 1.11–2.23 nmol HB mg protein^{-1} produced by the host enzyme. Importantly, the ability of the *T. algarvensis* PHAD to degrade denatured sPHAs *in vitro* does not imply an extracellular function, as similar activities have been reported for experimentally validated intracellular PHADs[2, 3]. Taken together, the primary structure, predicted cellular location, and functional assays support the interpretation presented in the main text that the *Thiosymbion* PHADs are misannotated as extracellular enzymes based on sequence similarity, despite functioning as an intracellular PHAD required for sPHA metabolism.

Supplementary Text 2 | *Olavius algarvensis* PHAD degrades short chain PHAs

To gain insight into the structural basis of PHAD activity in gutless oligochaetes, we generated a structural model of the *Olavius algarvensis* PHAD using AlphaFold2, comparing its predicted structure against the complete AlphaFold Protein Structure Database. The closest experimentally validated and structurally characterized homolog with the highest sequence identity within the same domain was a fungal PHAD from *Penicillium funiculosum* (PDB 2d81; predicted local distance difference test (pLDDT) = 94; Figure 2b)[4]. Although overall sequence identity between the two PHADs was relatively low (31.6 %), structural superposition revealed a high degree of similarity, indicating strong structural homology (root-mean-square deviation (RMSD) of 0.736 Å based on 236 Cα; DALI Z = 45.8).

Consistent with the fungal enzyme, the *O. algarvensis* PHAD was predicted to fold into a single-domain enzyme. The predicted active site comprised a canonical catalytic triad formed by residues S_{27}-D_{128} and H_{161}, as well as an oxyanion hole of S_{28} and C_{257} residues that were closely aligned with those of the fungal homolog (RMSD = 0.047-0.127 Å), supporting a conserved catalytic mechanism. Together with the strong overall structural similarity, this suggests that the *O. algarvensis* enzyme, like microbial PHADs, cleaves sPHA via a nucleophilic attack on the ester bond between the hydroxyalkanoate monomers[4, 5].

Although the substrate-binding region of the *O. algarvensis* enzyme shared only 28.6 % sequence similarity with the corresponding region of the fungal homolog, its tertiary structure was highly conserved (RMSD = 0.081-0.393 Å). In particular, the positions of residue W_{304}, which holds the polymer chain for the nucleophilic attack, and V_{129} and P_{298}, which flank the catalytic crevice and contribute to substrate attraction, are identical in the

animal and fungal homolog. These conserved structural features support efficient binding and processing of sPHAs.

Signal peptide prediction using SignalP[6] identified an N-terminal signal peptide forming the first 19 amino acids of the *O. algarvensis* PHAD. This signal peptide is predicted to mediate Sec-dependent export of the enzyme to the symbiont region beneath the worm's cuticle.

In addition to generating models for *O. algarvensis*, we also used AlphaFold2 to predict the structures of PHADs from nine other gutless oligochaete species (pLDDT = 85-96; Supplementary Figure 8b). All predicted PHADs had the same overall tertiary structure, including complete conservation of the catalytic triad, and key residues of the substrate binding domain. Several of these PHADs also contained predicted signal peptides consistent with Sec-dependent secretion (Supplementary Figure 8a; Supplementary Table 2). Together, these structural features indicate that PHADs from gutless oligochaetes are adapted to bind and degrade denatured extracellular sPHAs.

Supplementary Text 3 | Metaproteomic detection of PHAD expression

To assess PHAD expression *in vivo*, we analyzed metaproteomic datasets from two body regions of *O. algarvensis*, the *Thiosymbion*-free anterior tip of the worms and the *Thiosymbion*-rich postgenital trunk region which harbors symbiont-synthesized sPHA[7]. Extracted peptides of pooled and single worms were analyzed by metaproteomics[8, 9] (Supplementary Table 4, and Supplementary Text 8 below for method details).

We detected two unique peptides corresponding to the host PHAD in the trunk region of both pooled and single worms (n = 8 out of 12 samples, see Supplementary Text 8). In contrast, we did not detect peptides corresponding to the symbiont-encoded PHAD. This absence may reflect the low abundances of symbiont proteins relative to host proteins, or low expression levels of the symbiont PHAD, rather than its complete lack of expression. Consistent with this interpretation, expression of the symbiont PHAD was detected at the transcript level by mRNA hybridization (see Figure 4), indicating that the gene is expressed *in vivo* despite its absence in our metaproteomics dataset.

The detection of host PHAD specifically in the *Thiosymbion*-rich trunk region provides independent *in vivo* support for the role of the host enzyme in degrading symbiont-produced sPHAs. Together with the structural and functional analyses presented above, these

metaproteomic data further support host-mediated mobilization of symbiont-derived carbon stores.

Supplementary Text 4 | Multiple PHADs and potential metabolic flexibility

Bacteria encoding multiple PHAD copies can exhibit increased metabolic flexibility, as different PHADs generate distinct degradation products from sPHAs. For example, the betaproteobacterium *Ralstonia eutropha* has nine different intracellular PHADs that either release Co-A-bound monomers[10, 11], or hydrolyze sPHA into hydroxycarboxylic acid monomers or oligomers[12, 13]. These alternative degradation products can subsequently enter different metabolic pathways, thereby expanding the range of accessible substrates and metabolic outputs available to the organism.

In our dataset, several animal species also encoded multiple PHAD homologs. By analogy to bacterial PHADs, we hypothesize that animals with multiple PHADs may similarly exhibit increased metabolic flexibility, either by degrading different types of sPHA or generating distinct degradation products. To explore this hypothesis at a structural level, we modeled four PHADs from the water flea *Daphnia magna* using AlphaFold2 and compared their predicted structures. Relative to the fungal homolog from *P. funicolosum*, all four PHADs from *D. magna* show complete conservation of the catalytic triad, oxyanion hole and the tryptophan residue ($W_{302/317/336}$) that holds the polymer chain during degradation. These conserved features are consistent with all four enzymes functioning as PHADs.

In contrast, structural differences among the *D. magna* PHAD homologs are concentrated in the predicted substrate-binding region. Each homologue has a single amino acid substitution that is likely to influence substrate specificity. Notably, the two residues immediately following the catalytic aspartate ($D_{139/123/157}$) are substituted by either $SV_{140\text{-}141}$ or $ST_{124\text{-}125/158\text{-}159}$ across the four enzymes (Supplementary Figure 23). These substitutions may affect interactions with different sPHA substrates. However, direct biochemical assays will be required to determine substrate specificity and degradation products of individual PHAD homologs and to test whether PHAD multiplicity increases metabolic flexibility.

Supplementary Text 5 | Gutless oligochaetes express enzymes required for gaining energy from sPHA

Following depolymerization of symbiont-derived sPHAs by the *O. algarvensis* PHAD, additional enzymes are required to further catabolize the resulting breakdown products for carbon and energy generation. In bacteria, sPHA-derived oligomers are hydrolyzed to monomers by a hydroxybutyrate-dimer hydrolase (EC 3.1.1.22), and the resulting hydroxybutyrate monomers are subsequently oxidized to acetoacetate by a beta-hydroxybutyrate dehydrogenase (BHBD; EC 1.1.1.30). Acetoacetate can then be converted to acetyl coenzyme A (acetyl-CoA) and funneled into the citric acid cycle for energy production (Supplementary Figure 24)[14-22].

In animals, no homologs of the hydroxybutyrate-dimer hydrolase are known. However, animals encode BHBDs, which would allow oxidation of sPHA-derived hydroxybutyrate monomers to acetate[23]. To assess whether downstream sPHA catabolism is present in the host and symbiont, we applied the same gene identification and expression analysis strategy used for PHADs to enzymes involved in sPHA-derived carbon metabolism (see Methods: PHAD and BDBH identification). Specifically, we searched host and symbiont genomes and transcriptomes from all 15 host species examined for homologs of three enzymes known to catalyze downstream oxidation of hydroxyalkanoate intermediates: β-hydroxybutyrate dehydrogenase (BHBD; EC 1.1.1.30), hydroxybutyrate-dimer hydrolase (EC 3.1.1.22), and 3-hydroxybutyryl-CoA dehydrogenase (EC 1.1.1.157).

Across all analyzed *Thiosymbion* genomes and transcriptomes, we found no evidence for any of these three enzymes (Supplementary Table 6). Because at least one of these enzymes is required to oxidize sPHA-derived intermediates and generate reducing equivalents, their absence documents that *Thiosymbion* lacks the canonical enzymatic machinery required for downstream sPHA catabolism.

In contrast, host-enriched transcriptomic libraries encoded BHBD. In *O. algarvensis*, BHBD transcripts were detected at expression levels comparable to those of the host PHAD and other enzymes involved in symbiont digestion in the epidermis (Figure 4). Extending this analysis to additional gutless oligochaete species, we recovered BHBD transcripts from eight of the ten species for which host-enriched transcriptomes were available, with expression levels similar to those of their respective PHADs (Figure 4).

Together, these results document a consistent host-symbiont partitioning of sPHA metabolism across gutless oligochaetes: symbionts retain sPHA synthesis and depolymerization capacity, whereas hosts encode and express the downstream enzymatic machinery required to

degrade sPHA and oxidize sPHA-derived intermediates to capture symbiont-derived carbon.

Supplementary Text 6 | Protist PHADs

In addition to our discovery that PHADs are widespread in animals, we also identified homologs in 21 protist species spanning five major protist supergroups (Supplementary Table 8). Previously, PHADs had been reported only in the Amoebozoa *Acanthamoeba castellanii,* and the Evosea *Dictyostelium discoideum*[24]. Our analyses revealed that PHADs are also present in Choanoflagellates, Euglenozoa, Ciliophora, and Heterolobosea.

Phylogenetic analyses revealed that PHADs from Ciliophora formed a sister clade to Evosea PHADs, and to a larger clade containing sequences from Evosea, Choanoflagellate and Euglenozoa (Supplementary Figure 21). Overall, the phylogeny of protist PHADs did not recapitulate current protistan relationships[25]. For example, the PHADs of Choanoflagellata were not closely related to metazoan PHADs, but instead formed a sister clade to the PHAD from *A. castellanii* (Amoebozoa), while metazoan PHADs clustered with PHADs from Heterolobosea. This phylogenetic incongruence may reflect several non-mutually exclusive factors, including under sampling of protist PHAD diversity, the paraphyletic nature of several major protist groups[25], and limited phylogenetic resolution at deep-branching nodes of the PHAD tree.

Supplementary Text 7 | Binning of symbiont genomes

To generate reference *Thiosymbion* genome bins in this study, we used metagenomic data sets previously published under accession PRJEB94555[26]. The raw metagenomic reads were processed using BBDuk (v38.90; https://jgi.doe.gov/data-and-tools/software-tools/bbtools/), which was used for adapter removal, end trimming based on k-mer content, and filtering of reads shorter than 51 bp or with quality scores dropping to zero. Trimmed reads were error-corrected using BayesHammer (SPAdes v3.12.0.; error correction mode only)[27]. Read quality was subsequently assessed with FastQC[28], and taxonomic profiles of the samples were generated using phyloFLASH (v3.4)[29]. Quality-controlled reads were assembled using SPAdes (v3.12.0)[30] in metagenomic mode, with k-mer lengths of either 99 or 127 and a PHRED quality offset of 33. Assembled contigs were mapped against a custom database comprising symbionts from gutless oligochaetes using BBMap (v39.00)[31].

Metagenome assembled genomes (MAGs) were generated using MetaBAT (v2.0)[32], with a minimum contig length threshold of 1500 bp. For downstream analyses, we retained only MAGs from *Thiosymbion* that met the following criteria: estimated completeness between 52 % and 100 % with less than 1 0% contamination as determined by checkM (v2.0;)[33]. In addition, only bins from which 16S rRNA gene sequence could be recovered using Barrnap (version 9.0; https://github.com/tseemann/barrnap) were considered.

Supplementary Text 8 | Methods for metaproteomic analyses

The worms were prepared for metaproteomic analyses in four different ways. These included (i) single worms washed in 99 % ethanol, analysed as three biological replicates (n = 3), (ii) a pool of three worms washed in 99 % ethanol (n = 3), (iii) a pool of five worms washed in 99 % ethanol (n = 3), and (iv) untreated single worms analysed as three biological replicates (n = 3; Supplementary Table 4), resulting in a total of twelve proteomic samples analysed. The worms were dissected to separate the anterior tip region from the posterior trunk region just before the genital pads[7]. Dissected tip and trunk samples were flash frozen in liquid nitrogen and stored at -80 °C prior to protein digestion.

Samples were digested following the filter-aided sample preparation (FASP) protocol adapted from[34]. Briefly, 30 µl of SDT-lysis buffer (4 % (wt/vol) SDS, 100 mM Tris-HCl (pH 7.6), 0.1 M dithiothreitol) was added to each sample, followed by incubation for 10 min at 90 °C. Samples were centrifuged for 5 min at 21,000 x g and transferred to 10 kDa molecular weight cutoff centrifugal filter units (500 µl). To remove detergents, 200 µl of UA solution (8 M urea in 0.1 M Tris/HCl (pH 8.5)) was added to the filter units and the mixture centrifuged for 40 min at 14,000 x g. This step was repeated once. Alkylation was performed by adding 100 µl of IAA solution (0.05 M iodoacetamide in UA solution), mixing at 600 rpm for 1 min and incubating without mixing for 20 min at 22 °C in the dark. Filter units were centrifuged for 30 min at 14,000 x g and washed three times with 100 µl of UA by centrifugation for 20 min at 14,000 x g, followed by three washed with 50 mM ammonium bicarbonate using the same centrifuge conditions.

Filter units were transferred to fresh collection tubes and proteins were digested overnight at 37 °C in a humified chamber with 0.8 µg of Pierce MS-grade trypsin (Thermo Fisher Scientific) in 40 µl of ammonium bicarbonate buffer. Peptides were recovered by centrifugation at 14,000 x g for 20 min, followed by an additional elution with 50 µl of 0.5 M NaCl, mixing for 600 rpm for 1 min and centrifugation at 14,000 x g for 20 min. The eluted

peptides were quantified using the Pierce Micro BCA Protein Assay Kit (Thermo Fisher Scientific) following the manufacturer's instructions.

Peptide extracts were analyzed by one-dimensional liquid chromatography coupled to tandem mass spectrometry (1D-LC-MS/MS; Chromeleon v 7.2.10). Samples were loaded in solvent A (98% uHPLC water, 2% acetonitrile, 0.05% trifluoriacetic acid) onto a 300 µm inner diameter x 5 mm trap column packed with Acclaim PepMap100 C18, 5 µm 100 Å (Thermo Fisher, 174500) using an Ultimate 3000 RSLCnano Liquid Chromatograph (Thermo Fisher Scientific).

Peptides were separated on a 70 µm x 50 cm analytical EASY-Spray column packed with PepMap RSLC C18, 2 um material, 100 Å (Thermo Fisher Scientific, ES803), heated to 35 °C. The column was connected via an Easy Spray source to a Q Exactive HF-X Hybrid Quadrupole-Orbitrap mass spectrometer (Thermo Fisher Scientific). Peptides were eluted at a flow rate of 300 nl min-1 using a 140 min gradient, increasing from 95% buffer A (uHPLC water, 0.1% formic acid) to 50% buffer B (20% uHPLC water, 0.1% formic acid, 80% acetonitrile) over 102 minutes, followed by an increase to 99% buffer B over 38 minutes.

Eluting peptides were ionized by electrospray ionization (ESI) and analyzed in data-dependent acquisition mode. Full MS scans were acquired in the Orbitrap at a resolution of 70,000. The 15 most abundant precursor ions were selected for fragmentation, isolated with a 1.2 m/z window, fragmented by higher-energy collisional dissociation with a normalized collision energy of 30, and analyzed at a resolution of 17,500. Singly charged ions were excluded from MS/MS analysis, and dynamic exclusion was set to 25 s.

A custom protein sequence database was generated using a previously published *O. algarvensis* symbiosis database (PRIDE accession number PXD007510[35]) that was updated according to a metaproteomics database curation guide[36]. The database was refined using the cd-hit clustering approach[37] with improved symbiont and host sequence annotations including the PHAD protein predictions generated in this study. The final database comprised 1,439,552 protein sequences including symbiont and host proteins and the common Repository of Adventitious Proteins (cRAP; http://www.thegpm.org/crap) to represent common laboratory contaminants. MS/MS spectra were searched against the database using the Sequest HT node in Proteome Discoverer vs. 2.5 (Thermo Fisher Scientific). The Precursor Detector node was set with an S/N threshold of 1.5 and search parameters included trypsin (full), a maximum of two missed cleavages, and a fragment mass tolerance of 0.6 Da. Dynamic modifications acetylation of protein N termini (+42.011 Da) and included oxidation

of methionine (+ 15.995 Da) and deamidation (+0.984 Da) of Asparagine, Glutamine, and Arginine, with a maximum of 3 equal dynamic modifications per peptide. The static modification was carbamidomethylation of cysteine (+ 57.021 Da).

False discovery rates (FDRs) for peptide-spectrum matches (PSMs) were calculated using the Percolator Node in Proteome Discoverer, with a maximum delta Cn 0.05, a strict target FDR of 0.01, and a relaxed target FDR of 0.05, validated based on q-values. Protein-level FDRs were determined using the Protein FDR Validator Node with a target-decoy strategy. Proteins with q-values <0.01 were classified as high-confidence identifications, while proteins with q-values <0.05 were categorized as medium-confidence identifications.

Supplementary Text 9 | Phylogenetic analyses of animal PHADs relative to lipases and cutinases

To resolve the phylogenetic relationship between animal PHADs and lipases and cutinases, we clustered PHAD sequences with enzyme sequences retrieved from the ESTHER database[38]. Domain type 2 PHADs - including all animal PHADs identified in this study - were aligned separately with animal lipase and cutinase sequences using MAFFT[39] (v7.407) with the localpair option. Aligned sequences were manually inspected for conservation of the catalytic residues and the lipase box characteristic of α/β hydrolases. The resulting alignments were used to infer maximum likelihood trees using IQ TREE (v 1.6.12)[40], with branch support assessed by ultrafast bootstrap analysis. Phylogenetic trees were visualized using iTOL (version 5 & 6)[41] (Supplementary Figures 10 and 11).

Supplementary Figures

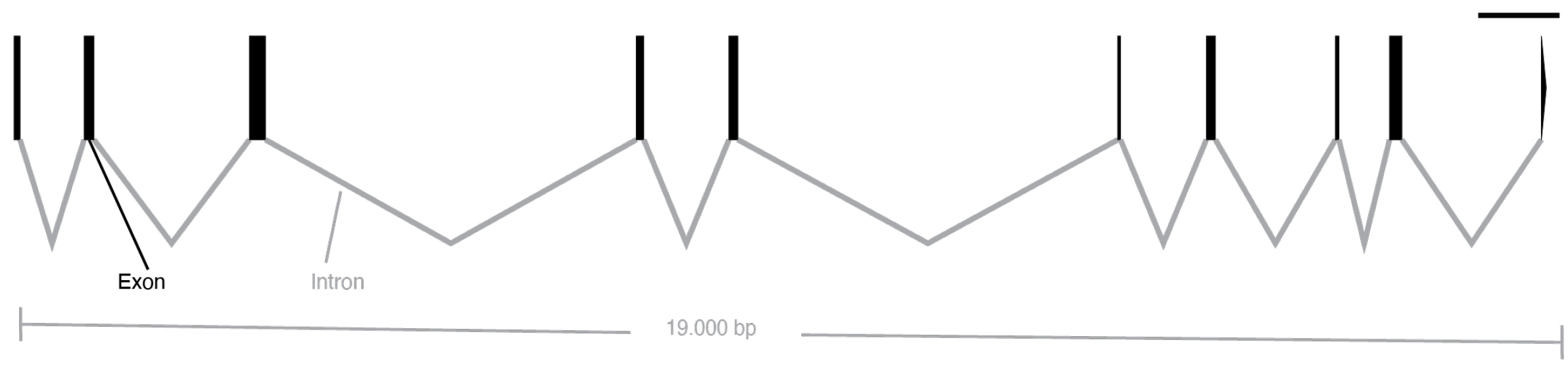

Supplementary Figure 1 | Identification of a *phaZ* gene encoding a PHAD in *O. algarvensis*. The *phaZ* gene identified in the *O. algarvensis* draft genome spans approximately 19 kb and is composed of 10 exons (black bars). Scale bar = 1 kb.

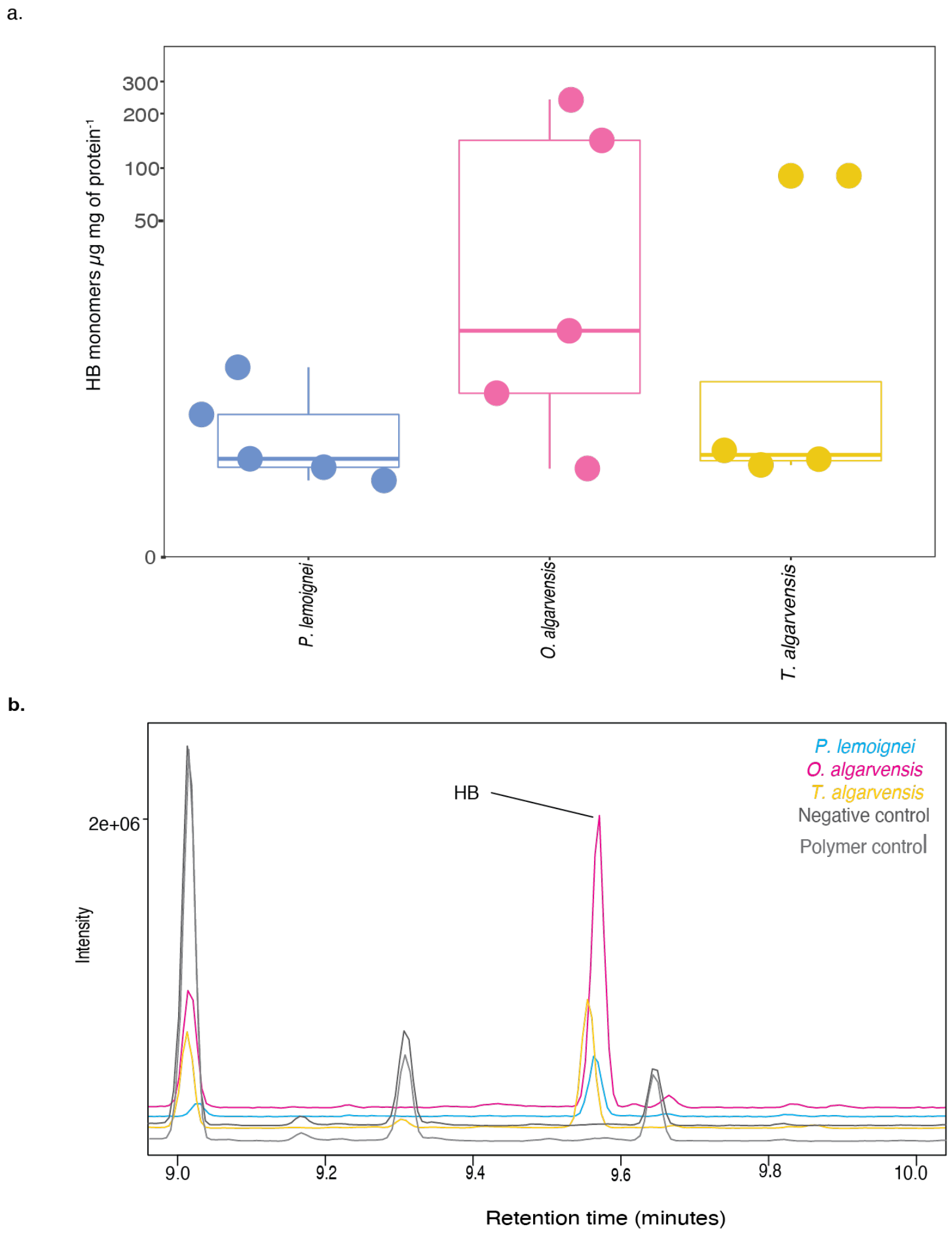

Supplementary Figure 2 | *O. algarvensis* PHAD degrades PHB/PHV into hydroxybutyrate (HB) monomers. a, b, GC-MS analyses of PHAD assays using PHB/PHV (n = 5 biological replicates of one heterologous expression). **a,** Boxplots show the concentration of HB monomers as a function of enzyme activity. The *O. algarvensis* PHAD (pink; n = 5 biological replicates of one heterologous expression, upper whisker = 14.9 nmol/mg protein, lower whisker = 0.2 nmol/mg protein; Q1 = 0.4 nmol/mg protein; Q3 = 12.1 nmol/mg protein; Median = 5.9 nmol/mg protein) produced more HB monomers than its symbiont (*T. algarvensis*, yellow; n = 5 biological replicates of one heterologous expression, upper whisker = 6.6 nmol/mg protein, lower whisker = 0.5 nmol/mg protein; Q1 = 0.9 nmol/mg protein; Q3 = 0.3 nmol/mg protein; Median = 0.2 nmol/mg protein) and the bacterial PHAD used as a positive control (*P. lemoignei,* blue; n = 5 biological replicates of one heterologous expression, upper whisker = 0.4 nmol/mg protein, lower whisker = 0.1 nmol/mg protein; Q1 = 0.1 nmol/mg protein; Q3 = 4.3 nmol/mg protein; Median = 1.8 nmol/mg protein). **b,** Total ion chromatograms show the relative peak heights of the HB monomers in representative assays (9.56 min). No HB was detected in assays using a heat inactivated PHAD (dark gray) or the controls without the enzyme (light gray, Supplementary Figure 3). Hydroxyvalerate (HV) monomers were not detected because the PHB/PHV substrate contained only 3% HV, which was below the detection limit of this assay.

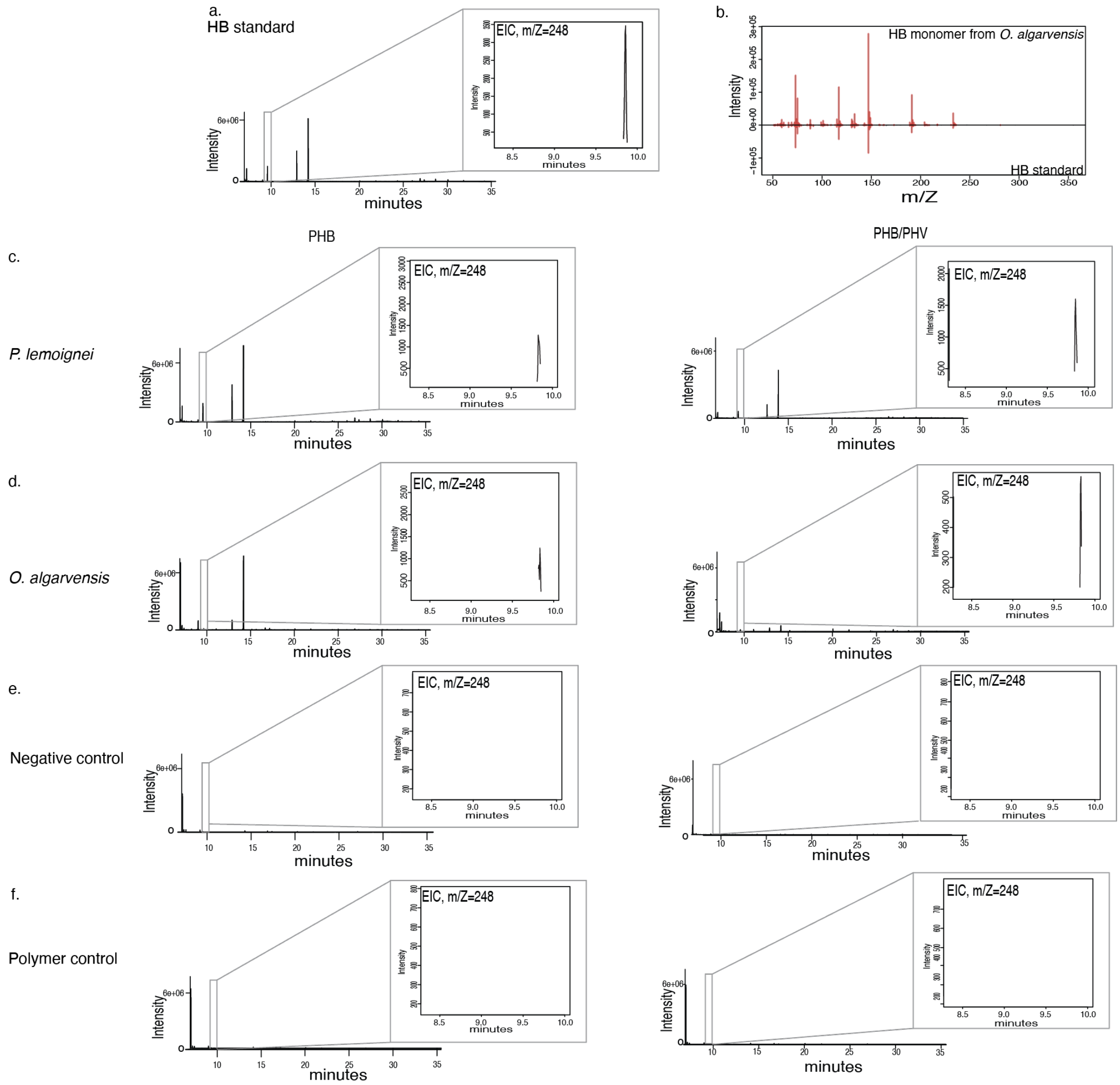

Supplementary Figure 3 | GC-MS identification of HB monomers from PHAD assays of PHB and PHB/PHV. a, Total ion chromatogram (TIC) of the HB standard showing a retention time of 9.56 min. The inset shows the extracted ion chromatograph (EIC) of the HB standard (m/z 248). **b,** Mass spectra demonstrating that HB produced in spot assays with heterologously expressed *O. algarvensis* PHAD match the mass spectra of the HB standard**. c, d,** TICs of PHAD assays using PHB and PHB/PHV for **c,** *P. lemoignei* and **d,** *O. algarvensis***.** Insets show EICs corresponding to the HB peak. **e, f,** No HB is detected in assays using **e,** heat inactivated PHADs or **f,** controls without the enzyme.

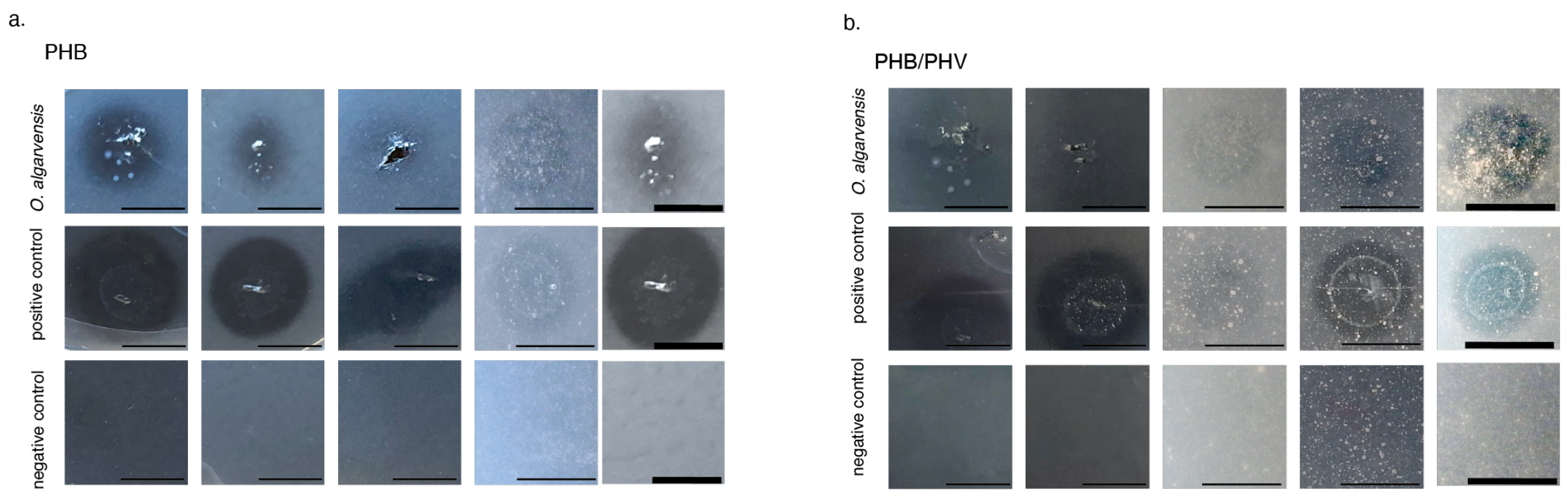

Supplementary Figure 4 | *O. algarvensis* PHAD is active on PHB and PHB/PHV substrates. Replication of enzyme spot assays (n = 5 biological replicates of one heterologous expression) on **a**, PHB and **b,** PHB/PHV assay plates. The heterologously expressed PHAD from *O. algarvensis* degrades sPHA within 24 hours, producing visible clearance zones. Clearance zones generated by the animal PHAD are less pronounced than those produced by the positive control, a heterologously expressed PHAD from the bacterium *Paucimonas lemoignei*, possibly reflecting differences in enzymatic activity between these two PHADs. Heat inactivation of both bacterial and animal PHADs eliminates their activity (negative controls). PHB/PHV assay plates show an uneven polymer distribution due to the reduced solubility of the copolymer.

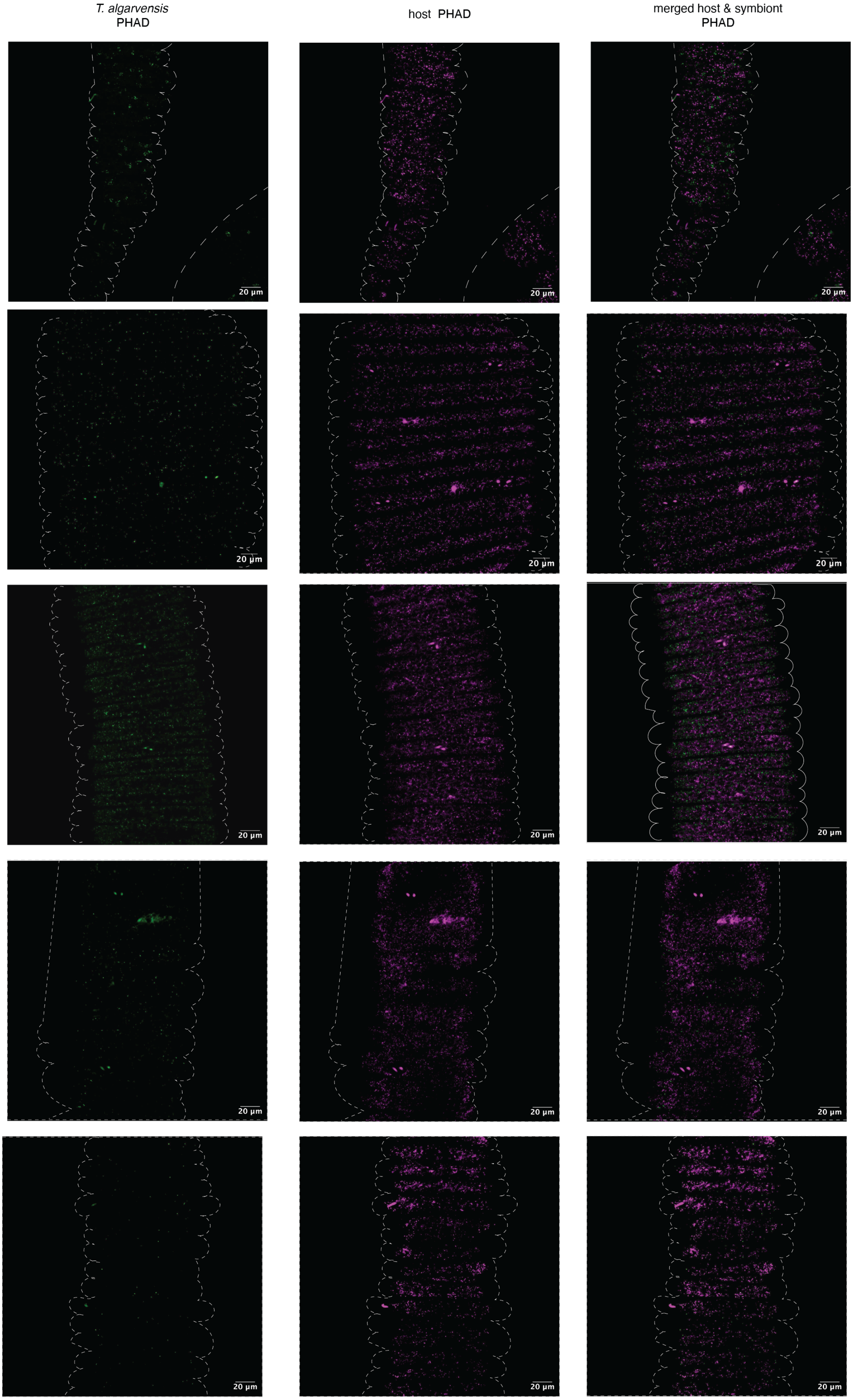
T. algarvensis
PHAD
host PHAD
merged host & symbiont
PHAD
20 µm

Supplementary Figure 5 | The PHAD of *O. algarvensis* is expressed in its epidermal cells. Replicates of whole-mount HCR-FISH of the PHAD mRNA from the host and symbiont. The probes specific to PHAD transcripts of the symbiont *T. algarvensis* (green) and the host *O. algarvensis* (pink) are distinct from each other and are expressed throughout the worm's epidermis. (n = 6 biological replicates)

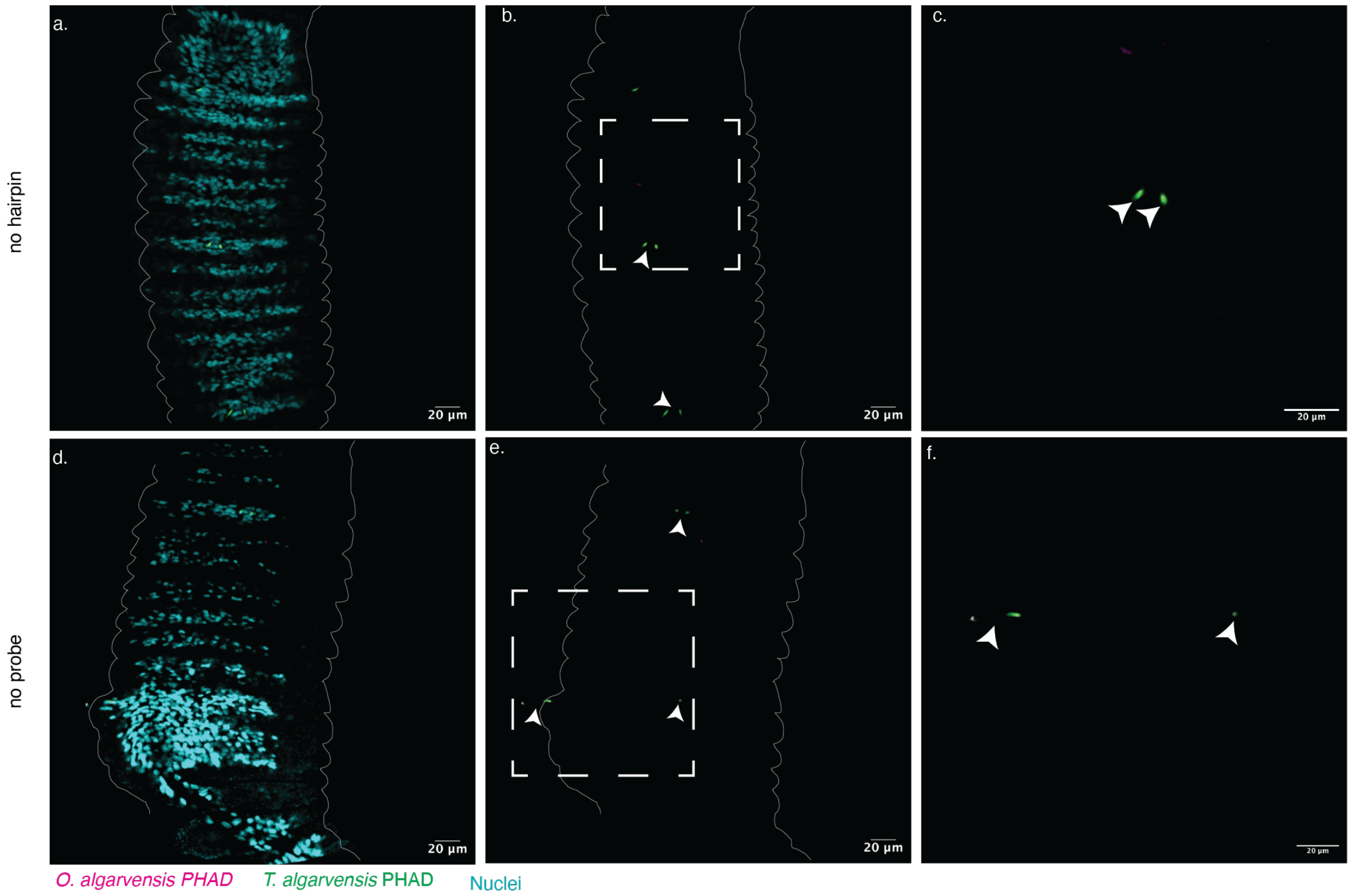

Supplementary Figure 6 | HCR-FISH specificity controls. Whole mount HCR-FISH images of control samples without hairpin amplification reagents (**a,b,c**) or without probes **(d**,**e,f)** demonstrate that the HCR-FISH signals in Figure 3 are specific to their target mRNA transcripts (n = 1). The white triangles in **c,b,e** and **f** indicate the autofluorescence of the worm's setae. The worm cuticle is outlined in white. Cyan = nuclei, magenta = *O. algarvensis* PHAD, green = *T. algarvensis* PHAD

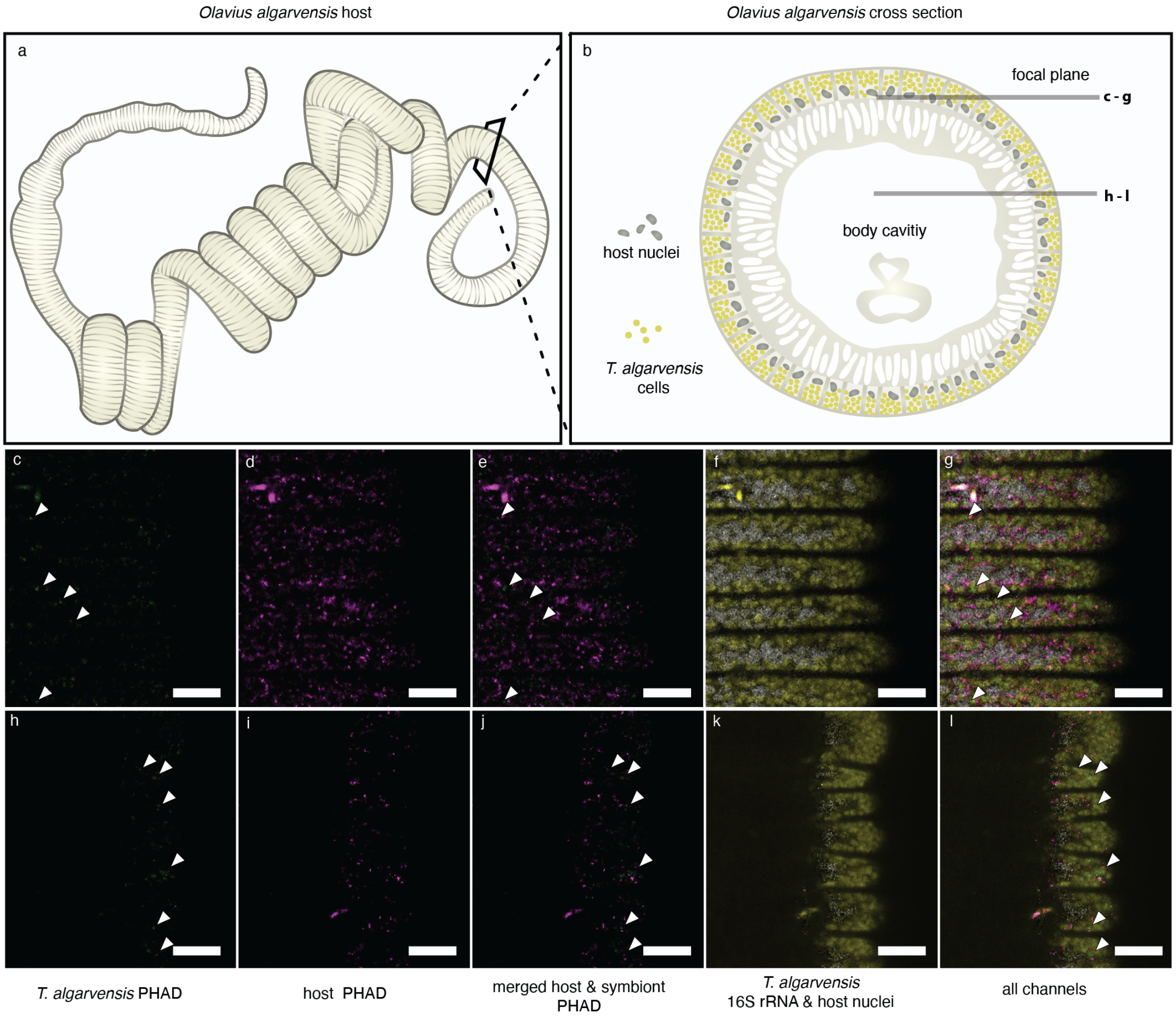

Supplementary Figure 7 | Localization of PHAD transcripts in *O. algarvensis* and its symbiont *T. algarvensis* using whole-mount HCR-FISH. **a,** Schematic drawing of the gutless oligochaete *O. algarvensis,* highlighting the region shown in **(b)**. **b**, Overview of focal planes captured in the confocal images presented in **Figure 3** and panels **c-l**. Symbionts (yellow) are located beneath the cuticle and above the host epidermal cells (gray). **c-g,** The upper focal plane captures the symbiont-rich region directly below the cuticle. **h-l,** The lower focal plane includes the symbiont region located on the right side of the images and the host coelom where no signals were observed. In **c - l**, host PHAD transcripts are shown in pink, symbiont PHAD in green, symbiont 16S rRNA in yellow, and host nuclei in grey. In the merged images (**e** and **j**), host and symbiont PHADs do not overlap, and no PHAD signals are visible in the coelom (n = 6 biological replicates).

a.

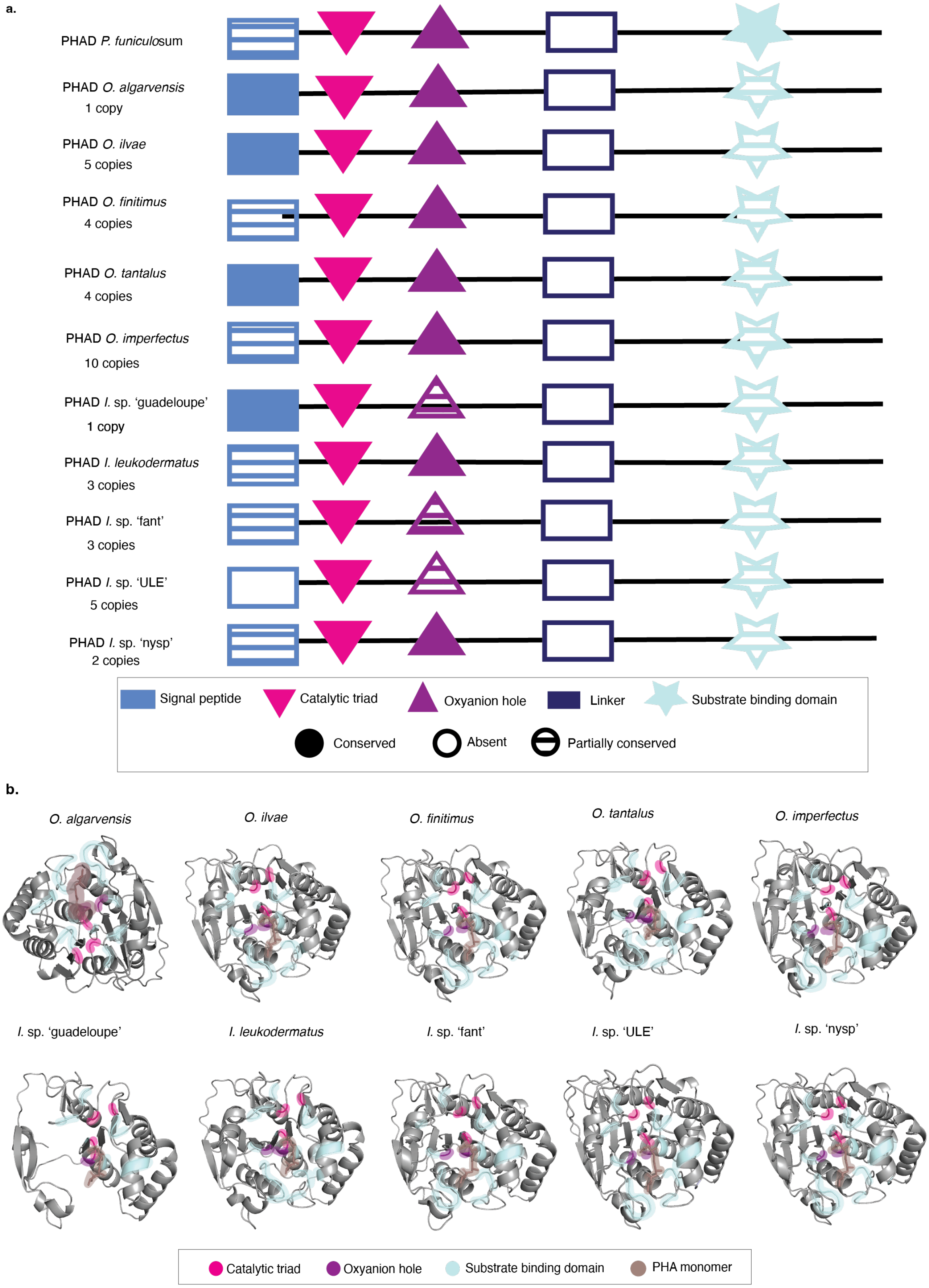
PHAD P. funiculosum
PHAD O. algarvensis
1 copy
PHAD O. ilvae
5 copies
PHAD O. finitimus
4 copies
PHAD O. tantalus
4 copies
PHAD O. imperfectus
10 copies
PHAD I. sp. 'guadeloupe'
1 copy
PHAD I. leukodermatus
3 copies
PHAD I. sp. 'fant'
3 copies
PHAD I. sp. 'ULE'
5 copies
PHAD I. sp. 'nysp'
2 copies
Signal peptide
Catalytic triad
Oxyanion hole
Linker
Substrate binding domain
Conserved
Absent
Partially conserved
b.
O. algarvensis
O. ilvae
O. finitimus
O. tantalus
O. imperfectus
I. sp. 'guadeloupe'
I. leukodermatus
I. sp. 'fant'
I. sp. 'ULE'
I. sp. 'nysp'
Catalytic triad
Oxyanion hole
Substrate binding domain
PHA monomer

Supplementary Figure 8 | Predicted PHAD enzymes from ten gutless oligochaete species. **a,** The *phaZ* genes of the 10 gutless oligochaetes species included in this study encode proteins ranging from 211 - 367 amino acids in length. MAFFT alignments to the experimentally validated fungal PHAD from *P. funiculsoum* (PDB: 2d81) show complete conservation of the catalytic triad and conservation of 70% of the oxyanion hole residues across all predicted PHAD sequences (pink and purple triangles). Nineteen of the 40 predicted PHADs have a signal peptide (blue squares; Supplementary Table 2). Across the full-length alignment, gutless oligochaete PHADs share 14.5% - 33.8% sequence identity and 65.9% - 95.3% sequence coverage relative to the *P. funiculsoum* PHAD. Symbols represent different regions of the protein that are either conserved (filled), partially conserved (stripped) or absent (open). **b**, Structural comparison of AlphaFold2 models from gutless oligochaete PHADs to the crystal structure of *P. funiculosum* PHAD (PDB: 2d81; RMSD = 0.720 - 2.740 Å). The catalytic triad (pink), oxyanion hole (purple) and the substrate-binding site including the residue that holds the polymer in place for degradation (cyan) show strong overlap between the fungal homolog and all predicted gutless oligochaete PHADs. A HB monomer (brown) is predicted to fit within the catalytic pocket of each enzyme (pLDDT = 85 – 96).

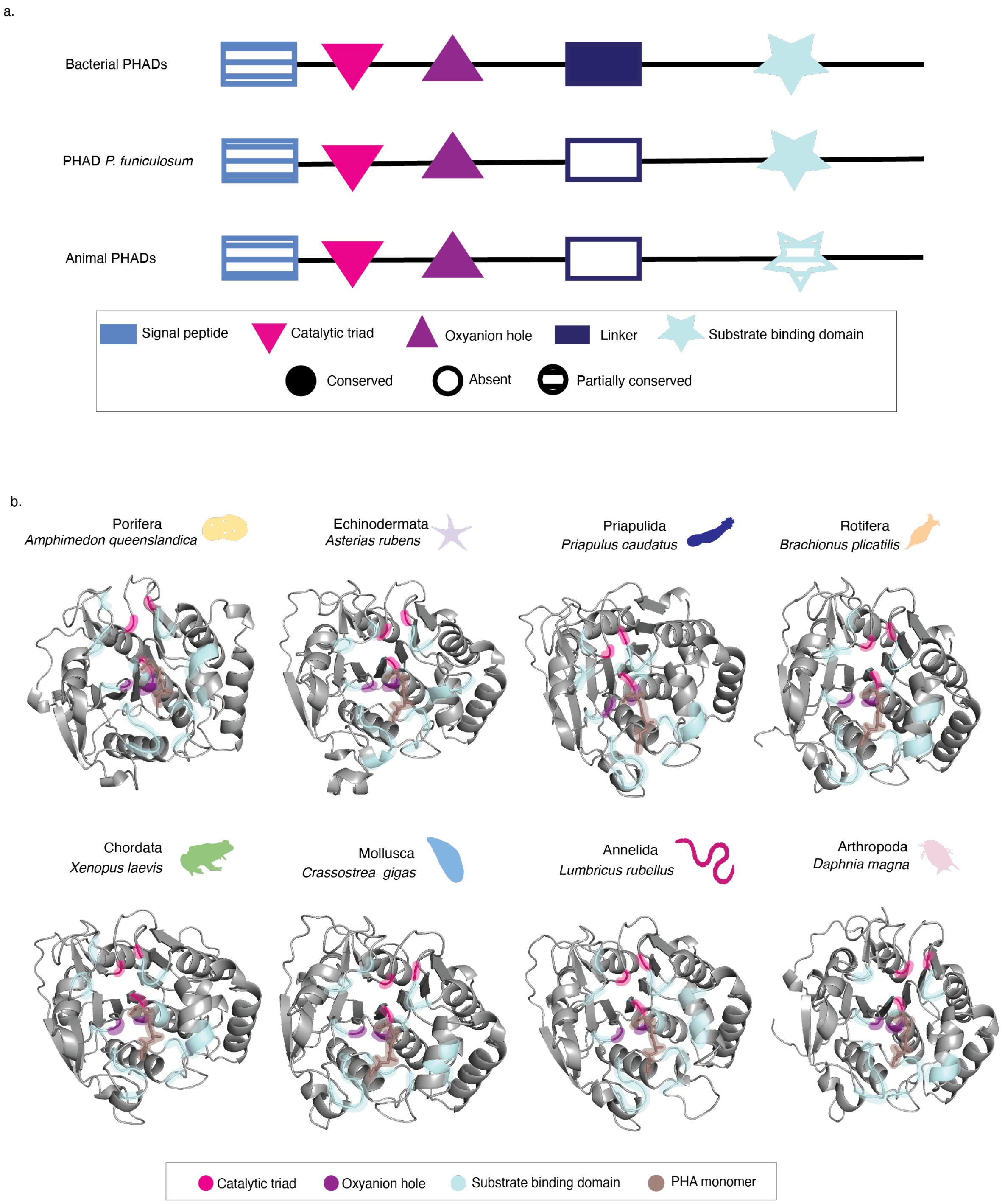
a.
Bacterial PHADs
PHAD P. funiculosum
Animal PHADs
Signal peptide
Catalytic triad
Oxyanion hole
Linker
Substrate binding domain
Conserved
Absent
Partially conserved
b.
Porifera
Amphimedon queenslandica
Echinodermata
Asterias rubens
Priapulida
Priapulus caudatus
Rotifera
Brachionus plicatilis
Chordata
Xenopus laevis
Mollusca
Crassostrea gigas
Annelida
Lumbricus rubellus
Arthropoda
Daphnia magna
Catalytic triad
Oxyanion hole
Substrate binding domain
PHA monomer

Supplementary Figure 9 | Metazoan PHADs share a conserved domain architecture with a fungal homolog. a, Schematic overview of the primary structure of animal PHADs compared with the fungal homolog from *P. funiculosum* (PDB: 2d81). Primary structure analyses indicate that metazoan PHADs have a higher similarity to the fungal PHAD of *P. funiculsoum* (PDB: 2d81) than to bacterial PHADs. Animal PHADs encode proteins ranging from 190 - 1166 amino acids in length (Supplementary Table 2). MAFFT alignments revealed that metazoan PHADs share between of 17.9% - 36.3% sequence identity with the fungal PHAD. The catalytic site (pink triangles) and oxyanion hole (purple triangles) are fully conserved across all metazoan PHADs, and 75% of the enzymes have a signal peptide (blue square; Supplementary Table 2). Symbols indicate protein regions that are conserved (filled), partially conserved (stripped) or absent (open). **b,** Structural comparison of AlphaFold2-predicted metazoan PHADs aligned to the crystal structure of the *P. funiculsoum* PHAD (RMSD 0.668 to 0.904 Å). The catalytic triad (pink), oxyanion hole (purple), polymer-stabilizing residue (cyan) and substrate-binding region (cyan) show strong overlap. A HB monomer (brown) is predicted to fit within the catalytic pocket of each enzyme (pLDDT = 87 – 97).

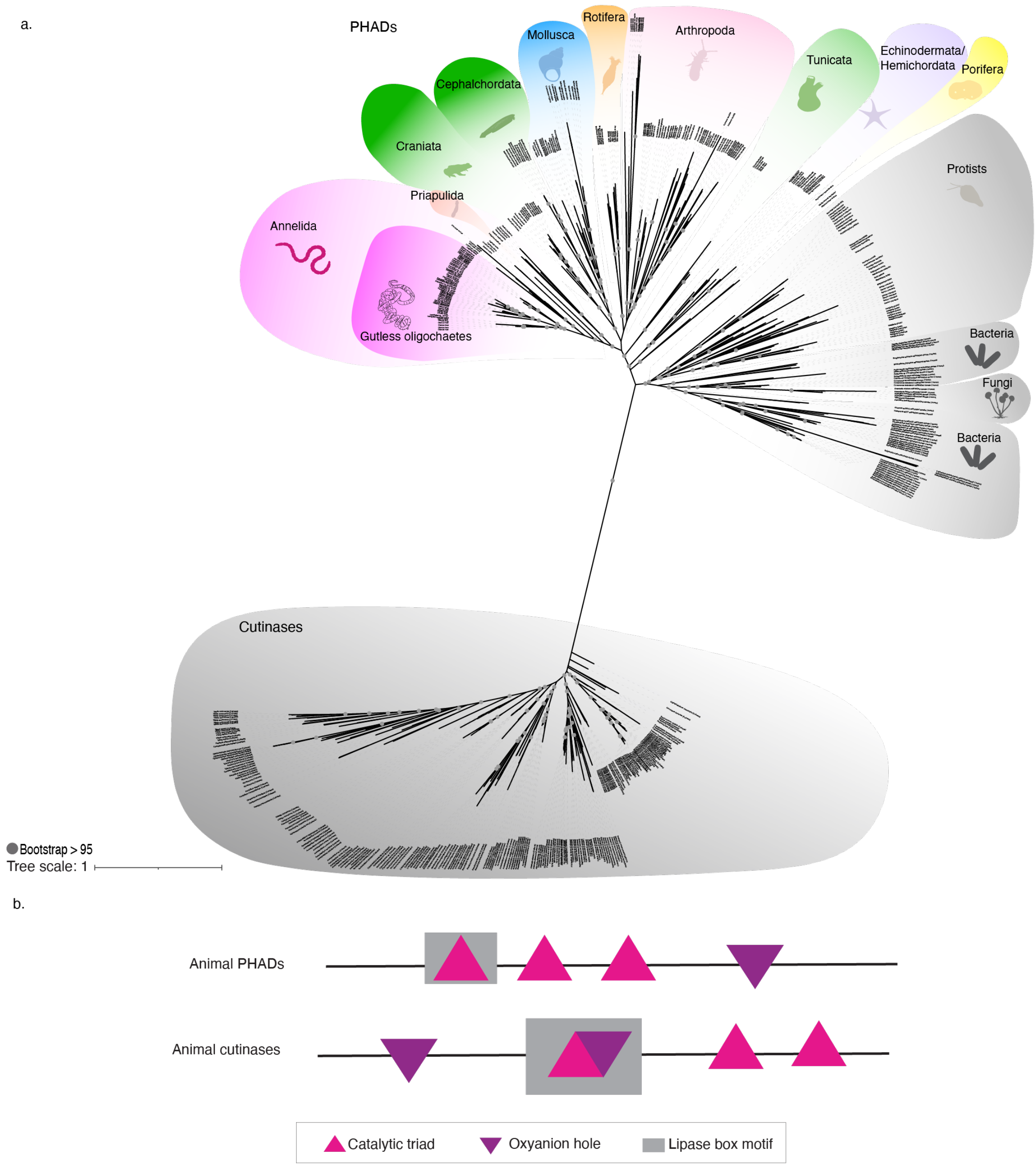

Supplementary Figure 10 | Phylogenetic analyses distinguish animal PHADs from cutinases. a. Phylogenetic analysis shows that animal PHADs cluster with bacterial and fungal PHADs, whereas animal cutinases form a distinct and separate clade. Animal cutinase sequences were retrieved from the ESTHER database[38] and aligned together with fungal, bacterial, protist, and animal PHADs. An unrooted maximum likelihood tree was calculated from the alignment using IQ-TREE with ultrafast bootstrap support (1,000 replicates). PHADs, including those from animals (colored clades), cluster separately from the cutinase clade. **b**, Schematic representation of the primary structure alignment of animal PHADs and cutinases. The catalytic triad of cutinases and PHADs shows no conservation between cutinases and PHADs. In addition, the oxyanion hole in cutinases consists of two residues located at the N terminus and within the lipase box, whereas in animal PHADs the oxyanion hole is positioned behind the lipase box motif.

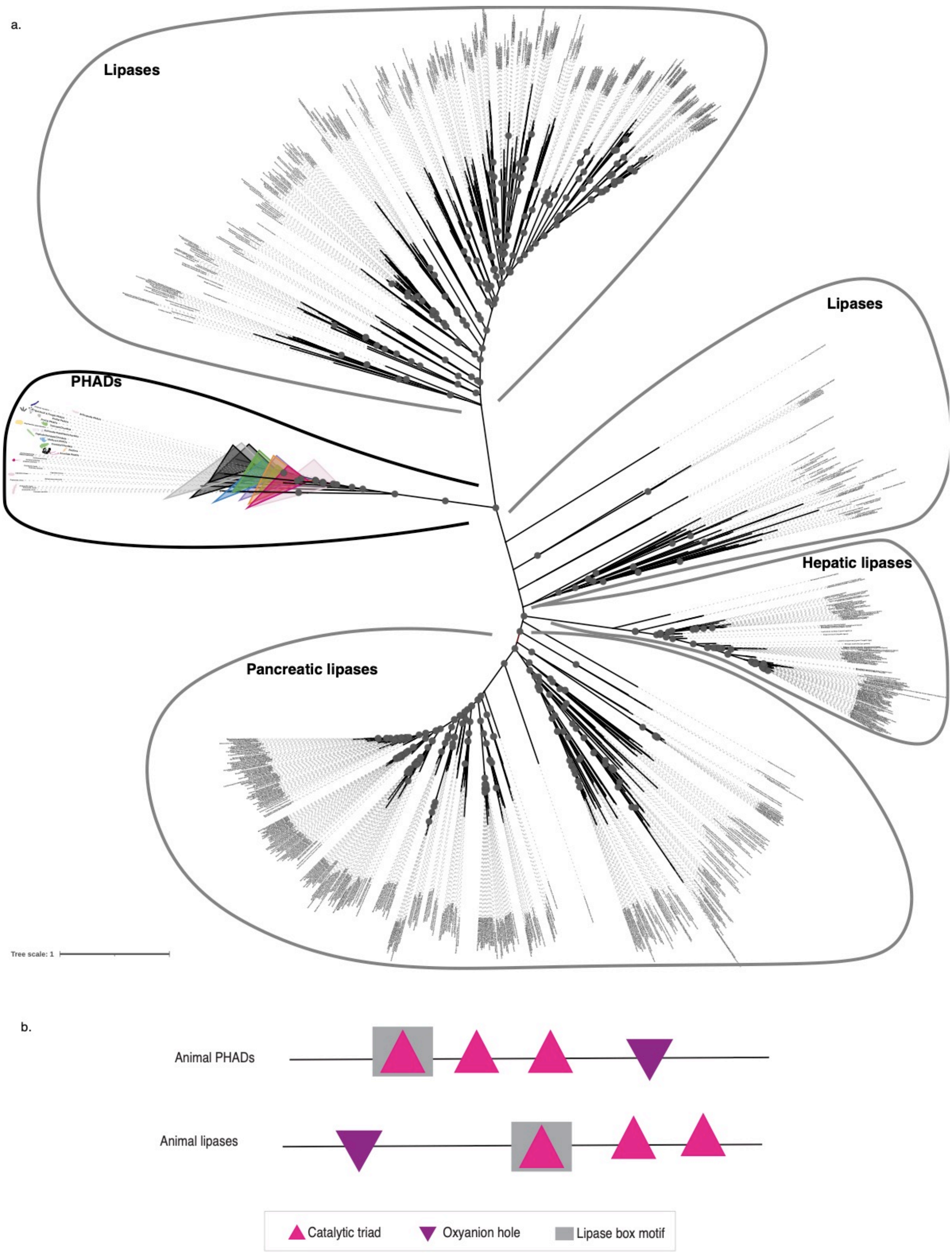
a.
Lipases
PHADs
Lipases
Hepatic lipases
Pancreatic lipases
Tree scale: 1
b.
Animal PHADs
Animal lipases
Catalytic triad
Oxyanion hole
Lipase box motif

Supplementary Figure 11 | Phylogenetic analyses distinguish animal PHADs from lipases. a, Phylogenetic analysis shows that all animal PHADs cluster with bacterial and fungal PHADs, whereas lipases form distinct and separate clades. Animal lipase sequences were retrieved from the ESTHER database[38] and aligned together with fungal, bacterial, protist, and animal PHADs. An unrooted maximum likelihood tree was calculated from the alignment using IQ-TREE with ultrafast bootstrap support (1,000 replicates). Lipases group into four distinct clades, all clearly separated from PHADs, including animal PHADs (colored clades). **b,** Schematic representation of the primary structure alignment of animal PHADs and lipases. The catalytic triad shows no conservation between lipases and PHADs. In addition, the asparagine residue of the catalytic triad in PHADs is replaced by a glutamic acid residue in lipases, providing a clear structural distinction between the two enzyme classes.

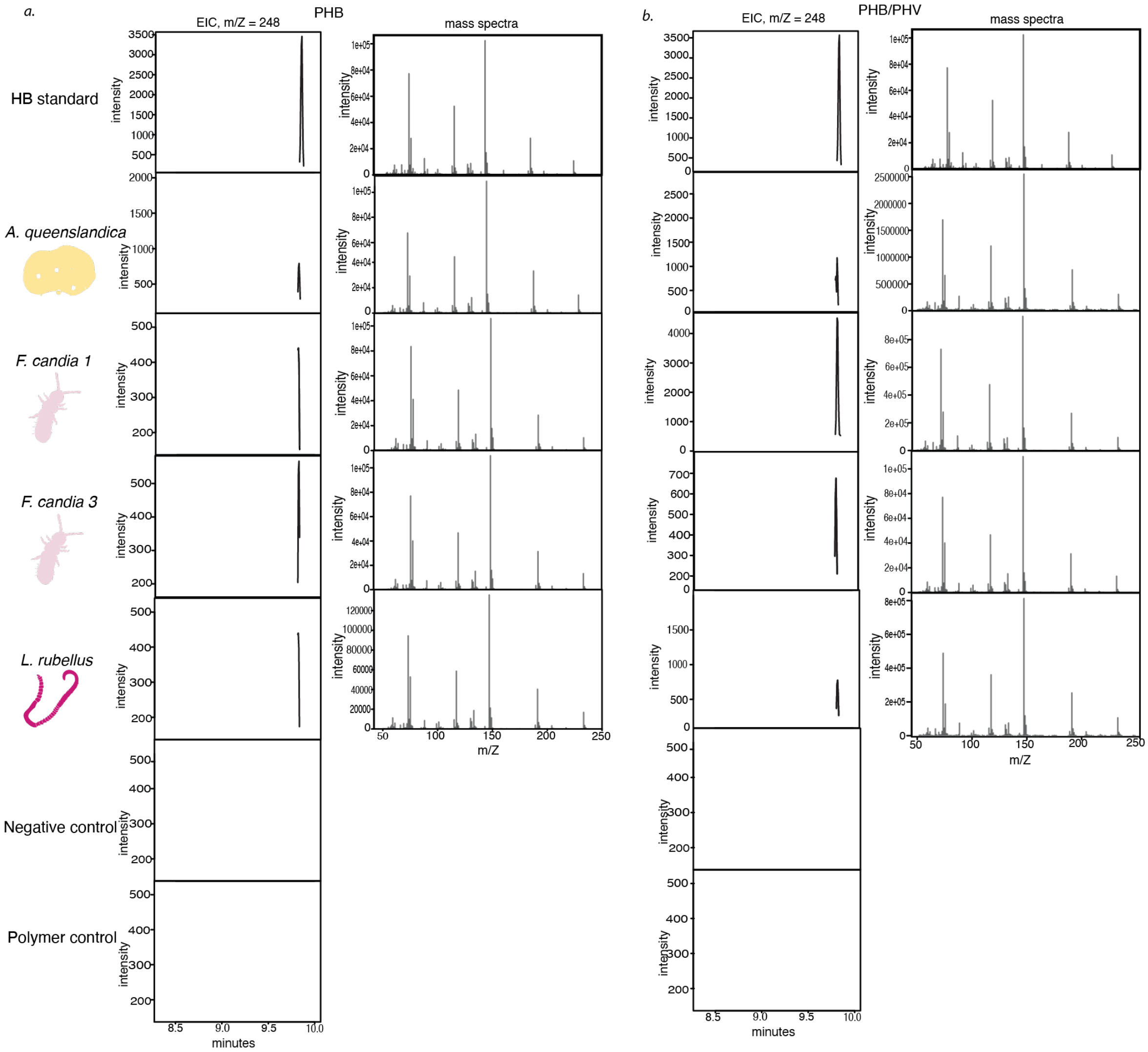

Supplementary Figure 12 | PHADs from terrestrial and marine animals degrade PHB and PHB/PHV into HB monomers. a,b, Chromatograms and corresponding mass spectra of the HB monomer peak detected in PHAD assays of the sponge *A. queenslandica*, the springtail *F. candia* (which has two PHAD homologs) and the earthworm *L. rubellus* using (**a**) PHB and (**b**) PHB/PHV. All detected HB monomers match the retention time (9.56 min) and mass spectra of the HB standard. No HB monomers were detected in assays conducted using a cell free expression with a non-sense vector (negative control) or the no-enzyme control (PHB or PHB/PHV without PHAD).

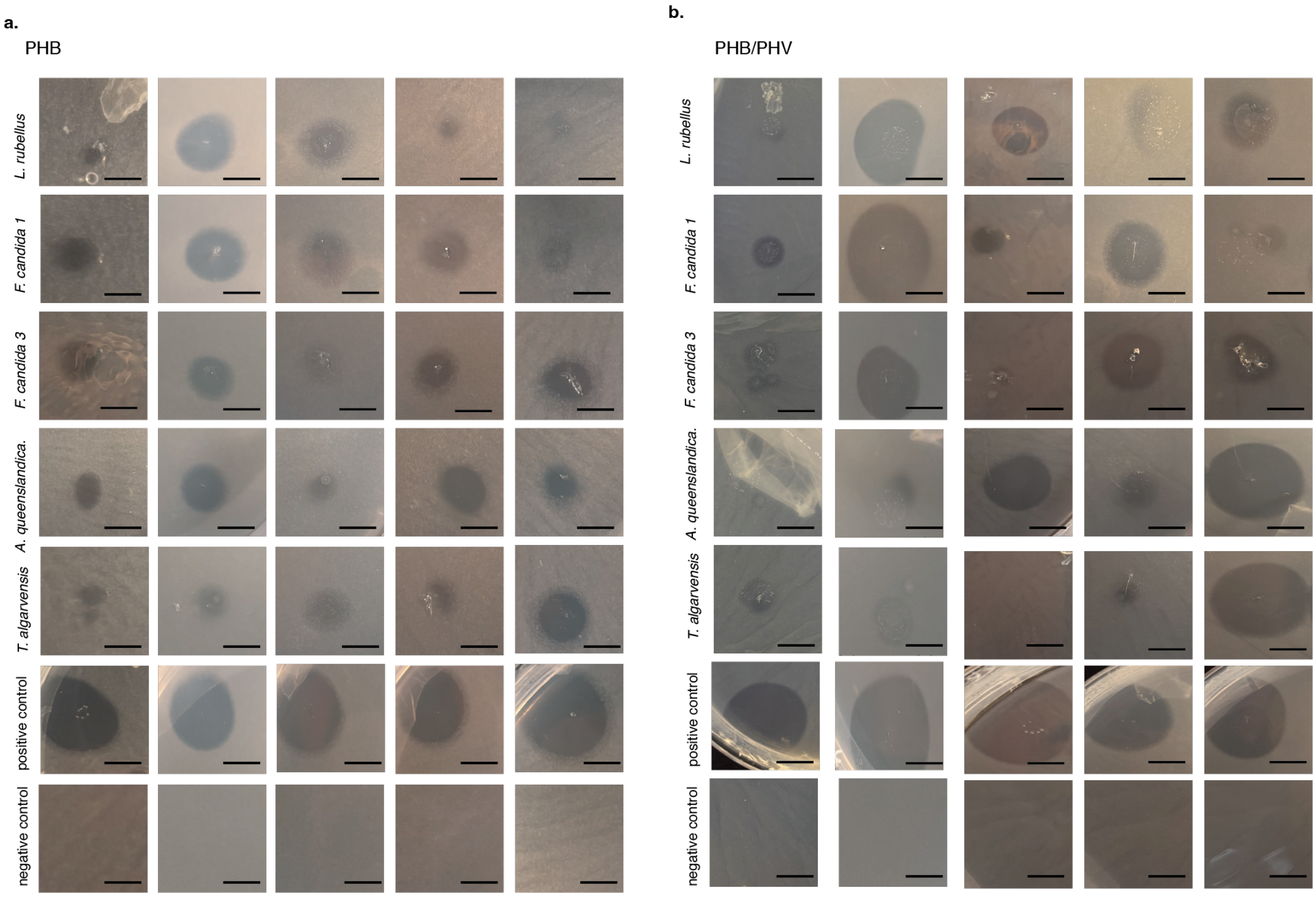

Supplementary Figure 13 | Animal PHADs degrade PHB and PHB/PHV in spot assays. Enzyme spot assays (n = 5 biological replicates of one heterologous expression) of four animal PHADs and the symbiont *T. algarvensis* PHAD on **a**, PHB and **b,** PHB/PHV assay plates. Expression of a nonsense vector produces no clearance zone and thus indicates no enzymatic activity (negative control).

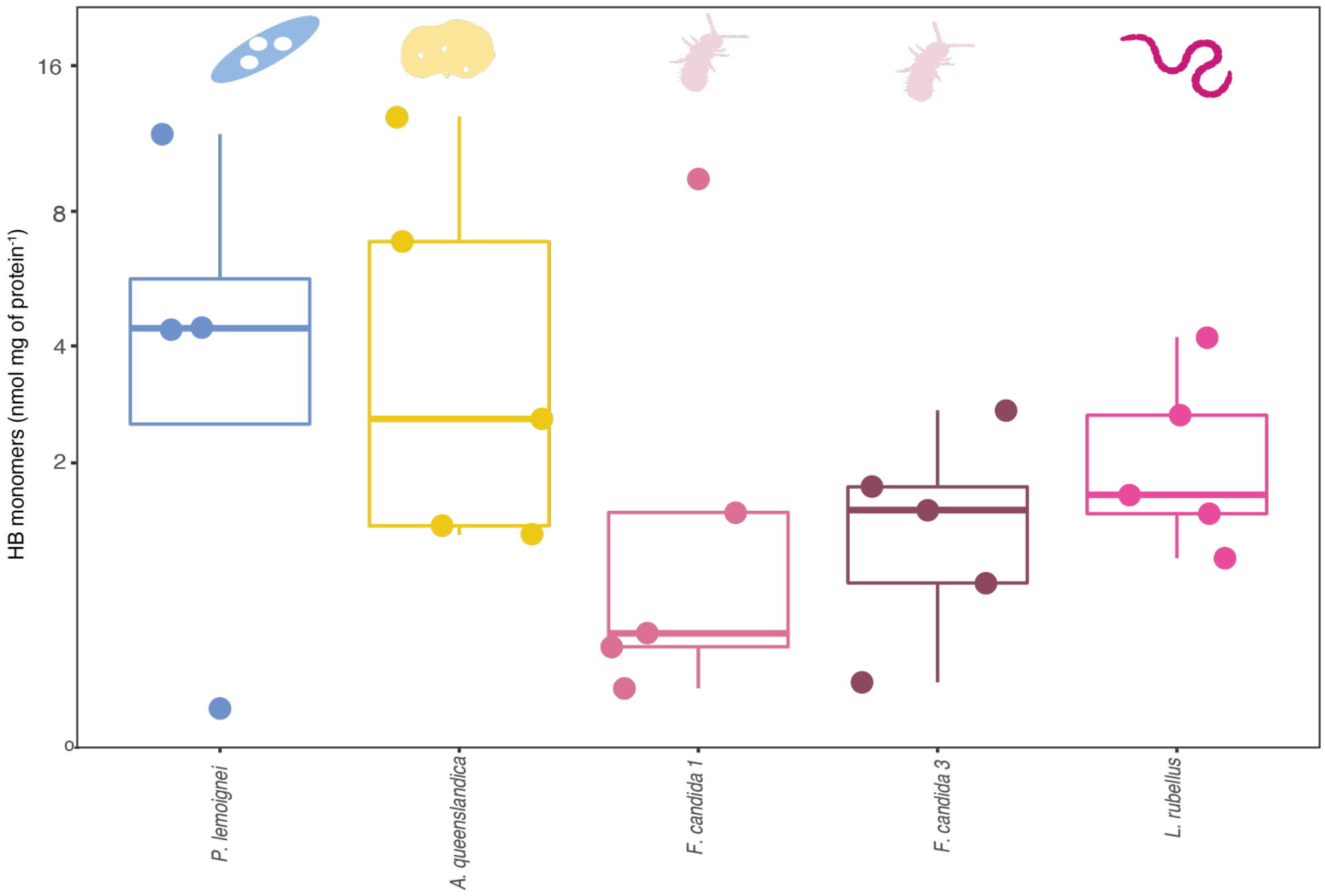

Supplementary Figure 14 | Animal PHADs degrade sPHA into its monomeric units. Boxplots show concentrations of HB monomers, measured by GC-MS, produced in PHAD enzyme assays using PHB/PHV. All tested enzymes generate measurable amounts of HB monomers, including PHADs from the sponge *A. queenslandica* (yellow; n = 5 biological replicates of one heterologous expression; upper whisker = 12.6 nmol/mg protein, lower whisker = 1.2 nmol/mg protein; Q1 = 1.2 nmol/mg protein; Q3 = 6.9 nmol/mg protein; Median = 2.6 nmol/mg protein), the springtail *F. candida* (light pink, two homologs; n = 5 biological replicates of one heterologous expression; *F. canida* 1: upper whisker = 1.4 nmol/mg protein, lower whisker = 0.1 nmol/mg protein; Q1 = 0.3 nmol/mg protein; Q3 = 1.4 nmol/mg protein; Median = 0.4 nmol/mg protein, *F. candida* 3: upper whisker = 2.8 nmol/mg protein, lower whisker = 0.2 nmol/mg protein; Q1 = 0.8 nmol/mg protein; Q3 = 1.7 nmol/mg protein; Median = 1.4 nmol/mg protein), and the earthworm *L. rubellus* (pink; n = 5 biological replicates of one heterologous expression; upper whisker = 4.2 nmol/mg protein, lower whisker = 0.9 nmol/mg protein; Q1 = 1.4 nmol/mg protein; Q3 = 2.7 nmol/mg protein; Median = 1.6 nmol/mg protein). No HB monomers were detected using a nonsense vector (negative control) or no-enzyme control (see Supplementary Figure 12 & 13). Hydroxyvalerate (HV) monomers were not detected because the PHB/PHV substrate contained only 3% HV, which is below the detection limit of the assay. As a positive control we used the PHAD of P. *lemoignei* (blue; n = 4 biological replicates of one heterologous expression; upper whisker = 11.6 nmol/mg protein, lower whisker = 0.02 nmol/mg protein; Q1 = 2.2 nmol/mg protein; Q3 = 8.0 nmol/mg protein; Median = 4.4 nmol/mg protein).

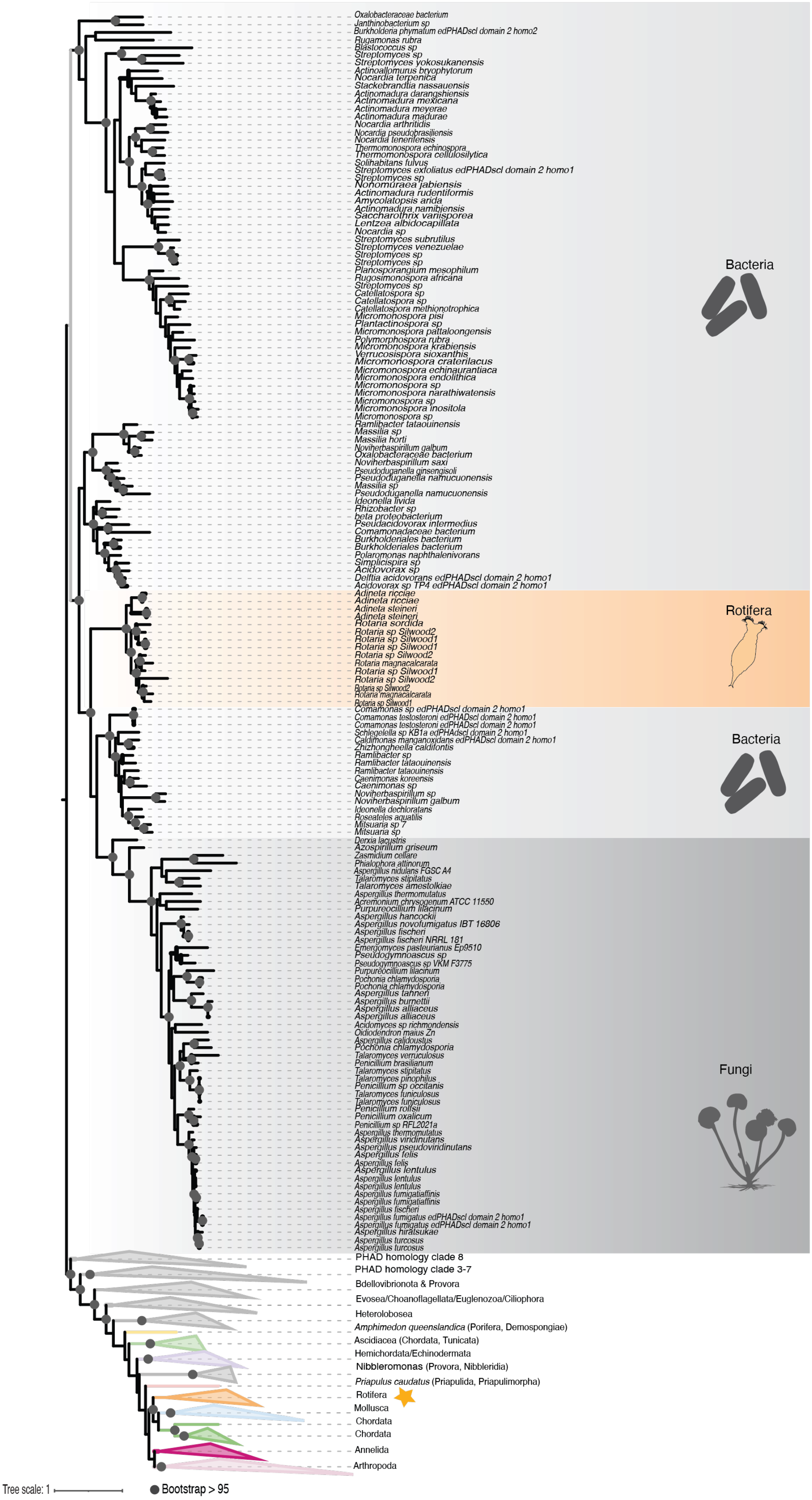

Oxalobacteraceae bacterium
Janthinobacterium sp
Burkholderia phymatum edPHADscl domain 2 homo2
Rugamonas rubra
Blastococcus sp
Streptomyces sp
Streptomyces yokosukanensis
Actinoallomurus bryophytorum
Nocardia terpenica
Stackebrandtia nassauensis
Actinomadura darangshiensis
Actinomadura mexicana
Actinomadura meyerae
Actinomadura madurae
Nocardia arthritidis
Nocardia pseudobrasiliensis
Nocardia tenerifensis
Thermomonospora echinospora
Thermomonospora cellulosilytica
Solihabitans fulvus
Streptomyces exfoliatus edPHADscl domain 2 homo1
Streptomyces sp
Nonomuraea jabiensis
Actinomadura rudentiformis
Amycolatopsis arida
Actinomadura namibiensis
Saccharothrix variisporea
Lentzea albidocapillata
Nocardia sp
Streptomyces subrutilus
Streptomyces venezuelae
Streptomyces sp
Streptomyces sp
Planosporangium mesophilum
Rugosimonospora africana
Streptomyces sp
Catellatospora sp
Catellatospora sp
Catellatospora methionotrophica
Micromonospora pisi
Plantactinospora sp
Micromonospora pattaloongensis
Polymorphospora rubra
Micromonospora krabiensis
Verrucosispora sioxanthis
Micromonospora craterilacus
Micromonospora echinaurantiaca
Micromonospora endolithica
Micromonospora sp
Micromonospora narathiwatensis
Micromonospora sp
Micromonospora inositola
Micromonospora sp
Ramlibacter tataouinensis
Massilia sp
Massilia horti
Noviherbaspirillum galbum
Oxalobacteraceae bacterium
Noviherbaspirillum saxi
Pseudoduganella ginsengisoli
Pseudoduganella namucuonensis
Massilia sp
Pseudoduganella namucuonensis
Ideonella livida
Rhizobacter sp
beta proteobacterium
Pseudacidovorax intermedius
Comamonadaceae bacterium
Burkholderiales bacterium
Burkholderiales bacterium
Polaromonas naphthalenivorans
Simplicispira sp
Acidovorax sp
Delftia acidovorans edPHADscl domain 2 homo1
Acidovorax sp TP4 edPHADscl domain 2 homo1
Adineta ricciae
Adineta ricciae
Adineta steineri
Adineta steineri
Rotaria sordida
Rotaria sp Silwood2
Rotaria sp Silwood1
Rotaria sp Silwood1
Rotaria sp Silwood2
Rotaria magnacalcarata
Rotaria sp Silwood1
Rotaria sp Silwood2
Rotaria sp Silwood2
Rotaria magnacalcarata
Rotaria sp Silwood1
Comamonas sp edPHADscl domain 2 homo1
Comamonas testosteroni edPHADscl domain 2 homo1
Comamonas testosteroni edPHADscl domain 2 homo1
Schlegelella sp KB1a edPHAdscl domain 2 homo1
Caldimonas manganoxidans edPHADscl domain 2 homo1
Zhizhongheella caldifontis
Ramlibacter sp
Ramlibacter tataouinensis
Ramlibacter tataouinensis
Caenimonas koreensis
Caenimonas sp
Noviherbaspirillum sp
Noviherbaspirillum galbum
Ideonella dechloratans
Roseateles aquatilis
Mitsuaria sp 7
Mitsuaria sp
Derxia lacustris
Azospirillum griseum
Zasmidium cellare
Phialophora attinorum
Aspergillus nidulans FGSC A4
Talaromyces stipitatus
Talaromyces amestolkiae
Aspergillus thermomutatus
Acremonium chrysogenum ATCC 11550
Purpureocillium lilacinum
Aspergillus hancockii
Aspergillus novofumigatus IBT 16806
Aspergillus fischeri
Aspergillus fischeri NRRL 181
Emergomyces pasteurianus Ep9510
Pseudogymnoascus sp
Pseudogymnoascus sp VKM F3775
Purpureocillium lilacinum
Pochonia chlamydosporia
Pochonia chlamydosporia
Aspergillus tanneri
Aspergillus burnettii
Aspergillus alliaceus
Aspergillus alliaceus
Acidomyces sp richmondensis
Oidiodendron maius Zn
Aspergillus calidoustus
Pochonia chlamydosporia
Talaromyces verruculosus
Penicillium brasilianum
Talaromyces stipitatus
Talaromyces pinophilus
Penicillium sp occitanis
Talaromyces funiculosus
Talaromyces funiculosus
Penicillium rolfsii
Penicillium oxalicum
Penicillium sp RFL2021a
Aspergillus thermomutatus
Aspergillus viridinutans
Aspergillus pseudoviridinutans
Aspergillus felis
Aspergillus felis
Aspergillus lentulus
Aspergillus lentulus
Aspergillus lentulus
Aspergillus fumigatiaffinis
Aspergillus fumigatiaffinis
Aspergillus fischeri
Aspergillus fumigatus edPHADscl domain 2 homo1
Aspergillus fumigatus edPHADscl domain 2 homo1
Aspergillus hiratsukae
Aspergillus turcosus
Aspergillus turcosus
PHAD homology clade 8
PHAD homology clade 3-7
Bdellovibrionota & Provora
Evosea/Choanoflagellata/Euglenozoa/Ciliophora
Heterolobosea
Amphimedon queenslandica (Porifera, Demospongiae)
Ascidiacea (Chordata, Tunicata)
Hemichordata/Echinodermata
Nibbleromonas (Provora, Nibbleridia)
Priapulus caudatus (Priapulida, Priapulimorpha)
Rotifera
Mollusca
Chordata
Chordata
Annelida
Arthropoda
Bacteria
Rotifera
Bacteria
Fungi
Tree scale: 1
Bootstrap > 95

Supplementary Figure 15 | Phylogenetic clustering of rotifer and bacterial Burkholderiales PHADs suggests a recent horizontal gene transfer (HGT) event. A maximum likelihood phylogenetic tree inferred using IQ TREE with ultrafast bootstrap (1000 replicates) shows that rotifer PHADs formed two distinct groups. One group clusters within the animal PHAD clade (orange star). A second group (highlighted in orange) clusters with PHADs from Burkholderiales, which form a sister group to fungal PHADs. This phylogenetic pattern is consistent with the possibility that rotifers acquired one PHAD copy via a recent HGT event from bacteria.

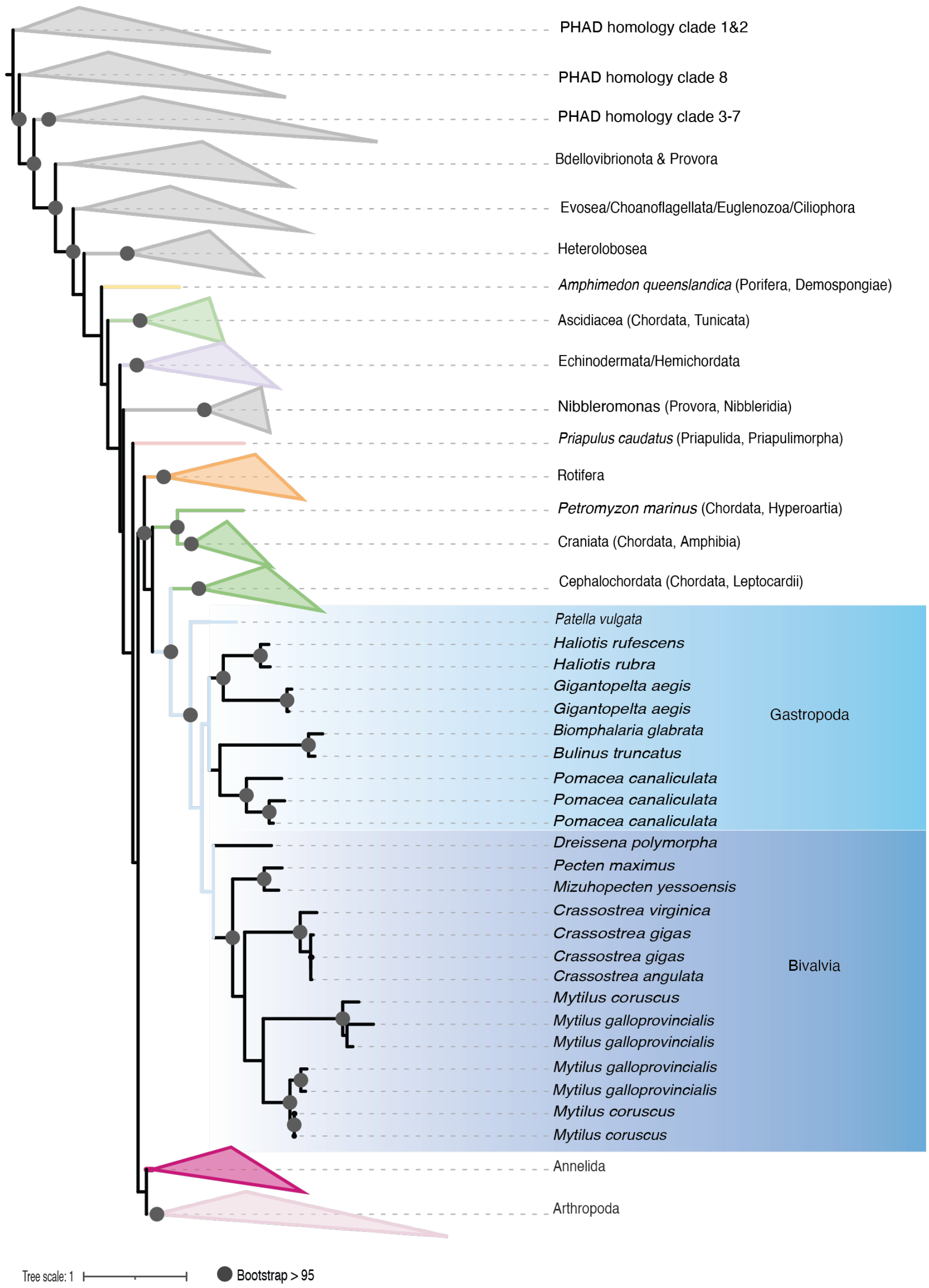

Supplementary Figure 16 | Mollusca PHADs cluster according to class. A maximum likelihood phylogenetic tree inferred using IQ TREE with ultrafast bootstrap (1000 replicates) shows that molluscan PHADs are split into two classes corresponding to the classes Bivalvia and Gastropoda. This separation is consistent with established phylogenetic relationships within the Mollusca.

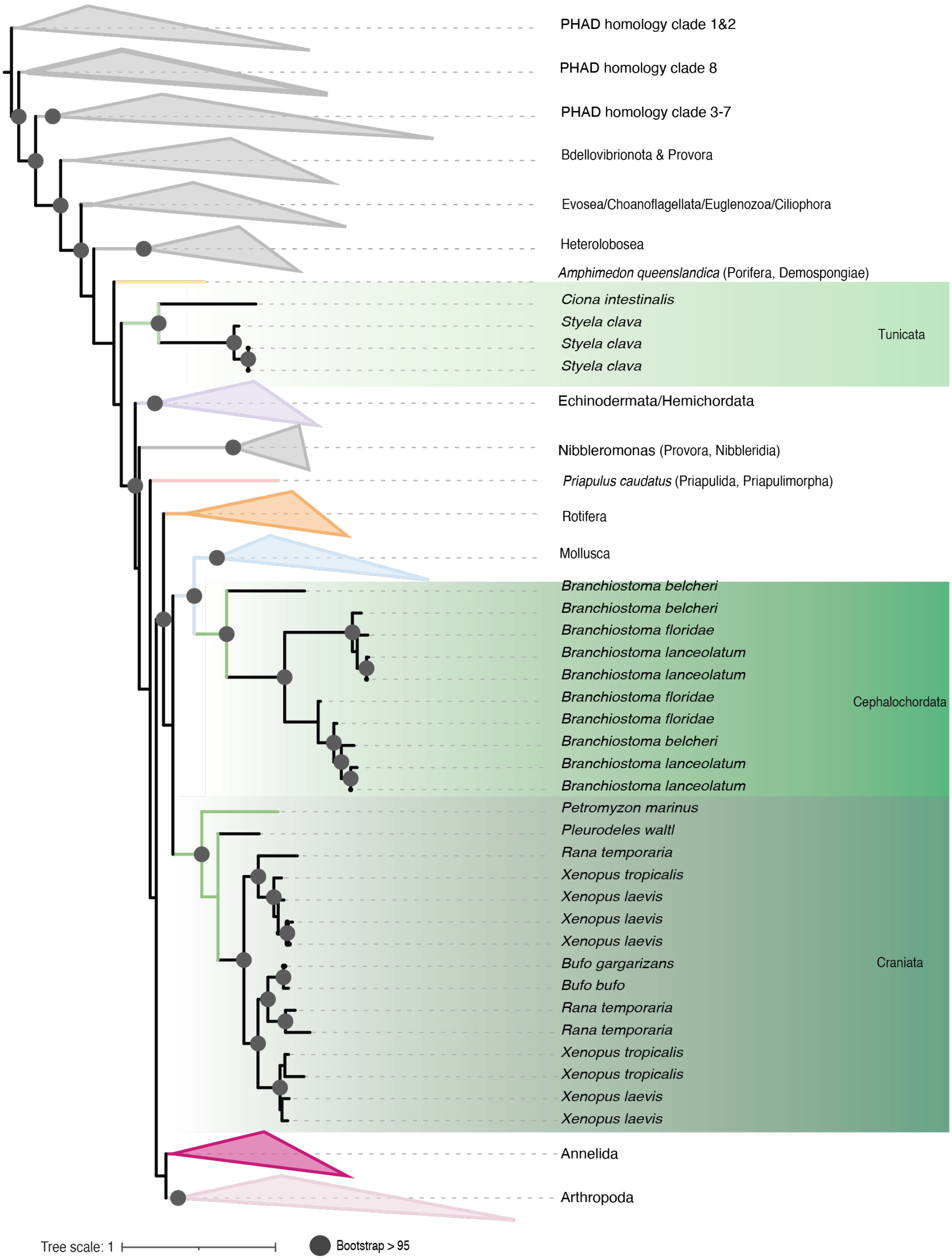

Supplementary Figure 17 | Chordata PHADs cluster according to their subphylum. A maximum likelihood phylogenetic tree inferred using IQ TREE with ultrafast bootstrap support (1000 replicates) shows that chordate PHADs segregate into clades corresponding to the major chordate subphyla Craniata, Cephalochordata, and Tunicata. PHADs from tunicates form a distinct branch separate from those of craniates and cephalochordates, consistent with established phylogenetic relationships within Chordata.

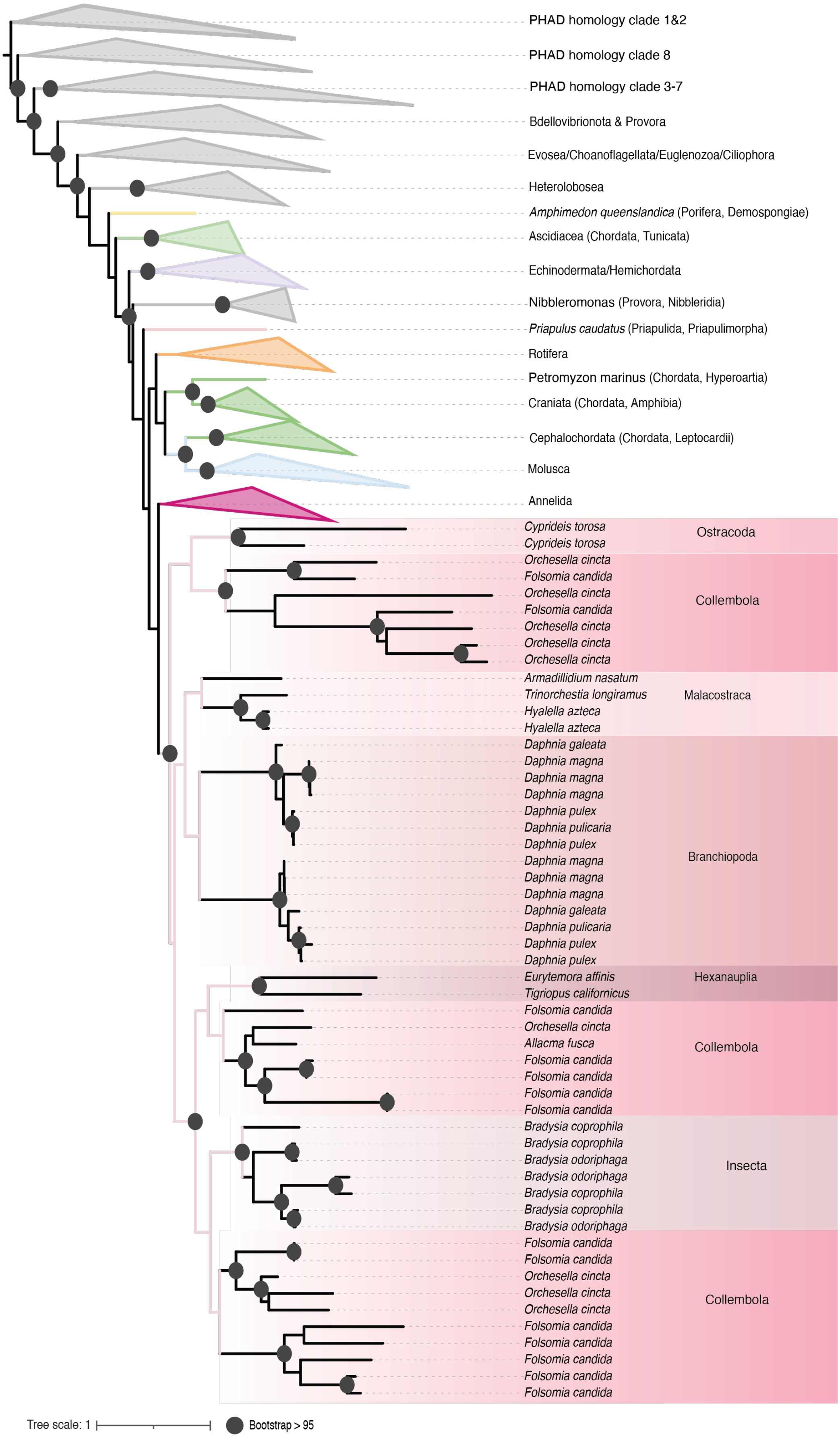

PHAD homology clade 1&2
PHAD homology clade 8
PHAD homology clade 3-7
Bdellovibrionota & Provora
Evosea/Choanoflagellata/Euglenozoa/Ciliophora
Heterolobosea
Amphimedon queenslandica (Porifera, Demospongiae)
Ascidiacea (Chordata, Tunicata)
Echinodermata/Hemichordata
Nibbleromonas (Provora, Nibbleridia)
Priapulus caudatus (Priapulida, Priapulimorpha)
Rotifera
Petromyzon marinus (Chordata, Hyperoartia)
Craniata (Chordata, Amphibia)
Cephalochordata (Chordata, Leptocardii)
Molusca
Annelida
Cyprideis torosa
Cyprideis torosa
Ostracoda
Orchesella cincta
Folsomia candida
Orchesella cincta
Folsomia candida
Orchesella cincta
Orchesella cincta
Orchesella cincta
Collembola
Armadillidium nasatum
Trinorchestia longiramus
Hyalella azteca
Hyalella azteca
Malacostraca
Daphnia galeata
Daphnia magna
Daphnia magna
Daphnia magna
Daphnia pulex
Daphnia pulicaria
Daphnia pulex
Daphnia magna
Daphnia magna
Daphnia magna
Daphnia galeata
Daphnia pulicaria
Daphnia pulex
Daphnia pulex
Branchiopoda
Eurytemora affinis
Tigriopus californicus
Hexanauplia
Folsomia candida
Orchesella cincta
Allacma fusca
Folsomia candida
Folsomia candida
Folsomia candida
Folsomia candida
Collembola
Bradysia coprophila
Bradysia coprophila
Bradysia odoriphaga
Bradysia odoriphaga
Bradysia coprophila
Bradysia coprophila
Bradysia odoriphaga
Insecta
Folsomia candida
Folsomia candida
Orchesella cincta
Orchesella cincta
Orchesella cincta
Folsomia candida
Folsomia candida
Folsomia candida
Folsomia candida
Folsomia candida
Collembola
Tree scale: 1
Bootstrap > 95

Supplementary Figure 18 | Arthropod PHADs do not fully recapitulate phylum-level phylogeny, likely because of multiple PHAD homologs in some Collembola (springtails). A maximum likelihood phylogenetic tree inferred using IQ TREE with ultrafast bootstrap support (1000 replicates) shows that arthropod PHADs form a monophyletic clade. Within this clade, however, PHADs from collembolans segregate into three distinct branches rather than clustering according to established arthropod relationships. This pattern may reflect ancient duplication followed by differential loss in most arthropods, rapid diversification in collembolans, or limited phylogenetic resolution.

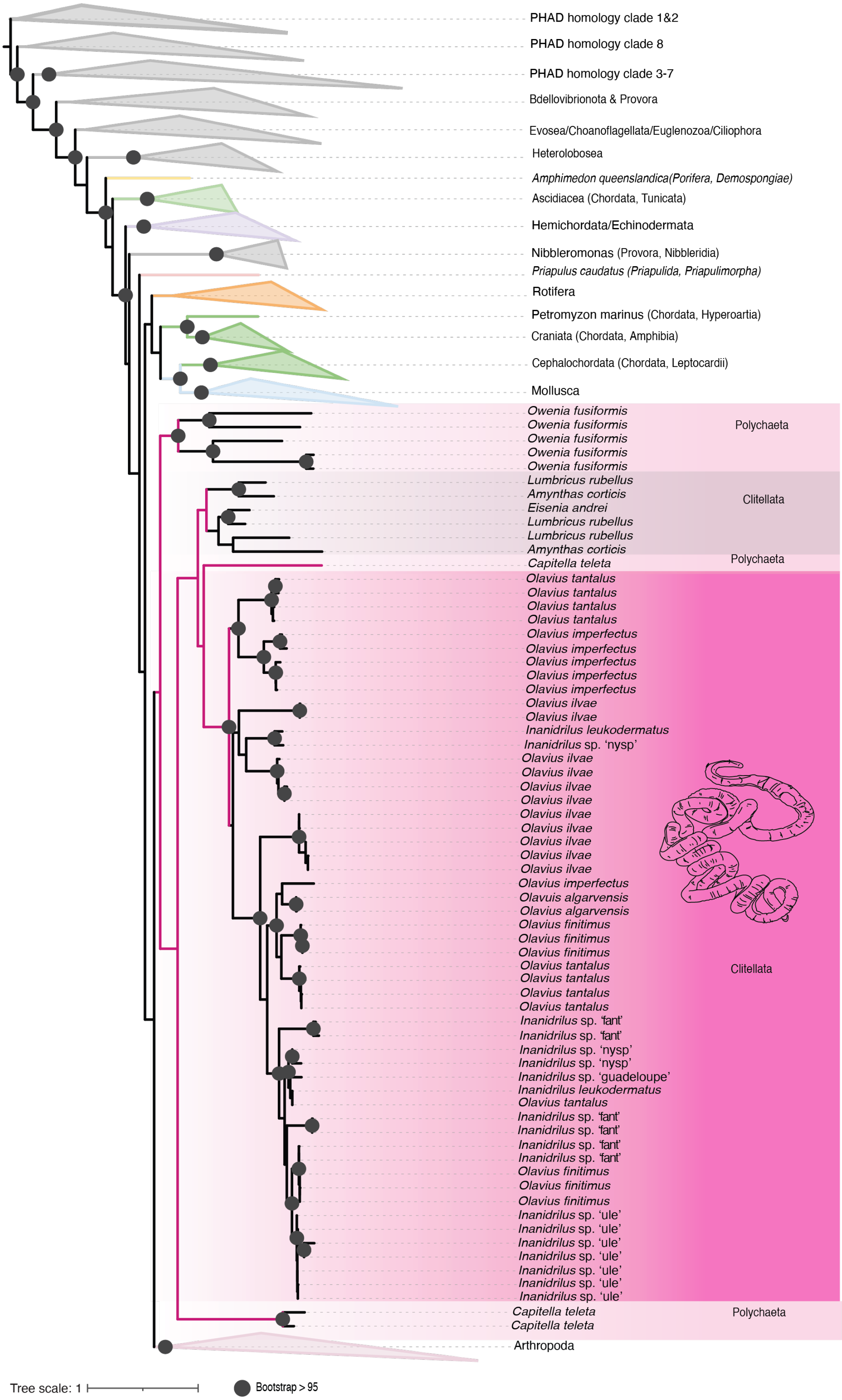
PHAD homology clade 1&2
PHAD homology clade 8
PHAD homology clade 3-7
Bdellovibrionota & Provora
Evosea/Choanoflagellata/Euglenozoa/Ciliophora
Heterolobosea
Amphimedon queenslandica(Porifera, Demospongiae)
Ascidiacea (Chordata, Tunicata)
Hemichordata/Echinodermata
Nibbleromonas (Provora, Nibbleridia)
Priapulus caudatus (Priapulida, Priapulimorpha)
Rotifera
Petromyzon marinus (Chordata, Hyperoartia)
Craniata (Chordata, Amphibia)
Cephalochordata (Chordata, Leptocardii)
Mollusca
Owenia fusiformis
Owenia fusiformis
Owenia fusiformis
Owenia fusiformis
Owenia fusiformis
Polychaeta
Lumbricus rubellus
Amynthas corticis
Eisenia andrei
Lumbricus rubellus
Lumbricus rubellus
Amynthas corticis
Clitellata
Capitella teleta
Polychaeta
Olavius tantalus
Olavius tantalus
Olavius tantalus
Olavius tantalus
Olavius imperfectus
Olavius imperfectus
Olavius imperfectus
Olavius imperfectus
Olavius imperfectus
Olavius ilvae
Olavius ilvae
Inanidrilus leukodermatus
Inanidrilus sp. 'nysp'
Olavius ilvae
Olavius ilvae
Olavius ilvae
Olavius ilvae
Olavius ilvae
Olavius ilvae
Olavius ilvae
Olavius ilvae
Olavius ilvae
Olavius imperfectus
Olavuis algarvensis
Olavius algarvensis
Olavius finitimus
Olavius finitimus
Olavius finitimus
Olavius tantalus
Olavius tantalus
Olavius tantalus
Olavius tantalus
Inanidrilus sp. 'fant'
Inanidrilus sp. 'fant'
Inanidrilus sp. 'nysp'
Inanidrilus sp. 'nysp'
Inanidrilus sp. 'guadeloupe'
Inanidrilus leukodermatus
Olavius tantalus
Inanidrilus sp. 'fant'
Inanidrilus sp. 'fant'
Inanidrilus sp. 'fant'
Inanidrilus sp. 'fant'
Olavius finitimus
Olavius finitimus
Olavius finitimus
Inanidrilus sp. 'ule'
Inanidrilus sp. 'ule'
Inanidrilus sp. 'ule'
Inanidrilus sp. 'ule'
Inanidrilus sp. 'ule'
Inanidrilus sp. 'ule'
Inanidrilus sp. 'ule'
Clitellata
Capitella teleta
Capitella teleta
Polychaeta
Arthropoda
Tree scale: 1
Bootstrap > 95

Supplementary Figure 19 | Annelid PHADs do not fully recapitulate phylogeny within this phylum, likely because of multiple PHAD homologs in some clitellates. A maximum likelihood phylogenetic tree inferred using IQ TREE with ultrafast bootstrap (1000 replicates) shows that annelid PHADs segregate into four subclades. Rather than clustering strictly according to established annelid relationship, PHADs from some clitellate species are distributed across multiple branches. This pattern may reflect the presence of multiple PHAD homologs in clitellates, ancient duplication followed by differential loss, or limited phylogenetic resolution.

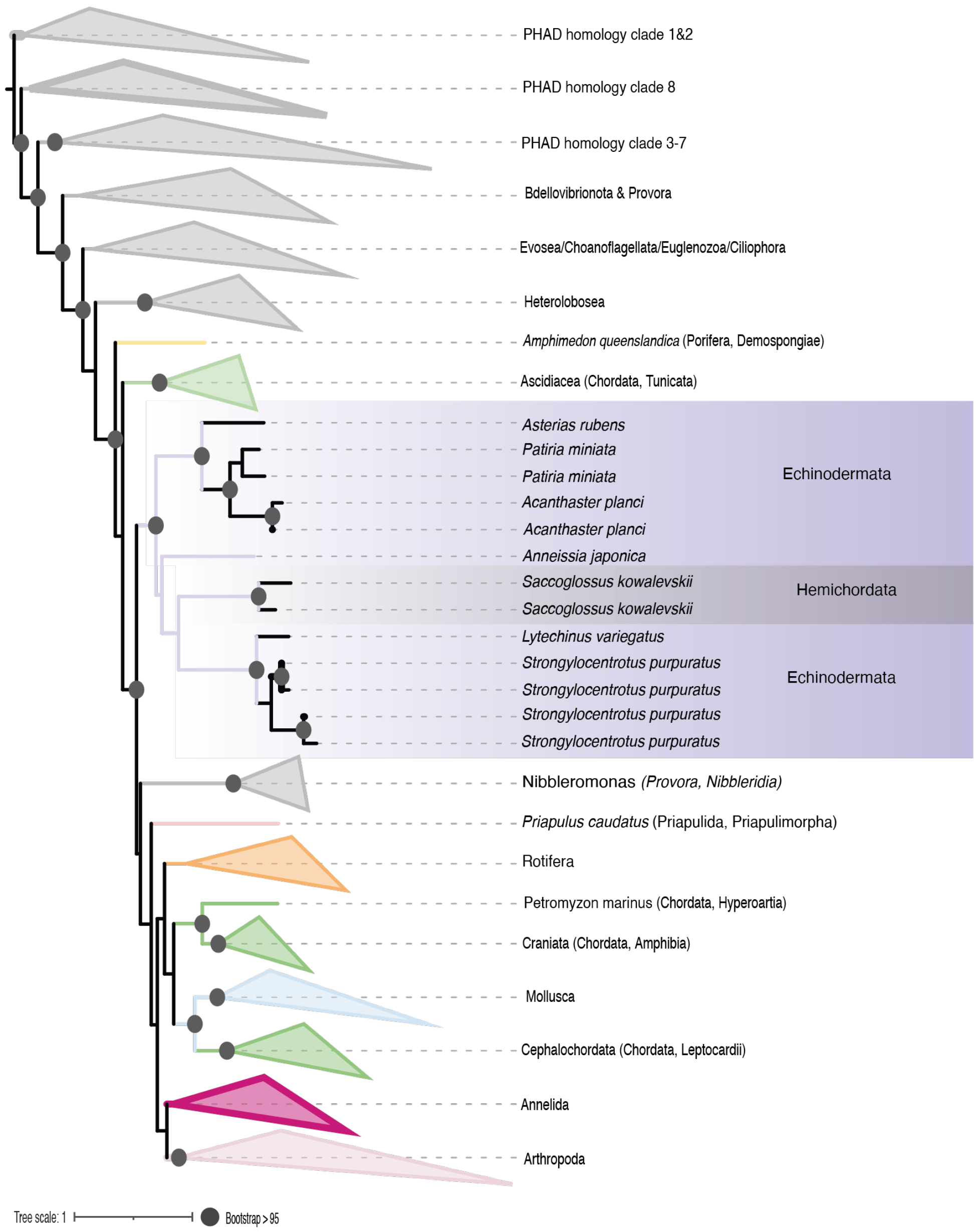

Supplementary Figure 20 | Echinoderm and hemichordate PHADs form a monophyletic group. A maximum likelihood phylogenetic tree inferred using IQ TREE with ultrafast bootstrap (1000 replicates) shows that PHADs from Echinodermata and Hemichordata PHADs cluster together in a single clade, consistent with their established close evolutionary relationship within the clade Ambulacraria.

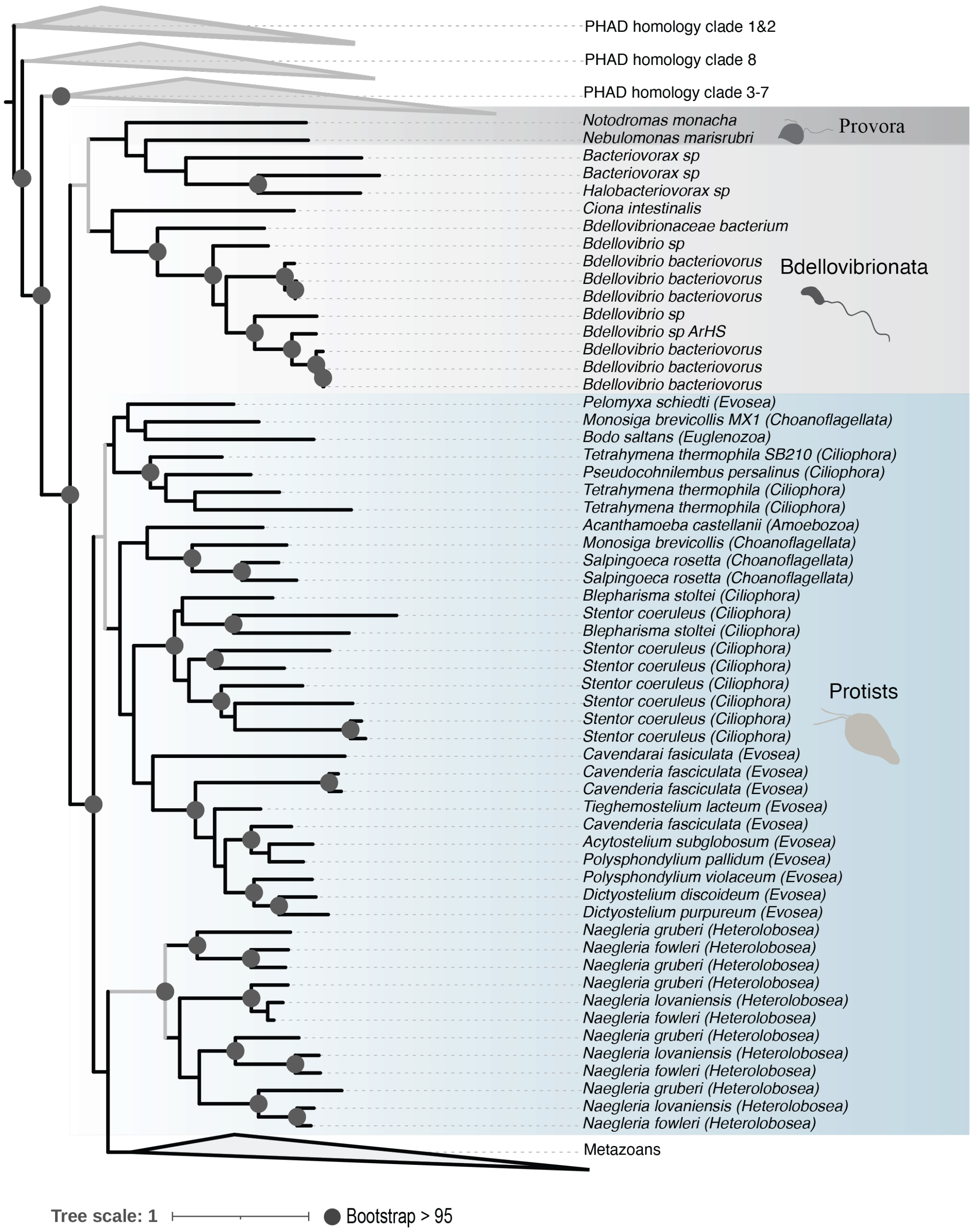
PHAD homology clade 1&2
PHAD homology clade 8
PHAD homology clade 3-7
Notodromas monacha
Nebulomonas marisrubri
Provora
Bacteriovorax sp
Bacteriovorax sp
Halobacteriovorax sp
Ciona intestinalis
Bdellovibrionaceae bacterium
Bdellovibrio sp
Bdellovibrio bacteriovorus
Bdellovibrionata
Bdellovibrio bacteriovorus
Bdellovibrio bacteriovorus
Bdellovibrio sp
Bdellovibrio sp ArHS
Bdellovibrio bacteriovorus
Bdellovibrio bacteriovorus
Bdellovibrio bacteriovorus
Pelomyxa schiedti (Evosea)
Monosiga brevicollis MX1 (Choanoflagellata)
Bodo saltans (Euglenozoa)
Tetrahymena thermophila SB210 (Ciliophora)
Pseudocohnilembus persalinus (Ciliophora)
Tetrahymena thermophila (Ciliophora)
Tetrahymena thermophila (Ciliophora)
Acanthamoeba castellanii (Amoebozoa)
Monosiga brevicollis (Choanoflagellata)
Salpingoeca rosetta (Choanoflagellata)
Salpingoeca rosetta (Choanoflagellata)
Blepharisma stoltei (Ciliophora)
Stentor coeruleus (Ciliophora)
Blepharisma stoltei (Ciliophora)
Stentor coeruleus (Ciliophora)
Stentor coeruleus (Ciliophora)
Stentor coeruleus (Ciliophora)
Protists
Stentor coeruleus (Ciliophora)
Stentor coeruleus (Ciliophora)
Stentor coeruleus (Ciliophora)
Cavendarai fasiculata (Evosea)
Cavenderia fasciculata (Evosea)
Cavenderia fasciculata (Evosea)
Tieghemostelium lacteum (Evosea)
Cavenderia fasciculata (Evosea)
Acytostelium subglobosum (Evosea)
Polysphondylium pallidum (Evosea)
Polysphondylium violaceum (Evosea)
Dictyostelium discoideum (Evosea)
Dictyostelium purpureum (Evosea)
Naegleria gruberi (Heterolobosea)
Naegleria fowleri (Heterolobosea)
Naegleria gruberi (Heterolobosea)
Naegleria gruberi (Heterolobosea)
Naegleria lovaniensis (Heterolobosea)
Naegleria fowleri (Heterolobosea)
Naegleria gruberi (Heterolobosea)
Naegleria lovaniensis (Heterolobosea)
Naegleria fowleri (Heterolobosea)
Naegleria gruberi (Heterolobosea)
Naegleria lovaniensis (Heterolobosea)
Naegleria fowleri (Heterolobosea)
Metazoans
Tree scale: 1
Bootstrap > 95

Supplementary Figure 21 | Bacterial *Bdellovibrio* and protistan Provora PHADs form a sister clade to animal and protist PHADs. A maximum likelihood phylogenetic tree inferred using IQ TREE with ultrafast bootstrap support (1000 replicates) shows that PHADs from Bdellovibrionata form a clade that is sister to PHADs from protists and metazoans. PHADs from two Provora species cluster with *Bdellovibrio* rather than with other protists. Both *Bdellovibrio* and Provora are predators (*Bdellovibrio* on bacteria, Provora on protists). Their close physical interactions with their prey are compatible with an ancient horizontal gene transfer scenario in which a PHAD gene may have been transferred between predator and prey lineages. The coherence and deep divergence of this clade argue against recent or repeated prey-to-predator transfer events. However, alternative explanations such as vertical inheritance followed by differential loss or limited resolution at deep evolutionary nodes cannot be excluded.

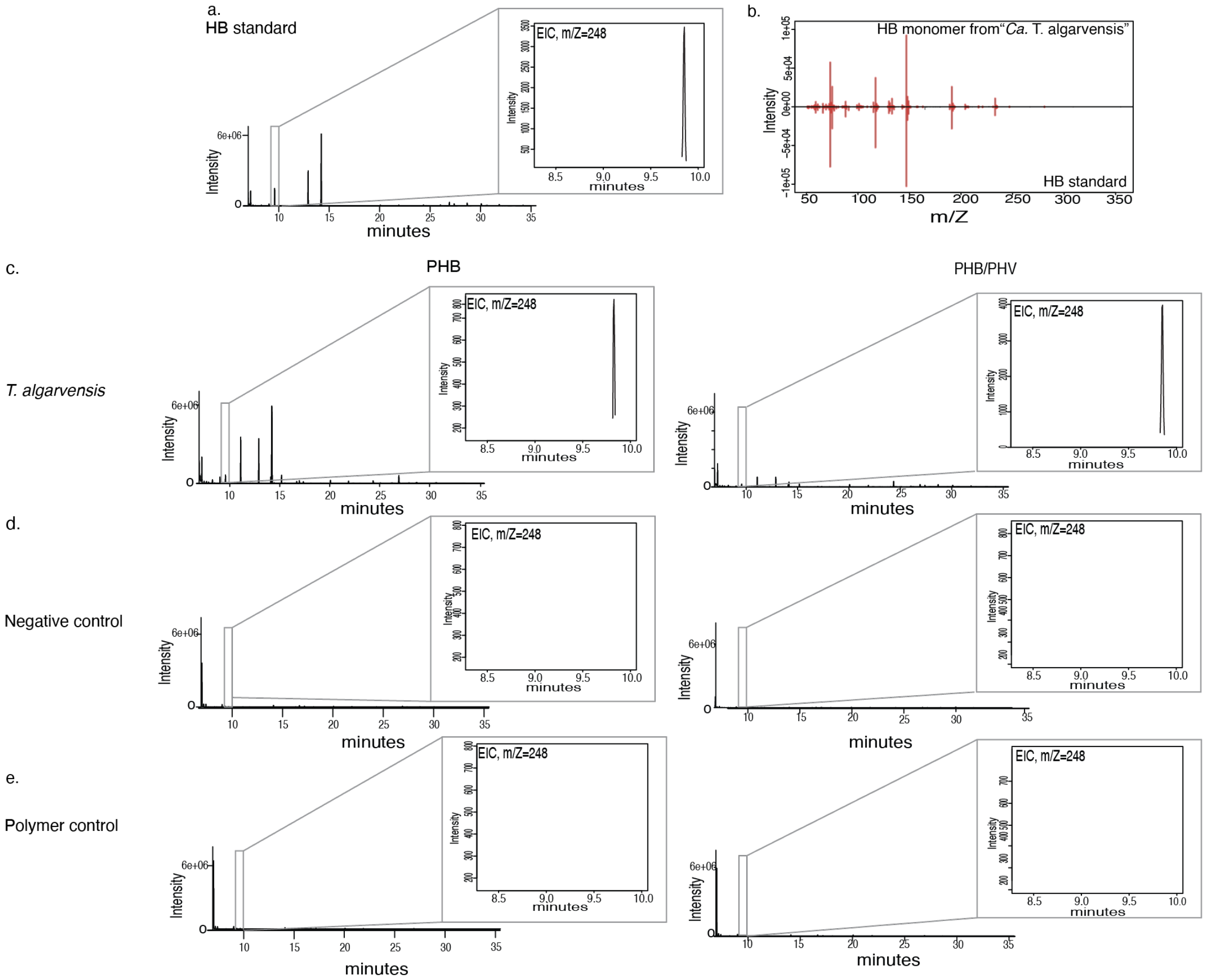

Supplementary Figure 22 | PHAD from *T. algarvensis* degrades PHB and PHB/PHV into HB monomers. a,c,d,e, GC-MS chromatograms of the HB standard, PHAD assays using PHB and PHB/PHV, the no-enzyme control and the negative control with a heat-killed PHAD. No HB monomers were detected in the two controls. Insets in (**a,c,d,e**) show extracted ion chromatograms (m/z 248) corresponding to the HB monomer. **b,** Fragmentation spectra show that HB monomers produced in assays with the *T. algarvensis* PHAD match the retention time (9.56 min) and mass spectrum of the HB standard.

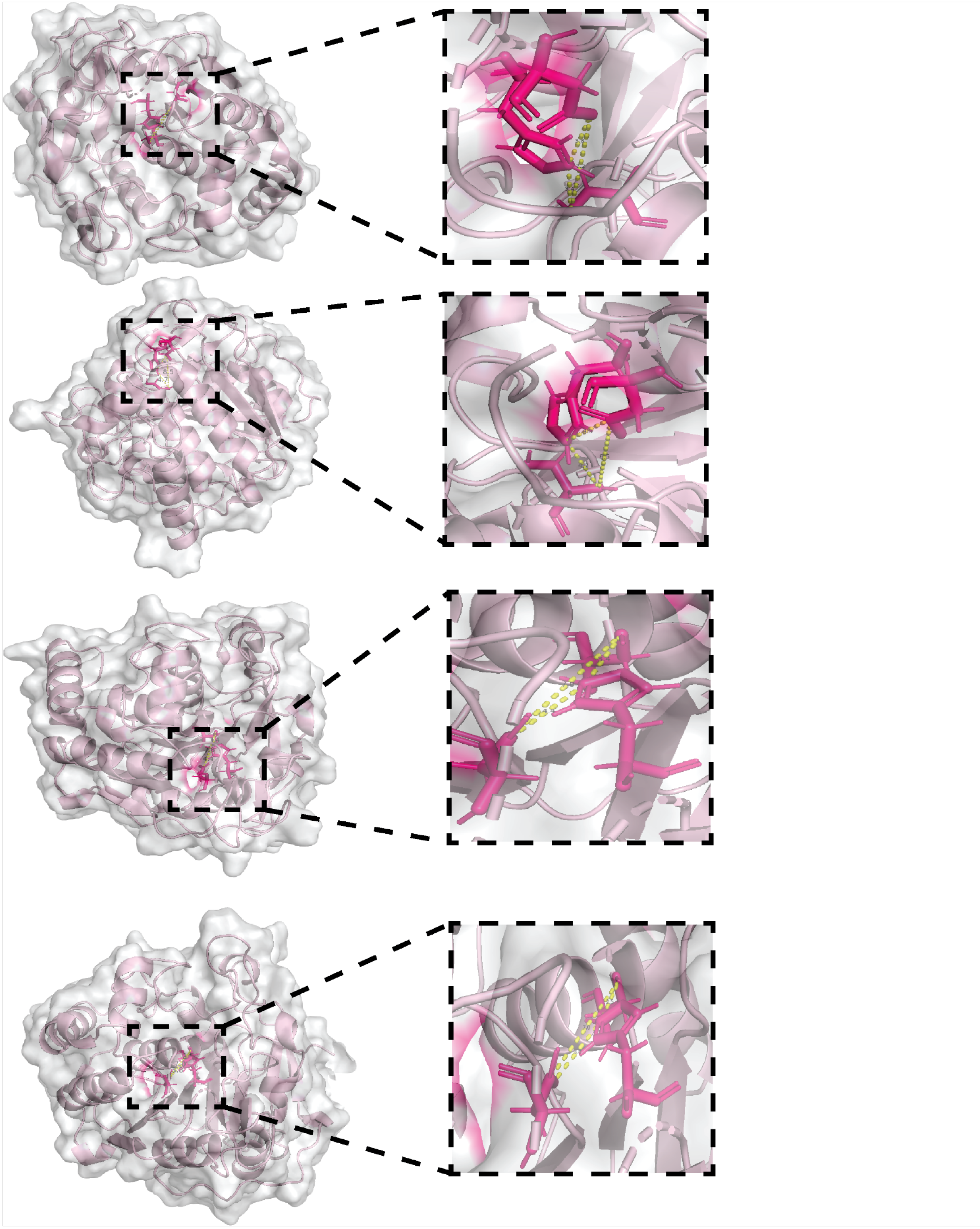

Supplementary Figure 23 | PHAD homologs from *Daphnia magna* show variation in the catalytic region. AlphaFold2 models of the four *D. magnas*'s PHAD homologs reveal differences in the configuration of residues surrounding the catalytic triad. Specifically, the two residues in the substrate binding region immediately following the catalytic aspartate ($D_{139/123/157}$) are substituted by either $SV_{140\text{-}141}$ or $ST_{124\text{-}125/158\text{-}159}$. This variation may influence substrate binding, and is consistent with the possibility that different PHAD homologs interact with distinct sPHA substrates.

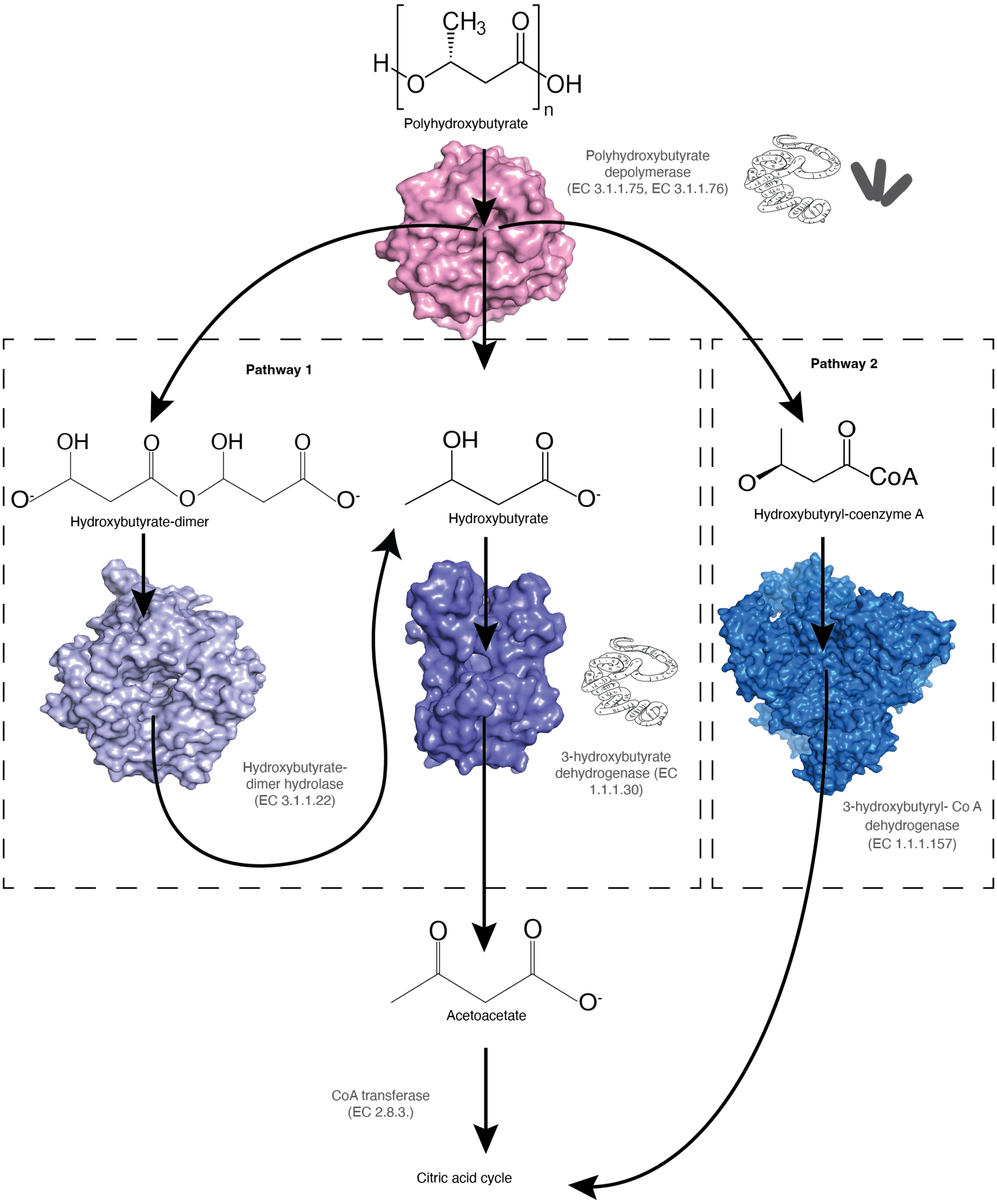

Polyhydroxybutyrate
Polyhydroxybutyrate depolymerase (EC 3.1.1.75, EC 3.1.1.76)
Pathway 1
Pathway 2
Hydroxybutyrate-dimer
Hydroxybutyrate
Hydroxybutyryl-coenzyme A
Hydroxybutyrate-dimer hydrolase (EC 3.1.1.22)
3-hydroxybutyrate dehydrogenase (EC 1.1.1.30)
3-hydroxybutyryl- Co A dehydrogenase (EC 1.1.1.157)
Acetoacetate
CoA transferase (EC 2.8.3.)
Citric acid cycle

Supplementary Figure 24 | Overview of enzymes and pathways involved in sPHA degradation. PHADs depolymerize PHB into hydroxybutyrate (HB) monomers, dimers or a mixture of oligomers. In **Pathway 1**, hydroxybutyrate-dimer hydrolase (EC 3.1.1.22) converts the dimers into their monomers. The resulting HB monomers are then oxidized to acetoacetate by β-hydroxybutyrate dehydrogenase (BHBD, EC 1.1.1.30; PDB 3w8e), after which acetoacetate is converted to acetyl coenzyme-A by CoA-transferase (2.8.3) and enters the citric acid cycle. Alternatively, in **Pathway 2**, PHAD activity produces CoA-bound monomers that are directly converted to acetyl-CoA by 3-hydroxybutyryl-coenzyme A dehydrogenase (EC 1.1.1.157; PDB 6acq). In our study, all gutless oligochaete species expressed a PHAD, and eight of the ten species expressed a BHBD. Their primary symbionts lacked a BHBD, a hydroxybutyrate dimer hydrolase and 3-hydroxybutyryl-coenzyme A dehydrogenase, indicating the absence of the canonical downstream enzymes required to catabolize the products of PHAD activity.

References

1. Hiraishi, T., Y. Hirahara, Y. Doi, M. Maeda and S. Taguchi, *Effects of mutations in the substrate-binding domain of poly[(R)-3-hydroxybutyrate](PHB) depolymerase from* Ralstonia pickettii *T1 on PHB degradation.* Applied and Environmental Microbiology, 2006. **72**(11): p. 7331-7338.
2. Chen, H.-J., S.-C. Pan and G.-C. Shaw, *Identification and characterization of a novel intracellular poly(3-hydroxybutyrate) depolymerase from* Bacillus megaterium. Applied and Environmental Microbiology, 2009. **75**(16): p. 5290-5299.
3. de Eugenio, L.I., P. Garci, J.M. Luengo, J. San Roma and J.L. Garci, *Biochemical evidence that* phaZ *gene encodes a specific intracellular medium chain length polyhydroxyalkanoate depolymerase in* Pseudomonas putida *KT2442: characterization of a paradigmatic enzyme.* Journal of Biological Chemistry, 2007. **282**(7): p. 4951-4962.
4. Hisano, T., K. Kasuya, Y. Tezuka, N. Ishii, T. Kobayashi, M. Shiraki, E. Oroudjev, H. Hansma, T. Iwata, Y. Doi, T. Saito and K. Miki, *The crystal structure of polyhydroxybutyrate depolymerase from* Penicillium funiculosum *provides insights into the recognition and degradation of biopolyesters.* Journal of Molecular Biology, 2006. **356**(4): p. 993-1004.
5. Braaz, R., R. Handrick and D. Jendrossek, *Identification and characterisation of the catalytic triad of the alkaliphilic thermotolerant PHA depolymerase* PhaZ7 *of* Paucimonas lemoignei. FEMS Microbiology Letters, 2003. **224**(1): p. 107-12.
6. Teufel, F., J.J. Almagro Armenteros, A.R. Johansen, M.H. Gíslason, S.I. Pihl, K.D. Tsirigos, O. Winther, S. Brunak, G. von Heijne and H. Nielsen, *SignalP 6.0 predicts all five types of signal peptides using protein language models.* Nature Biotechnology, 2022. **40**(7): p. 1023-1025.
7. Ott, J., S. Bulgheresi, H. Gruber-Vodicka, A. Gruhl, L. König and N. Leisch, *Meiofauna meets microbes - Chemosynthetic symbioses*, in New Horizons in Meiobenthos Research: Profiles, Patterns and Potentials. 2023, Springer. p. 79-119.
8. Jensen, M., J. Wippler and M. Kleiner, *Evaluation of RNAlater as a Field-Compatible Preservation Method for Metaproteomic Analyses of Bacterium-Animal Symbioses.* Microbiology Spectrum, 2021. **9**(2): p. e01429-21.
9. Wiśniewski, J.R., *Filter aided sample preparation–a tutorial.* Analytica Chimica Acta, 2019. **1090**: p. 23-30.
10. Uchino, K., T. Saito, B. Gebauer and D. Jendrossek, *Isolated poly(3-hydroxybutyrate)(PHB) granules are complex bacterial organelles catalyzing formation of PHB from acetyl coenzymeA (CoA) and degradation of PHB to acetyl-CoA.* Journal of Bacteriology, 2007. **189**(22): p. 8250-8256.
11. Eggers, J. and A. Steinbüchel, *Poly(3-hydroxybutyrate) degradation in* Ralstonia eutropha *H16 is mediated stereoselectively to (S)-3-hydroxybutyryl coenzyme A (CoA) via crotonyl-CoA.* Journal of Bacteriology, 2013. **195**(14): p. 3213-3223.
12. Sznajder, A. and D. Jendrossek, *To be or not to be a poly(3-hydroxybutyrate)(PHB) depolymerase:* PhaZd1 *(*PhaZ*6) and* PhaZd2 *(*PhaZ*7) of* Ralstonia eutropha*, highly active PHB depolymerases with no detectable role in mobilization of accumulated PHB.* Applied and Environmental Microbiology, 2014. **80**(16): p. 4936-4946.
13. Abe, T., T. Kobayashi and T. Saito, *Properties of a novel intracellular poly(3-hydroxybutyrate) depolymerase with high specific activity* (PhaZd*) in* Wautersia eutropha *H16.* Journal of Bacteriology, 2005. **187**(20): p. 6982-6990.
14. Tanio, T., T. Fukui, Y. Shirakura, T. Saito, K. Tomita, T. Kaiho and S. Masamune, *An extracellular poly(3-hydroxybutyrate) depolymerase from* Alcaligenes faecalis. European Journal of Biochemistry, 1982. **124**(1): p. 71-77.

15. Jendrossek, D., I. Knoke, R.B. Habibian, A. Steinbüchel and H.G. Schlegel, *Degradation of poly(3-hydroxybutyrate), PHB, by bacteria and purification of a novel PHB depolymerase from* Comamonas sp. Journal of Environmental Polymer Degradation, 1993. **1**(1): p. 53-63.
16. Senior, P.J. and E.A. Dawes, *The regulation of poly-beta-hydroxybutyrate metabolism in* Azotobacter beijerinckii. Biochemical Journal, 1973. **134**(1): p. 225-38.
17. Prieto, M.A., L.I.d. Eugenio, B. Galán, J.M. Luengo and B. Witholt, *Synthesis and degradation of polyhydroxyalkanoates*, in Pseudomonas. 2007, Springer. p. 397-428.
18. Doi, Y., Y. Kawaguchi, N. Koyama, S. Nakamura, M. Hiramitsu, Y. Yoshida and H. Kimura, *Synthesis and degradation of polyhydroxyalkanoates in* Alcaligenes eutrophus. FEMS Microbiology Reviews, 1992. **9**(2-4): p. 103-108.
19. Schirmer, A., D. Jendrossek and H.G. Schlegel, *Degradation of poly(3-hydroxyoctanoic acid)[P(3HO)] by bacteria: purification and properties of a P(3HO) depolymerase from* Pseudomonas fluorescen*s GK13.* Applied and Environmental Microbiology, 1993. **59**(4): p. 1220-1227.
20. Nakayama, K., T. Saito, T. Fukui, Y. Shirakura and K. Tomita, *Purification and properties of extracellular poly(3-hydroxybutyrate) depolymerases from* Pseudomonas lemoignei. Biochimica et Biophysica Acta (BBA)-Protein Structure and Molecular Enzymology, 1985. **827**(1): p. 63-72.
21. Shirakura, Y., T. Fukui, T. Tanio, K. Nakayama, R. Matsuno and K. Tomita, *An extracellular D(−)-3-hydroxybutyrate oligomer hydrolase from* Alcaligenes faecalis. Biochimica et Biophysica Acta (BBA)-Protein Structure and Molecular Enzymology, 1983. **748**(2): p. 331-339.
22. Delafield, F., K.E. Cooksey and M. Doudoroff, *β-Hydroxybutyric dehydrogenase and dimer hydrolase of* Pseudomonas lemoignei. Journal of Biological Chemistry, 1965. **240**(10): p. 4023-4028.
23. Lehninger, A.L., H. Sudduth and J.B. Wise, *D-β-hydroxybutyric dehydrogenase of mitochondria.* Journal of Biological Chemistry, 1960. **235**(8): p. 2450-2455.
24. Anderson, I.J., R.F. Watkins, J. Samuelson, D.F. Spencer, W.H. Majoros, M.W. Gray and B.J. Loftus, *Gene discovery in the* Acanthamoeba castellanii *genome.* Protist, 2005. **156**(2): p. 203-14.
25. Burki, F., A.J. Roger, M.W. Brown and A.G. Simpson, *The new tree of eukaryotes.* Trends in Ecology & Evolution, 2020. **35**(1): p. 43-55.
26. Mankowski, A., M. Kleiner, C. Erséus, N. Leisch, Y. Sato, J.-M. Volland, B. Huettel, C. Wentrup, T. Woyke, J. Wippler, N. Dubilier and H. Gruber-Vodicka, *Repeated losses and gains of bacterial symbionts in gutless marine annelids over 150 million years.* BioRxiv, 2021-04.
27. Nikolenko, S.I., A.I. Korobeynikov and M.A. Alekseyev, *BayesHammer: Bayesian clustering for error correction in single-cell sequencing.* BMC Genomics, 2013. **14**(Suppl 1): p. S7.
28. Andrews, S., *FastQC: a quality control tool for high throughput sequence data*. 2010, Cambridge, United Kingdom.
29. Gruber-Vodicka, H.R., B.K. Seah and E. Pruesse, *phyloFlash: rapid small-subunit rRNA profiling and targeted assembly from metagenomes.* Msystems, 2020. **5**(5): p. 10.1128/msystems. 00920-20.
30. Prjibelski, A., D. Antipov, D. Meleshko, A. Lapidus and A. Korobeynikov, *Using SPAdes* de novo *assembler.* Current Protocols in Bioinformatics, 2020. **70**(1): p. e102.
31. Bushnell, B., *BBMap: a fast, accurate, splice-aware aligner*. 2014, Lawrence Berkeley National Lab.(LBNL), Berkeley, CA (United States).

32. Kang, D.D., F. Li, E. Kirton, A. Thomas, R. Egan, H. An and Z. Wang, *MetaBAT 2: an adaptive binning algorithm for robust and efficient genome reconstruction from metagenome assemblies.* PeerJ, 2019. **7**: p. e7359.
33. Chklovski, A., D.H. Parks, B.J. Woodcroft and G.W. Tyson, *CheckM2: a rapid, scalable and accurate tool for assessing microbial genome quality using machine learning.* Nature Methods, 2023. **20**(8): p. 1203-1212.
34. Wiśniewski, J.R., A. Zougman, N. Nagaraj and M. Mann, *Universal sample preparation method for proteome analysis.* Nature Methods, 2009. **6**(5): p. 359-362.
35. Kleiner, M., X. Dong, T. Hinzke, J. Wippler, E. Thorson, B. Mayer and M. Strous, *Metaproteomics method to determine carbon sources and assimilation pathways of species in microbial communities.* Proceedings of the National Academy of Sciences, 2018. **115**(24): p. E5576-E5584.
36. Blakeley-Ruiz, J.A. and M. Kleiner, *Considerations for constructing a protein sequence database for metaproteomics.* Computational and Structural Biotechnology Journal, 2022. **20**: p. 937-952.
37. Li, W. and A. Godzik, *Cd-hit: a fast program for clustering and comparing large sets of protein or nucleotide sequences.* Bioinformatics, 2006. **22**(13): p. 1658-1659.
38. Hotelier, T., L. Renault, X. Cousin, V. Negre, P. Marchot and A. Chatonnet, *ESTHER, the database of the α/β-hydrolase fold superfamily of proteins.* Nucleic Acids Research, 2004. **32**(suppl_1): p. D145-D147.
39. Katoh, K. and D.M. Standley, *MAFFT multiple sequence alignment software version 7: improvements in performance and usability.* Molecular Biology and Evolution, 2013. **30**(4): p. 772-780.
40. Minh, B.Q., H.A. Schmidt, O. Chernomor, D. Schrempf, M.D. Woodhams, A. von Haeseler and R. Lanfear, *IQ-TREE 2: New Models and Efficient Methods for Phylogenetic Inference in the Genomic Era.* Molecular Biology and Evolution, 2020. **37**(5): p. 1530-1534.
41. Letunic, I. and P. Bork, *Interactive Tree Of Life (iTOL) v5: an online tool for phylogenetic tree display and annotation.* Nucleic Acids Research, 2021. **49**(W1): p. W293-W296.
