# Supplementary material for: Animal degradation of microbial storage polyhydroxyalkanoates
Source: Nat Ecol Evol. 2026 Aug 13;10(10):1823–37. doi: 10.1038/s41559-026-03153-8 (PMC13642032; doi:10.1038/s41559-026-03153-8)
Supplement: Supplementary file 3 [file 41559_2026_3153_MOESM3_ESM.pdf]

nature portfolio

Peer Review File

Animal degradation of microbial storage polyhydroxyalkanoates

Corresponding Author: Professor Nicole Dubilier

Version 0:

Reviewer comments:

Reviewer #1

(Remarks to the Author)
The authors addressed all of my previous concerns and took my comments to heart. I belive the enzymatic assay added to the results and now shows more convincingly that the enzymes are active against PHAs. I have no further comments to the authors. Thank you for a very interesting piece of work.

Reviewer #2

(Remarks to the Author)
The authors have largely revised the previously submitted manuscript and convincingly have addressed most of the concerns raised by the reviewer(s). The authors show that eukaryotes (here gutless worms) have (active) PHA depolymerases that contribute to the digestion and utilization of bacterial symbionts including the PHA moiety accumulated by the symbionts. This contribution substantially broadens our knowledge on PHA depolymerases and highlight that PHA degradation is not limited to prokaryotes and single cell microorganisms. I therefore support publication of this important contribution.

One minor suggestion:
Main text, lines 111-117 and Suppl. Text, lines 23-46: please provide also similarity values (not only identity values) of the aligned proteins. Perhaps a suppl. Fig. showing the two aligned (domain) amino acid sequences would be also good.

Reviewer #3

(Remarks to the Author)
I was reviewer # 3 last time and had criticized the lack of functional proof with respect to the predicted enzyme functions. After reading through the manuscript and the response letter I can acknowledge that the authors have taken most of my comments into consideration and addressed them.
Using MS technologies but also halo formation and after overexpressing selected genes and enzymes they now have preliminary data that strongly suggest that the predicted enzymes are functional and involved in PHA transformation.
While this is positive, strong and solid kinetic data on enzyme turnover would be favorable.
I would urge the authors to clearly state that the substrates converted might not be the natural substrates of the enzymes detected. It is well known that alpha-beta hydrolases are highly promiscuous enzymes and that they often randomly turn over – albeit at very low rates - a very wide range of different substrates. The literature indicates that some of these enzymes may have the potential to degrade over 80 different substrates. So it remains questionable if the PHAs are the native and given the small halo forming activities.
Nevertheless this is a very interesting and well written manuscript that makes an important contribution to the field.

Open Access This Peer Review File is licensed under a Creative Commons Attribution 4.0 International License, which permits use, sharing, adaptation, distribution and reproduction in any medium or format, as long as you give appropriate credit to the original author(s) and the source, provide a link to the Creative Commons license, and indicate if changes were made.
In cases where reviewers are anonymous, credit should be given to 'Anonymous Referee' and the source.
The images or other third party material in this Peer Review File are included in the article's Creative Commons license, unless indicated otherwise in a credit line to the material. If material is not included in the article's Creative Commons license and your intended use is not permitted by statutory regulation or exceeds the permitted use, you will need to obtain permission directly from the copyright holder.
To view a copy of this license, visit https://creativecommons.org/licenses/by/4.0/

Response to the Reviewers

Manuscript: "Animals degrade microbial storage polyhydroxyalkanoates"

Authors: Caroline Zeidler[1], Harald Gruber-Vodicka[1,2], Dolma Michellod[1], M. Grace D'Angelo[1], Stefan Becker[3], Henry Berndt[2], Juliane Wippler[1], Marlene Violette[4], Manuel Kleiner[4], Alba Porta-Fidalgo[1], Rebekka S. Janke[1], Kristina Weinert[1], Manuel Liebeke[1,5], Nicole Dubilier[1*], Maggie Sogin[1,6*]

[1]Max Planck Institute for Marine Microbiology, Celsiusstraße 1, 28359 Bremen, Germany

[2] Zoological Institute, University of Kiel, 24118 Kiel, Germany

[3] MARUM Center for Marine Environmental Sciences, University of Bremen, 28359 Bremen, Germany

[4] North Carolina State University, Department of Plant and Microbial Biology, 27607 Raleigh, North Carolina, USA

[5] Institute of Human Nutrition and Food Science, Department of Metabolomics, University of Kiel, 24118 Kiel, Germany

[6] University of California at Merced, Merced, CA 95343, USA

We thank all three reviewers and the Editor for their insightful and constructive feedback on our manuscript. We now have new data from additional experiments that we are confident will address the reviewers' concerns. Below a brief summary of our new findings, followed by our detailed point-by-point response to the referees. All revised figures are included at the end of our response.

While Referees #1 and #2 were supportive, Referee #3 raised two major concerns: "…..they fail to show that the enzyme is indeed a functional homolog. Further it has previously been reported that humans, mouse and other animals produce and degrade PHB-like polymers."

We now directly address the first concern of Referee #3 by showing that the polyhydroxyalkanoate depolymerase (PHAD) we identified in animals is a functional homolog of microbial PHADs. Using a new GC-MS-based enzyme assay, we demonstrate that heterologously expressed PHADs from four very distantly related animal species from both marine and terrestrial environments degrade high molecular weight storage PHAs (sPHAs) into hydroxyalkanoates, the characteristic product of PHAD activity.

The second concern of Referee #3 reflects a misunderstanding. Animals produce PHA-like polymers only as low molecular weight compounds with 30 to 200 hydroxyalkanoates complexed within membranes or covalently bound to membrane proteins, where they serve structural or regulatory roles. In contrast, microorganisms accumulate high molecular weight sPHAs with at least 1000 hydroxyalkanote units in intracellular granules for carbon and energy storage. Although low molecular weight PHAs have been studied for decades in animals, the enzymes that degrade them remain unknown. Importantly, our data show that animal PHADs

degrade microbial sPHAs into hydroxyalkanoates - not that they act on low molecular weight animal PHAs. This distinction underscores the novelty of our study.

Our revised manuscript, supported by new enzymatic, structural, and phylogenetic data, therefore provides a significant advance by identifying the first animal PHADs capable of degrading microbial sPHAs. These enzymes cluster with experimentally verified bacterial and fungal PHADs and are clearly distinct from other alpha/beta hydrolases, addressing the additional concerns raised by Referees #2 and #3.

Referees' comments

In our responses below to the referees' comments, we indicate the line numbers in the revised manuscript where each change can be found. Because of the number and length of the referees' comments, quoting the revised text in full would have substantially reduced the readability of this response. This approach allows reviewers to quickly and efficiently verify that all their comments have been fully addressed. All revised figures and legends are shown at the end of this response.

Referee #1 (Remarks to the Author):

The manuscript by Zeidler et al. reports on the discovery of enzymatic evidence for PHA degradation in gutless worms and other animals that either feed on PHA-producing symbionts or on soil/sediment rich in PHA. The authors reported the discovery of a PHA depolymerase (PHAD) in the transcriptome of the gutless worm Olavius algarvensis. They further identified the gene encoding the enzyme in the worm genome, heterologously expressed it in E.coli and used the purified enzyme in a spot assay to show that it indeed degraded PHA and other derived polymers. Homologs of the O. algarvensis PHAD were also identified in several other animal and protist species hinting at a more widespread occurrence of PHA degradation ability in animals and protists.

1. Innovation and Novelty: Results reported here are significant because they could be linked to the degradation of naturally produced PHA (bioplastics) in soil and aquatic environments and contribute to the carbon cycling in these habitats.

2. Methods and Results: The use of HCR-FISH to label the mRNA in the epidermal cells of *O. algarvensis* and the employment of two probes for the host and for the symbiont is innovative and convincing.

We thank the reviewer for their positive comments.

3. Going the extra mile to heterologously express the enzyme is commended. However, the spot assay figures were not clear. The authors report on positive PHA and PHA derivatives degradation based on these spot assays but I found it hard to see. It looks like the figures in Extended data 1 and in Suppl figure 15 are the plate pictures after incubation of the enzymes with PHA for 24 hours with zones of clearing. Maybe it would be helpful to show the plates before incubation and somehow point to these zones of clearing. The negative control

pictures were not helpful either. This seems to be an important part of the results and having it as extended data/ supplementary figure may not be the most appropriate.

We agree. As described in our brief summary above, we now have new data providing evidence that the heterologously expressed PHAD of *Olavius algarvensis* and three other animals (the earthworm *Lumbricus rubellus,* the sponge *Amphimedon queenslandica* and the springtail *Folsomia candida*) degrade sPHAs. We coupled the spot assays with a new approach we developed that uses gas chromatography - mass spectrometry (GC-MS) analyses to directly demonstrate that animal PHADs degrade sPHAs to hydroxyalkanoates (see revised Figure 2 & 5 and Supplementary Figure 2-4 & 12-14). Importantly, the relative activities of the animal PHADs fell within the same range as the bacterial PHAD we used as a positive control. Our results show that animal PHADs are not only comparable in efficiency to bacterial enzymes in degrading sPHAs, but also reveal that a capability previously thought to be restricted to microbes is widespread across animals (see Figure 2 & 5). These new results are highlighted in our updated figures, main text and the supplement.

4. Phylogeny of PHAD (trees in Fig 5 and Suppl Figs 8-14): More clarification is needed on the trees for the delineation of animals and protists. There was a lot of discussion and speculation on the presence/absence of PHAD in LCA of animals mainly based on these trees. For example, lines 256-262. While these assumptions are intriguing, I failed to see the support (at least based on the trees in the manuscript). It is fine to discuss these assumptions but I think with only a few copies of PHAD per clade and a few species of hosts per clade, these statements need to be toned down a bit.

We agree and toned down our statements. The limited number of animal and protist PHADs in publicly available databases limits strong statistical support for the deep-branching nodes between protist and animal PHADs. However, given the clear separation of protist and animal PHADs from bacterial PHADs, we hypothesize that the evolution of animal and protist PHADs were closely linked. We modified lines 371-379 accordingly.

5. The fact that downstream PHA degradation genes were also identified in the hosts but not the symbionts is interesting. The discussion on the strong selective advantage to the host makes sense. Maybe Figure 4 and Suppl Figure 18 can be swapped. I think the identification of BHBD transcripts expressed to the same level as PHAD is important and deserve to be in the main manuscript.

We agree and have replaced Figure 4 with Supplementary Figure 18 in the main text and now all data is presented in Figure 4. We also expanded our discussion of these findings in lines 241-317.

6. I am not an expert on AlphaFold or protein folding in general, and so I will not comment on this part of the results. However, from a non-expert opinion, I was convinced that the enzymes should be capable of binding to PHA, and, with the minor differences identified in the substrate binding pocket of some PHADs, also binding to PHA derivatives.

Agreed, the two other reviewers were also not critical of this part of our study.

7. Overall, I genuinely enjoyed reading this manuscript. The combination of transcriptomic analysis, HCR FISH, heterologous expression, enzyme assays, protein folding, and bioinformatic analyses helped deliver a complete story. I agree with the authors that this discovery could potentially change the way we think about carbon cycling by accounting for a largely ignored fraction of carbon stored in these PHA molecules present in natural environments.

Thank you for your supportive and constructive comments!

Referee #2 (Remarks to the Author):

Zeidler et al. suggest that polyhydroxybutyrate (PHB), that is accumulated by prokaryotic symbiont cells (Ca. Thiosymbion algarvensis) of the gutless worm Olavius algarvensis, is degraded and utilized by a worm-encoded PHB depolymerase during lysosomal digestion of the symbionts by the host. The author identified each one PHB depolymerase (phaZ) gene in O. algarvensis and a putative PHB depolymerase in Ca. T. algarvensis. PHB depolymerase of the eukaryotic host was related in primary amino acid sequence and in its predicted (alphafold) 3D-structure to the PHB depolymerase of the fungus Penicillium funiculosum. Enzymatic activity of PhaZ (PHAD) was confirmed in vitro using the heterologically (in E. coli) expressed gene and a commercial PHB sample. The putative prokaryotic PHB depolymerase was not investigated.

In the second part of this study Zeidler and co-workers provide evidence that PhaZ (PHAD)-related PHB depolymerase genes are present as well in all 14 investigated gutless worms (oligochaetes) but also in at least 66 gut-bearing animal species. PHB depolymerases are well-known in bacteria and in many Archaea but have been so far not detected in eukaryotic species except some fungal species and few protists.

In principle, I like this work, and the discovery of PHB depolymerases with ability to digest material that has been produced by a symbiont in eukaryotic species (other than fungi) deserves publication. However, the manuscript in its present form suffers from several inconsistencies as pointed out below exemplarily. In addition, several figures, fig. legends and many of the suppl. Figs/legends are hard to understand for an interested scientist.

Major comments:

1. A: For those reader that are not experts in oligochaetes biology it would be beneficial if the authors could spend a few more words on the physiology of the O. algarvensis and its symbiont Ca. T. algarvensis. After reading one of the previous publications of the authors and after an internet search I have learned that Ca. T. algarvensis is a chemolithoautotrophic sulfur (sulfide?) oxidizing species. Organic compounds produced by the chemolithoautotrophic lifestyle of the prokaryote are secreted and can be used by the host. In this study the authors state that the host digests the symbiont cells.

   When does this digestion take place? Is only a fraction of the symbiont cells digested? Or does digestion depend on unfavorable conditions?

We agree that providing a few sentences in the Introduction on the biology of the symbiosis is helpful and have now added these (lines 78-88), as well as providing references to more extensive descriptions of the symbiosis. To answer the reviewer's question here briefly: The symbionts are digested in the epidermis through phagolysosomal digestion, which occurs continuously (not only under unfavorable conditions), as this is the only way that hosts can gain nutrition.

B: I thought that the host relies on the metabolism of the symbionts but if the symbiont is digested, the gutless worm must die as well. Or are the symbionts continuously taken up by the host? Maybe this is well-known for O. algarvensis specialists but for a broader readership some explanation would be good.

As mentioned above, we now provide more information in the Introduction and refer to literature on the symbiosis. Briefly, there is a balance between symbiont division and host digestion that ensures a continuous supply of nutrition to the host[1].

C: The authors state that digestion happens by lysomal digestion. How is this digestion controlled (spacially?) Could it be that the PHB content of the symbionts helps the symbiont cells to (partially) resist lysomal digestion? The degradation products of PHB (3HB and 3HB oligomers, and corresponding methylesters) are strong antioxidants (better than gluthathione).

Please see Koskimäki et al.2016 (DOI: 10.1038/nchembio.2043).

Addressed as explained above. Briefly, the digestion of the symbionts in phagolysosomes is well documented[1], but the enzymatic processes involved remain unknown. Also, thank you for pointing out this interesting paper. While it is conceivable that sPHA degradation products could partially inhibit symbiont digestion, this would ultimately be detrimental to the host, which depends entirely on its symbionts for nutrition. Such a strategy would not be evolutionarily successful over the long term.

2. A: Line 80-82 (10.2% amino acid similarity). The value of 10.2% similarity is meaningless. In suppl. fig. S2 you have aligned ≈40 residues of two potential PHA depolymerases that presumably (according to fig 1a) are circular permutated (as PHA depolymerases of different subgroups often are). Furthermore, both sequences presumably belong to differ types of PHA depolymerases: the eukaryotic sequence is composed of only ≈320 amino acids, while the prokaryotic sequence is almost 900 residues "long". So you compare apples with pears. To find out whether the two sequences have (partial) amino acid similarities you should choose a bioinformatic approach with settings that are more appropriate to identify local (partial) similarities.

We agree and thank the reviewer for pointing this out. We now no longer compare the amino acid similarities of the sequences, and instead, as suggested, compare the phylogeny of the animal and symbiont PHADs and their structural differences (e.g., the circular permutation of the lipase box). We have modified Figure 2 (now Figure 1) to visualize the differences between the host and symbiont PHADs, changed the order of Figures 1 and 2, and removed Supplementary Figure 2 from the manuscript.

B: The manuscript provides hardly no information on the properties of the (putative) prokaryotic PHB depolymerase. Is there any related sequence available in the database that could give you a hint whether this protein is really a PHA depolymerase (intracellular or extracellular) and, if yes, are there examples available in literature of biochemically characterized PHA depolymerases related to the Ca. Thiosymbion algarvensis enzyme?

We agree that more information about the symbiont PHAD would be valuable and have now included experiments with heterologously expressed symbiont PHAD analyzed with our new GC-MS analyses (Figure 2, the main text in lines 111-117, and in the Supplement Figure 2, 13 & 22 and Text 1).

C: Is the enzyme also active (as the O. algarvensis one)?

Yes, it is also active, please see our preceding reply and revised figures and text.

D: Active with amorphous or crystalline PHA? The tested PHB depolymerase activity of the host PHB depolymerase was performed with (partially) crystalline and denatured PHB. The PHB material of the symbiont most likely is completely amorphous. Therefore, the host PHB depolymerase should be tested for activity with amorphous (native) PHB granules.

We agree that the symbionts' sPHA is likely amorphous within intact symbiont cells. However, once the host begins digesting its symbionts in phagolysosomes, the sPHA is highly unlikely to remain amorphous and instead becomes denatured upon release from the symbiont cell[2]. Because the host cannot degrade sPHA within intact symbionts - this would require importing its PHAD into bacterial cells, a process not known for any animal enzyme - degradation likely occurs within the phagolysosome on denatured sPHA. This interpretation is consistent with the phylogenetic placement of the gutless oligochaete PHADs within the dPHADscl clade (Figure 1), which comprises PHADs known to degrade denatured short chain sPHAs. We therefore consider the use of denatured PHA in our enzyme assays appropriate, as it best reflects the natural biological context in which the gutless oligochaete PHADs act. We now explain this reasoning in lines 151-173.

3. A: Fig. 2/suppl. fig. 6: I find these figs. not useful for the readership: you should explain what is meant with "PHADs of domain type 1" versus "PHADs of domain type 2". For most of the sequences in this fig. there is no information provided to which species the respective sequence refers. It would be great if this fig. would include and somehow indicate those sequences that refer to a protein for which the PHA depolymerase activity has been experimentally verified. Explain "no lipase box"; to my knowledge all PHA depolymerases have a lipase box (there might be variations in the degree of conservation).

We agree and revised accordingly (please see our revised figure below (now Figure 1). In the figure, we included the intracellular $nPHA_{SCL}$ depolymerases from *Cupriavidus necator*, which does not contain a lipase box. The experimentally validated enzyme (from *C. necator*) has a conserved catalytic asparagine and histidine residue but lacks the lipase box with the embedded catalytic serine[3].

B: Fig. 2/suppl. fig. 6 are almost identical.

We agree and have replaced Figure 2 (now Figure 1) with Supplementary Figure 6 in the main text.

4. A: Fig. 3: I have learned that Ca. T. algarvensis accumulates not only PHA (in form of small granules) but also large amounts of sulfur (also in form of granules). In the model shown in fig. 3d, however, only PHA granules are indicated. Why are sulfur globules not shown in fig. 3d? In the "true" EM-image (fig. 3I) three types of granule-like structures are visible in the symbiont cells: (i) large granules with grey appearance, (ii) granules of variable size with white appearance and (iii) small electron-dense granules (black appearance) often enriched in the cell periphery. I have found no comment on this in the manuscript and it is not evident for the reader which of the granule-like structures (i) or (ii) represent PHA granules.

We agree and have modified Figure 3 to now show the sulfur granules, and explain these in the figure legend.

B: Fig. 3e-g: The resolution and intensities of the fluorescence spot data are too low to clearly support the authors statement of asymmetric distribution of prokaryotic phaZ transcripts (green) and eukaryotic phaZ transcripts (pink). The green spots in fig. 3e are also visible in high numbers in the lower left part of the image. It is difficult to decide how much green spots are in the left upper part of the image because this part of the image seems to be a little bit out of focus and the signals are very weak. Fig. 3e-h are very dark and the fluorescence spots are hardly visible even on a computer screen and almost undetectable in a print-out. In particular, the distribution of spots is in some parts of the figs hardly detectable (and presumably out of focus).

We agree and have now included a new, additional figure that shows the expression of symbiont and host transcripts more clearly (Supplementary Figure 7).

C: I was wondering that in the suppl. fig.3 transcripts of prokaryotic and eukaryotic phaZ genes were NOT spatially separated. Citation from the legend to suppl. Fig. 3: The probes specific to PHAD transcripts of the symbiont "Ca. T. algarvensis" (green) and the host O. algarvensis (pink) are distinct from each other and are expressed throughout the worm's epidermis. This seems to be in contradiction to the statement in the main text (lines 142-145): The probes specific to the "Ca. T. algarvensis" PHAD hybridized to the symbionts, which occur just below the worm's cuticle and above its epidermal cells (Figure 3e, g, h). In contrast, the O. algarvensis PHAD probes hybridized to the epidermal cells directly below the symbionts (Figure 3f, g).

We agree that this was not written clearly and have revised accordingly (lines 200-207 of the main text).

D: The quality of the EM image seems to be rather low, at least for the eukaryotic part of the image. Which structures correspond to the organelles (mitochondria, ER, Golgi etc). Are the (three) dark grey structures the nuclei? I feel unable to corelate fig. 3i with the model 3d and

with the light microscopy image 3h. Is only one cell shown in fig. 3i? From the model (3d) I would expect to see two complete cells and small parts of two adjacent cells.

We intentionally used a lower-magnification TEM image to provide an overview of the spatial organization of the symbiosis and phagolysosomal digestion, rather than to resolve organelle details. High-resolution ultrastructural descriptions of these features are already well documented in previous studies on *O. algarvensis* and related gutless oligochaetes[4]. To improve the reader's ability to correlate between panels, we revised Figure 3d–i by adding labels for host and symbiont structures, adjusting the number and arrangement of cells, and including inset boxes indicating image positions.

E: EM images should have black/white/grey intensities corresponding to electron density. In Fig. 3i some structures are in yellow color (marked as lysosomes). Has color been added artificially? On what basis? Please explain.

We agree that this should be explained and have revised accordingly. In our previous manuscript, color was added to the TEM image in Figure 3i using standard false-coloring techniques to highlight specific host and symbiont structures and to facilitate comparison with the corresponding schematic model (Figure 3d). The underlying grayscale intensities corresponded to the original electron density data, and the color overlay was applied purely for illustrative clarity, not for quantitative interpretation. We have now revised the TEM image and added labels instead of false coloring to help the reader identify structures.

F: What does the reader see in fig3h? 7 or 8 individual eukaryotic cells of the epidermis? Please indicate specific cells. If this assumption is correct, the regular orientation of 7-8 epidermal cells in Fig. 3h is not detectable in Fig. 3i. (even talking into account that the magnification in 3i is higher than in 3h. The box in 3c "d&i" is misleading.

As mentioned in our responses to the reviewers' previous comments, we have now extensively revised Figure 3 to explain more clearly what is shown. Figure 3h shows fluorescent labeling of host nuclei (gray, DAPI) and "Candidatus *Thiosymbion algarvensis"* (hereafter *Thiosymbion algarvensis*) cells (yellow, 16S rRNA MiL-FISH), representing the upper symbiont layer just beneath the cuticle and above the epidermis. In contrast, Figure 3i depicts the cuticle (right) and underlying epidermis, identifiable by three host nuclei. To clarify orientation, we labeled key structures in both panels and added Supplementary Figure 7 illustrating the worm's anatomy and imaging approach.

G: Fig. 3h: It is difficult to decide which signals correspond to the Ca. Thiosymbiont cells in fig. 3h. I have the impression that the yellowish spots are located not only at the "right" side of the image (as suggested in the model in 3c) but more or less regularly in the whole cell periphery. Maybe I am wrong, but for me the image does not seem to support the authors conclusion that the symbiont cells are mainly located at the surface side (right side) of the epidermal cells. Please indicate the symbiont cells by arrows (or provide the DAPI signals, in the Method section (lines 511 und 517) it is stated that the material was also stained with DAPI.

Revised as suggested. As explained in our preceding responses, we have now extensively revised Figure 3 and Supplementary Figure 7.

H: line 527: method to fig. 3. Please check Volt intensities (10.000kV is unlikely; 10 kV meant?)

We thank the reviewer for spotting this typo and have corrected to 10 kV.

5. page 8 bottom: it is hard to imagine that the symbiont cells should not have (at least one) an enzyme for catabolism of the primary PHA depolymerase product. Most genomes have multiple dehydrogenase genes. The substrate specificities of these dehydrogenase enzymes are often low and the annotations of such genes/gene products might have a wrong "name" (and indicate a different substrate specificity). Please describe how you have verified that the genome does not contain a candidate for a potential 3HB-DH or 3HB-CoA-DH.

We agree that this is an unusual finding. In fact, we find this very interesting because it suggests that *T. algarvensis* may have lost these genes during its transition from a free-living to a symbiotic lifestyle. As described in Methods in lines 501–508, we searched the symbiont's metagenomically assembled genome (MAG) (92.4% complete, 1.49% contamination) and transcriptomes for genes encoding 3-BHBD, β-hydroxybutyrate oligomer hydrolase, and 3-hydroxybutyryl-CoA dehydrogenase using HMMER and a custom database including homologs from *Chromatiales* spp.. No significant hits (HMM score > 50) were detected. To account for potential gaps arising from MAG incompleteness or binning artifacts, we also BLASTed the *Chromatiales* homologs against the complete holobiont metagenomes. No significant hits were detected (identity < 50; coverage < 50), making it highly unlikely that all degradation genes would be absent from both nearly complete genomes and deeply sequenced metagenomes. Moreover, we searched the MAGs of the *Thiosymbion* symbionts from 14 other gutless oligochaete species, and found no homologues. This absence across all examined gutless oligochaete symbionts therefore likely reflects true gene loss. We present and discuss these findings in a thoroughly revised section called "Symbionts may not be able to metabolize the sPHAs they produce", and explain the selective benefit the hosts would gain through this gene loss (lines 250-303).

6. A: Line 66-67. "42% PHB content". The (high) PHB content of the symbiont was determined in a previous study of the corresponding author´s group. I was wondering why the PHB content in that study had been quantified by NMR technique. I am not a specialist in NMR and I can´t evaluate how reliable the value of 42% PHB content is. In studies on PHA accumulation in prokaryotes the amount of PHA is qualitatively and quantitatively determined by gas chromatography of samples (after conversion of PHB into monomeric methyl- or propionyl-esters of the corresponding hydroxyalkanoic acids).

We agree that PHA can be quantified using several methods, including GC-MS and NMR (see Koller et al. 2015 for a review of approaches, Linton et al. 2012 for use of NMR specifically)[5,6]. While GC-MS has the advantage that it is suitable for routine data collection, as the reviewer points out, it requires derivatization steps that limit the capacity to distinguish between heteropolyesters and blends of homopolyesters. NMR, on the other hand, can distinguish

between different types of PHA as it does not require derivatization. Consequently, in the study you mentioned[7], we used NMR to quantify the different forms of PHA, including PHV, PHmV and PHB (which is reported as the total PHA content of the worm).

B: In fig. 3, (see also comment 4 above) the symbiont cells are shown in a model (fig. 3d) and in an EM image (fig. 3i). However, it is unclear whether all granule like structures represent PHA/PHB. In literature on Ca. T. algarvensis large amounts of intracellular sulfur globules are frequently mentioned (in addition to PHB granules). It would be good to have quantitative values of PHB (and sulfur) for the oligochaetes used in this study.

We agree that quantitative estimates for sPHA and sulfur would be helpful and have now included these in lines 85-88 and 219-220.

7. lines 430-437 (primary structure analysis). "…we compared the recovered Ca. T. algarvensis…….PHAD amino acid sequences to the fungal homolog from P. funiculosum". It is not sufficient to compare a potential PHB depolymerase amino acid sequence with (only) the P. funiculosum sequence because PHA depolymerases of different subfamilies are not related in the primary amino acid sequences. It would be necessary to include representatives of other PHB depolymerase subfamily members as well.

We agree and have revised the main and supplementary text accordingly, as outlined in our response above to your Comment #2.

8. Line 435-436 "Novel amino and protist sequences that contained the complete catalytic site were predicted to be eukaryotic PHADs …." PHB depolymerases are serine hydrolases or hydrolases in which serine has been replaced by a cysteine and most – if not all of them – belong to the a/b-hydrolase family with a catalytic triad. However, not every a/b-hydrolase is a PHB depolymerase! Therefore, the presence of a (putative) catalytic site as the only criterion for the identification of a PHB depolymerase is not sufficient!

We fully agree that the presence of a catalytic site alone is insufficient to identify a PHAD. In our analysis, this motif served only as an initial screen. We then aligned putative animal sequences to the fungal PHAD homolog (*P. funiculosum*, PDB 2d81) and retained only those with full conservation of the catalytic triad, lipase box, and oxyanion hole. These sequences and those from the PHAD Engineering Database (DED)[2] were placed into a phylogenetic tree. Sequences that clustered within PHADs of the DED were considered animal PHADs and retained in our analysis. To exclude the possibility that the putative animal PHADs were not another type of a/b hydrolase, we generated additional phylogenetic trees of bacterial, fungal and animal PHADs with both lipases or cutinases. Sequences clustering with verified PHADs, but not with other a/b-hydrolases such as lipases or cutinases, were considered true PHADs. Finally, our newly added GC-MS analyses confirmed that heterologously expressed PHADs from four animal species degrade PHB and PHB/PHV into HB monomeric units, validating their functional annotation. We expanded our results to make these points clearer, now shown in Figure 5, Supplementary Figures 10-14 and described in lines 319-351.

Minor comments:

9. Line 40 (nitrogen and phosphate): please replace by ”nitrogen and phosphorous”.

Revised as suggested.

10. Line 48 (”The only known natural enzymes can degrade PHAs are called PHA depolymerase”). Some lipases and cutinases can (also) hydrolyze polyesters of hydroxyalkanoic acids with terminal OH-function in the monomer (no methyl or ethyl side group in the polymer).

We agree and have revised accordingly in lines 61-62 and 337-339.

11. Line 66/67 + line 62 suppl text: a copolymer of PHB and PHV would be a block-co-polymer. You probably mean a random copolymer of 3-hydroxybutyrate and 3-hydroxyvalerate. poly(3HB-co-3HV)

We agree and have revised line 86 in the main test and line 49 of the supplement.

12. Fig. 1a: This fig. is hard to understand: The red triangle (catalytic triad) is indicated at a certain position within the primary amino acid sequence. However, the three amino acids that form the catalytic triad are far from each other in the primary amino acid sequence. What you potentially mean is the position of the lipase box (G-x-S-x-G). Please provide a more clear figure. What is meant with the --S S--? Active site serine? Why two serines? Alternatively show the whole lipase box penta-sequence Please define and explain catalytic triad, oxyanion whole, substrate-binding site. The letters should have the same colors as the symbols above.

We agree and revised the figure (now Figure 2), also in response to Referee #1, Comment #3, to better highlight the representation of the enzyme’s primary structure.

13. Fig. 1b: In the zoom-in of the two structures five residues are indicated and highlighted by sticks. Please highlight only the catalytic triad residues.

We agree and have adjusted the figure (now Figure 2) to only show the catalytic triad residues as sticks (see Response to Reviewer #2, Comment #12).

14. Legend to fig1 is incomplete

We agree and have expanded the legend to include the full description of the figure (now Figure 2).

15. Line 90: "12 out of 15 individuals, indicating the relevance of this enzyme". One could also argue in an opposite way: 3 out of 15 individual did not have PHAD-specific mRNA suggesting that PHAD is not essential at least in three species.

The 15 transcriptome libraries included both total RNA and host-enriched RNA sequencing approaches. All host-enriched libraries (n = 3) contained the PHAD transcript, whereas the libraries lacking it were derived from total RNA, which often underrepresents host transcripts due to the dominance of rRNA and bacterial reads. We therefore interpret the absence of PHAD in these libraries as a likely sequencing bias rather than true absence of expression. In response to this comment, we have revised the text (lines 145-148) accordingly.

16. Legend to fig. 4: Please indicate the full genus name for the 5 strains from the right

Revised as suggested.

17. Line 231-233: "catalytic site, 100% identity. Since the catalytic site is just 1 residue (serine) the %-value is misleading. Do you mean the region around the catalytic site? Or the catalytic triad?

Revised. As explained on our response to your Comment #8, we are referring to the 100% identity of the catalytic triad and oxyanion hole.

18. Substrate binding site: How did you identify the substrate binding site? Only a few substrate binding domains from extracellular PHB depolymerases have been (experimentally) identified. For most PHB depolymerases that are specific for amorphous (native) PHB granules these substrate binding sites are more or less unknown/poorly defined.

To identify the substrate binding site, we compared the PHAD's primary structure to that of the experimentally validated PHAD from the fungus *P. funiculosum* (see our reply to your Comment #8). Because the animal PHAD was classified as a dPHADscl of domain type 2 (explained above in our reply to your Comment #2D), we did not look for binding sites specific to amorphous PHA.

19. Fig. 5: The legend is more an interpretation but a legend. Please explain the fig. in more detail. Some of the symbols are not explained. What is the meaning of different color intensities

We agree and have revised the legend accordingly.

20. Line 472: Reference 101. Please check if #101 is the correct reference

Reference #101 (now #118) is indeed the correct reference for the heterologous gene expression method we used in our manuscript.

21. Line 500. Define 5 x sodium chloride, sodium citrate?

We revised to clarify (lines 678-679)

Referee #3 (Remarks to the Author):

The article describes the identification of a novel hydrolase in the gutless oligochaete, Olavius algarvensis and that acts on PHA's, polyhydroxyalkanoates. The respective enzyme was designated PHAD. They further report PHAD homologues in other animals genomes and protists and they speculate that the last common ancestor of metazoans possessed a PHAD.

Polyhydroxyalkanoates are polymers that are widely produced by bacteria, archaea and eukaryotes. They differ in part in their structure because of the short chain fatty acid used. The polymers are degraded by de-polymerases that have been well characterized mostly in bacteria and some fungi. The authors provide preliminary evidence that Olavius algarvensis contains an enzyme that shows weak homologies to fungal and bacterial enzymes and that this enzyme may be able to hydrolyze PHB to some extent. In addition, some preliminary structural data are presented using alphafold2. The authors imply that by not studying the eukaryotic enzymes we have overseen a large faction within the carbon cycle.

Main critics:

1. The authors rely largely on preliminary data and over interpret the few biochemical data they present. This concerns especially the biochemical characterization and the structural prediction. While they observed a potential homologue of a bacterial PhaZ in their genome data set they fail to show that the enzyme is indeed a functional homologue. Further it has previously been reported that humans, mouse and other animals produce and degrade PHB-like polymers. Thus, the title needs to be changed.

As explained above at the beginning of this Response, this valid critique motivated us to add new experimental data demonstrating that PHADs from four very distantly related animal species, including terrestrial and marine species, degrade sPHA to HB monomers (gutless oligochaetes, sponges, springtails and earthworms) (Figure 2, Figure 5, Supplementary Figure 2 & 3 and Supplementary Figure 12-14; see also our responses above to Reviewer #1, Comment #3, and Reviewer #2, Comment 8).

2. Further it has previously been reported that humans, mouse and other animals produce and degrade PHB-like polymers. Thus, the title needs to be changed.

We apologize for not making this distinction clearer in our initial submission. Animals produce PHA-like polymers only as low molecular weight compounds that are bound to membranes or proteins, where they serve structural or regulatory functions (often referred to as complexed (cPHA) or oligomeric PHAs (oligo PHAs). In contrast, microorganisms produce high molecular weight polyhydroxyalkanoates (storage PHAs or sPHAs) that form visible intracellular granules

and function as carbon and energy reserves. Our study specifically focuses on high molecular weight, microbial sPHAs, which are not known from animals, and demonstrates for the first time that animals possess enzymes capable of degrading them. To reflect this distinction, we have revised the title to: "Animals degrade microbial storage polyhydroxyalkanoates".

3. Claims made on the occurrence of PHBs in animals are not correct: Polyhydroxyalkanoates are synthesized by bacteria, archaea and eukaryotes. In contrast what is written in the introduction their occurrence has been reported in plants, archaea, animals and even humans. Early reports by Leninger and his team implied already in the 1960s that humans produce and degrade PHB-like polymers doi: 10.1111/j.1432-1033.1994.00317.x; doi: 10.1155/2011/693253; doi: 10.1002/cbdv.201200294

Please see our response above and our revised Introduction in which we distinguish between low molecular weight PHAs found in animals and microbial sPHAs (lines 54-60).

4. Nevertheless, it is well-known that mammalian organisms (humans) produce and utilize poly-beta -hydroxybutyrates. The length of the polymers and the degree of polymerization appears to be shorter compared to bacteria; but they harbor enzymes to depolymerizes these. DOI: 10.1002/cbdv.201200278

We agree, as explained above in response to your previous comments. As noted above, mammals, including humans, have been reported to contain only low molecular weight cPHAs or oligo PHAs that differ fundamentally from microbial storage PHAs (sPHAs) in both size and cellular context. Notably, although several studies have suggested that cPHAs may be degraded by nonspecific esterase or depolymerase activities, the specific enzymes responsible for cPHA or oligo PHA depolymerization have not yet been identified[8].

Our study represents a significant advance for two reasons. First, we demonstrate that animals can enzymatically degrade microbial sPHAs, a capacity previously thought to be restricted to bacteria, fungi, and a limited number of protists[9]. Second, we identify and functionally validate a specific enzyme mediating this activity. Our findings therefore reveal a previously unrecognized enzymatic capacity in animals.

5. Based on the earlier findings and that it has been previously shown that human produce PHAs and degrade them, the current title is misleading

Revised accordingly.

6. Interestingly, and as the authors state correctly, no functional characterization of an animal-derived PHA 'depolymerase' as a homologue of bacterial PhaZ protein has been published until today. However, they are annotated as such in various eukaryotic and animal genomes.

We agree and have clarified this distinction in the revised text (lines 71-73). While several eukaryotic and animal genes are annotated as putative PHA depolymerases (PHB depolymerase-like enzymes) in public databases, none have been functionally characterized. Our study

therefore provides the first experimental evidence confirming that these annotated homologs are indeed active PHADs capable of degrading microbial sPHAs.

7. A: Further the authors do not consider that Olavius algarvensis most likely produces polyhydroxyalkanoates itself. Their claim that the identified enzyme acts mainly on bacterial PHAs is speculative and not based on data presented in this study. The enzyme could have a role linked to degradation of endogenous PHB produced by Olavius algarvensis

We agree that *O. algarvensis* may produce low molecular weight cPHAs and oligo PHAs, and we do not exclude that its PHAD could also act on such endogenous polymers. However, the reviewer's comment does not contradict our central conclusion. Our heterologous expression assays directly demonstrate that the *O. algarvensis* PHAD efficiently degrades microbial sPHAs, establishing - based on experimental evidence - that animal PHADs possess the enzymatic capacity to act on microbial sPHAs. This activity has previously been documented only in bacteria and fungi. While a potential additional role in host-derived PHAs remains an interesting possibility, it is not required to explain the observed activity and is therefore not addressed further in this study.

B. It is further likely that the Olavius algarvensis also carries a gene similar 3-hydroxy-3-methylglutaryl-CoA lyase involved in depolymerization of PHAs as well.

We respectfully disagree. While *O. algarvensis* expresses both 3-hydroxy-3-methylglutaryl-CoA (HMG-CoA) lyase and synthase, these enzymes are components of the ketone body pathway, catalyzing the conversion of acetoacetyl-CoA and acetyl-CoA to HMG-CoA and subsequently to acetoacetate[10-12]. Their presence is therefore not indicative of PHA degradation or synthesis. To our knowledge, no animal enzymes have been experimentally shown to degrade cPHAs or oligo-PHAs[8], and we found no evidence that *O. algarvensis* possesses such genes.

8. The biochemical and structural characterization lacks solid data. Halo formation on agar plates containing emulsions of non-soluble substrates is a standard technology in most labs to initially verify if proteins are active or not. However, they are not considered as proper biochemical characterization. They are often inaccurate and affected by pH changes, endogenous activities of the host and thickeners of agar and type of emulsion and many other factors. The images presented in figure 14 in the supplementary material are not convincing and should not be the standard in a high profile journal. In my view a careful characterization would require enzyme assays and kinetics providing data measured under standard and reproducible conditions. Specific activities would be mandatory. With this respect it would be mandatory to show what are the primary degradation products. Is ß-OH butyrate released or are dimers and/ or oligomers released? Is the enzyme an exoenzyme or endo-acting? These are all questions that need to be answered before claims can be made that the current enzyme is a true PHA-depolymerase.

   Also, it would be good to know what the optimum temperature of the enzyme is and if cofactors are required. A melting point analysis and other additional biochemical structure data should be included. These data should be placed in context and benchmarked against data of known and well characterized enzymes.

The observation that purified enzyme needed 24 hours to produce a halo indicates already that there is most likely only a very weak activity, and essential cofactors are perhaps missing.

Please, be aware that nature has invented some very generic hydrolases that are able to hydrolyze multiple substrates some of these enzymes turn over more than 80 substrates.

With this respect it would be mandatory to show what are the primary degradation products. Only then the reader can verify that the enzyme activity is measured are not promiscuous side activities of a generic alpha/beta-hydrolase. It is noteworthy that cutinases and some lipases also act on PHB. https://www.ncbi.nlm.nih.gov/pmc/articles/PMC1287645/.

We agree, and as described in our replies above we now provide new experimental data demonstrating that animal PHADs degrade sPHAs to 3-hydroxybutyrate, consistent with the activity of microbial PHADs. In addition, our phylogenetic analyses exclude that the animal PHADs described here are cutinases or lipases (see our response to Reviewer 2, Comment #8, and Supplementary Figures10 & 11).

We agree that detailed biochemical characterization (e.g. temperature optima, cofactors, and kinetic parameters) would be valuable. However, such analyses are not required to support the conclusions of this study. The central advance of our work is the identification and functional validation of a previously unrecognized animal PHAD and the demonstration that animal PHADs can degrade microbial sPHAs, a capability previously thought to be restricted to bacteria and fungi. By showing enzymatic activity, our data establish the core functional framework required to support this conclusion. More detailed biochemical characterization would represent a logical and valuable next step for future studies building on this work, but is not essential for establishing the central conclusion of the present study.

9. A: The transcriptome data leave the question open if full length gene transcripts were observed and what the overall coverage was. In fact it would be appropriate to include a few metadata on the overall reads and also on the full-length genes that were hit. Simply giving a download link it's not sufficient

We agree and now provide additional details regarding the transcriptomic data in our manuscript. Supplementary Table 2 includes metadata for each of the sequencing libraries used in our study, including links to the raw data uploaded to ENA, and overall coverage and completeness of the transcripts (99-686 amino acids; completeness 31% to 100%; coverage 21% to 100%).

B: This reviewer could not find any amino acid sequence for the predicted de-polymerase. Therefore, it is very difficult to verify any of the claims with respect to the structure, active side and homology to other known organisms. It would be mandatory to provide the complete protein sequence to allow a careful data analysis.

We have included the PHAD sequences from the gutless oligochaetes and their symbionts in Supplementary File 2. All other animal PHADs were recovered from online databases and the corresponding accession numbers are listed in Supplementary Table 6.

10. The authors further stated that the gene was found in the genome. Unfortunately, no information is provided whether the symbiont carries this gene somewhere on the

chromosome (which one?) or if it is part of a mitochondrial genome. This information would be essential to judge on the phylogenetic origin of the respective gene.

We agree that determining the genomic location of the host PHAD would be informative. In our manuscript, we show that the symbiont *T. algarvensis* encodes a *phaZ* gene in its genome and that this enzyme does not cluster phylogenetically with the *O. algarvensis* PHAD (Figure 1, lines 111-117). Because a complete *O.* algarvensis genome is not yet available, we cannot currently determine the chromosomal context of the host PHAD or assess a potential mitochondrial localization. However, we note that establishing the genomic location is not required to infer phylogenetic origin, which can be robustly assessed from sequence-based phylogenetic analyses, as shown in comparable studies[13-15].

11. Figure 1 and elsewhere: the legend should clearly state that the structure is a predicted structure and also the active side is a predicted active side no experimental data have been presented to verify any of the functions. The same is true for the predicted signal peptide.

We agree and have revised accordingly.

12. With only a 10.2% identity to the best characterized bacterial and 31% to the best characterized eukaryotic enzymes the structure predictions should be taken with care. The section on the size and volume of the active site is highly speculative and lacks biochemical data.

We agree and revised accordingly, described in our responses to Reviewer #2, Comments #2 and #8.

13. Supplementary figure 3 shows that mRNA and a can be detected, and the gene is most likely expressed. It would be essential to provide protein data. Thus, can the activity be measured in vivo or at least in an in vitro model on a protein level?

We agree and have now added new proteomic data to address this point. The *O. algarvensis* metaproteome revealed peptides matching the host PHAD (but not the symbiont PHAD) in the trunk region, which harbors the sPHA producing *Thiosymbion* symbiont. No corresponding peptides were detected in the worm's tip, which lack this symbiont. These newly added results indicate that PHAD activity occurs specifically at the site of PHA storage. These results are now presented in Supplementary Table 3, and lines 149-150 have been revised accordingly.

Revised Main Figures:

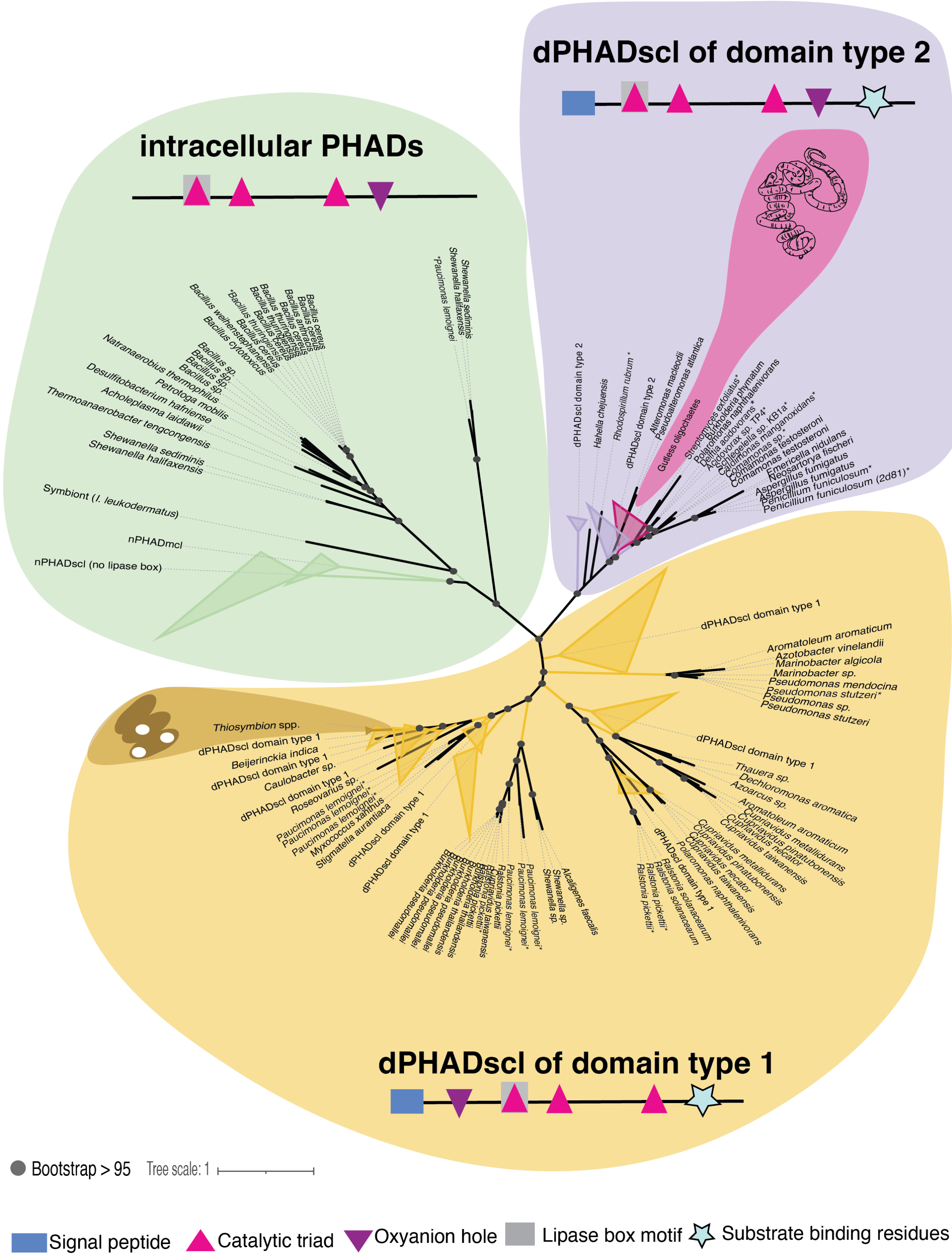

Figure 1 | The PHADs of *O. algarvensis* and its symbiont *Thiosymbion algarvensis* are phylogenetically distinct. The two enzymes fall into two different clades of extracellular PHADs (dPHADs). Comparative analyses of PHADs from gutless oligochaetes, their symbionts and the PHAD engineering database (DED) revealed three major clades: domain type 1 dPHADs (yellow), domain type 2 dPHADs (purple), and intracellular PHADs (green). Extracellular PHADs degrade denatured sPHAs outside of the cell, and the oxyanion hole (purple triangle) is positioned either before (domain type 1) or after (domain type 2) the catalytic triad (pink triangle). Intracellular PHADs degrade native sPHAs inside the cell, lack a signal peptide (blue box), and possess an undescribed substrate-binding site (cyan star). *O. algarvensis* PHADs group with domain type 2 dPHADs (pink clade), while those of their symbiont group with domain type 1 dPHADs (dark yellow clade). Experimentally validated PHAD sequences are marked with an asterisk (*). The tree was calculated using IQ-TREE with ultrafast bootstrap support (1,000 replicates) and was based on an amino acid alignment.

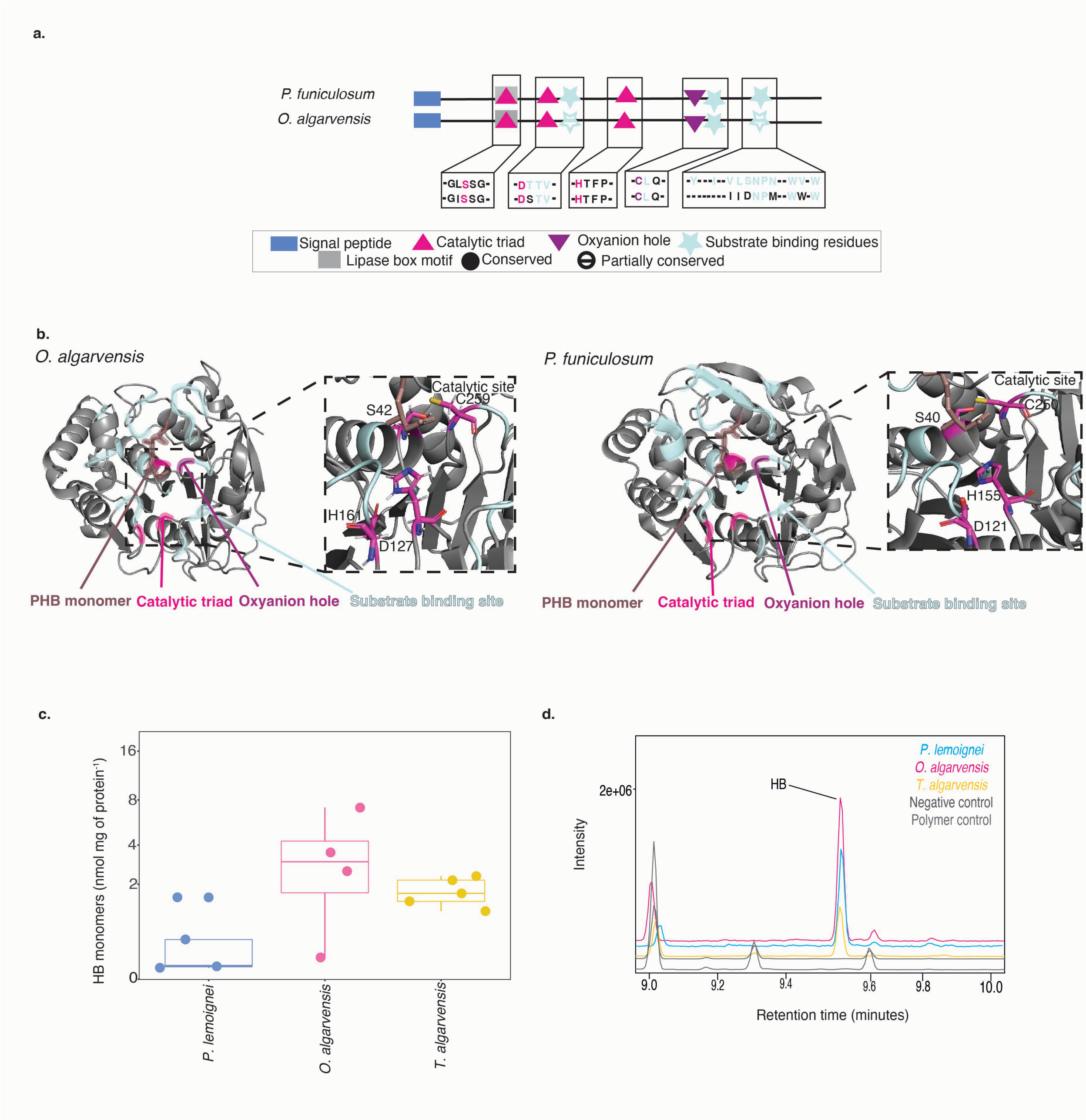
a.
P. funiculosum
O. algarvensis
Signal peptide
Catalytic triad
Oxyanion hole
Substrate binding residues
Lipase box motif
Conserved
Partially conserved
b.
O. algarvensis
P. funiculosum
Catalytic site
S42
C259
H161
D127
S40
H155
D121
PHB monomer
Catalytic triad
Oxyanion hole
Substrate binding site
c.
HB monomers (nmol mg of protein-1)
P. lemoignei
O. algarvensis
T. algarvensis
d.
P. lemoignei
O. algarvensis
T. algarvensis
Negative control
Polymer control
HB
Intensity
2e+06
Retention time (minutes)

Figure 2 | *O. algarvensis* PHAD degrades PHB into hydroxybutyrate (HB) monomers. a, A comparison between the primary structures of the *phaZ* gene between *O. algarvensis* and the fungus *P. funiculosum*. The *O. algarvensis* protein spans 333 amino acids, while the *P. funiculosum* PHAD has 318 amino acids. The signal peptide spanning the first 20 amino acids (blue square), the catalytic triad including the catalytic serine residue (S42, D127, H167; pink triangles) located in the lipase box (gray box) and the C259 residue of oxyanion hole (purple triangle) are fully conserved between the two enzymes (filled symbols). The substrate binding domain is only partially conserved (partially filled light blue star) and shares 29.2% identity to that of the fungal homolog. All other residues in the primary structure are represented by the black line. **b,** The AlphaFold2 predicted tertiary structure of the *O. algarvensis* PHAD (left) and the crystal structure of the homolog from *P. funiculosum* (right) show strong similarity based on the superposition of the two enzymes using PyMol (PDB 2d81; RMSD 0.773 Å; RMSD catalytic domain 0.045-0.822 Å; RMSD substrate binding domain 0.471 Å). The insets show the catalytic triad (dark pink) that surrounds the PHB (brown) based on the model's prediction (oxyanion hole in purple, substrate-binding domain in light cyan). **c,d,** GC-MS analyses of PHAD assays using PHB (n=4). **c,** Boxplots show the concentration of HB monomers resulting from enzymatic degradation of PHB. The *O. algarvensis* PHAD (pink) produces more HB monomers than its symbiont (*T. algarvensis* yellow) and the bacterial PHAD used as a positive control (*P. lemoignei,* blue). **d,** Total ion chromatograms show the relative peak heights of the HB monomers in representative assays (9.56 min). No HB was detected in assays using a heat inactivated PHAD (dark gray) or controls without the polymers; light gray, Supplementary Figure 3).

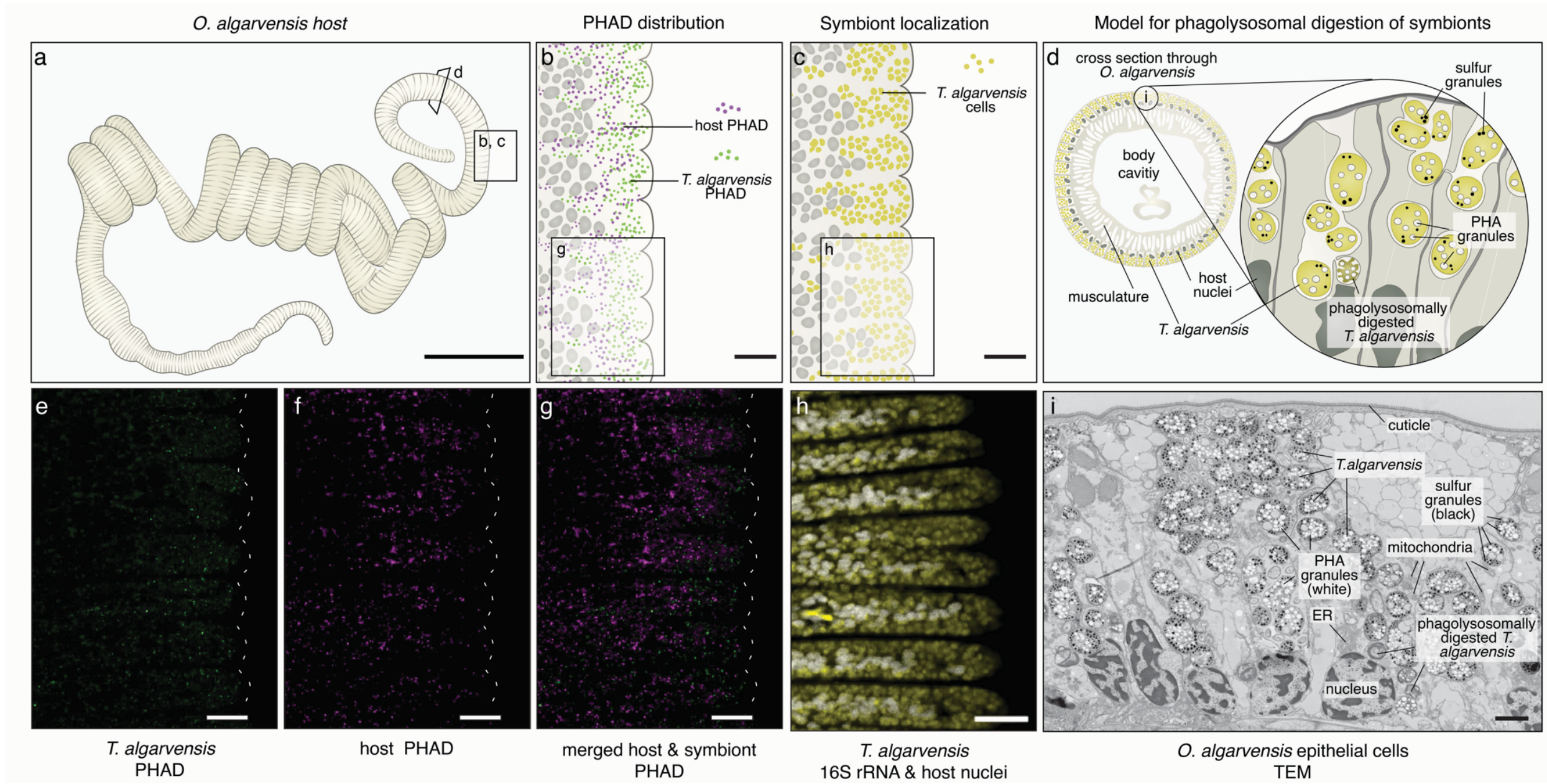

Figure 3 | The PHAD of *O. algarvensis* is expressed in its epidermal cells where symbionts are digested through phagolysosomal activity. **a,** Schematic drawing of *O. algarvensis* highlighting the focal planes shown in panels **b-d**. **b,** Schematic overview of HCR-FISH showing PHAD transcripts from the symbiont *T. algarvensis* (green; **e**) and its host (pink; **f**). Symbiont and host signals do not overlap, as shown in the merged image (**g**). **c**, The symbionts (yellow), visualized by whole mount 16S rRNA FISH (**h**), are located directly beneath the host's cuticle (gammaproteobacterial probe GAM42a; host nuclei stained with DAPI in grey). **d**, Schematic of host epithelial cells showing symbionts and their phagolysosomal digestion based on the TEM image in (**i**). The symbionts contain two types of granules: large sulfur vesicles (light grey; S^0 212.7 nmol per average worm) and smaller, more numerous sPHA granules (black to white; 0.7 µmol carbon per worm). Scale bars: a = 6 mm, b-c, e-h = 20 µm, d,i = 2 µm. See Supplementary Figure 5-7 for imaging controls and focal plane analyses.

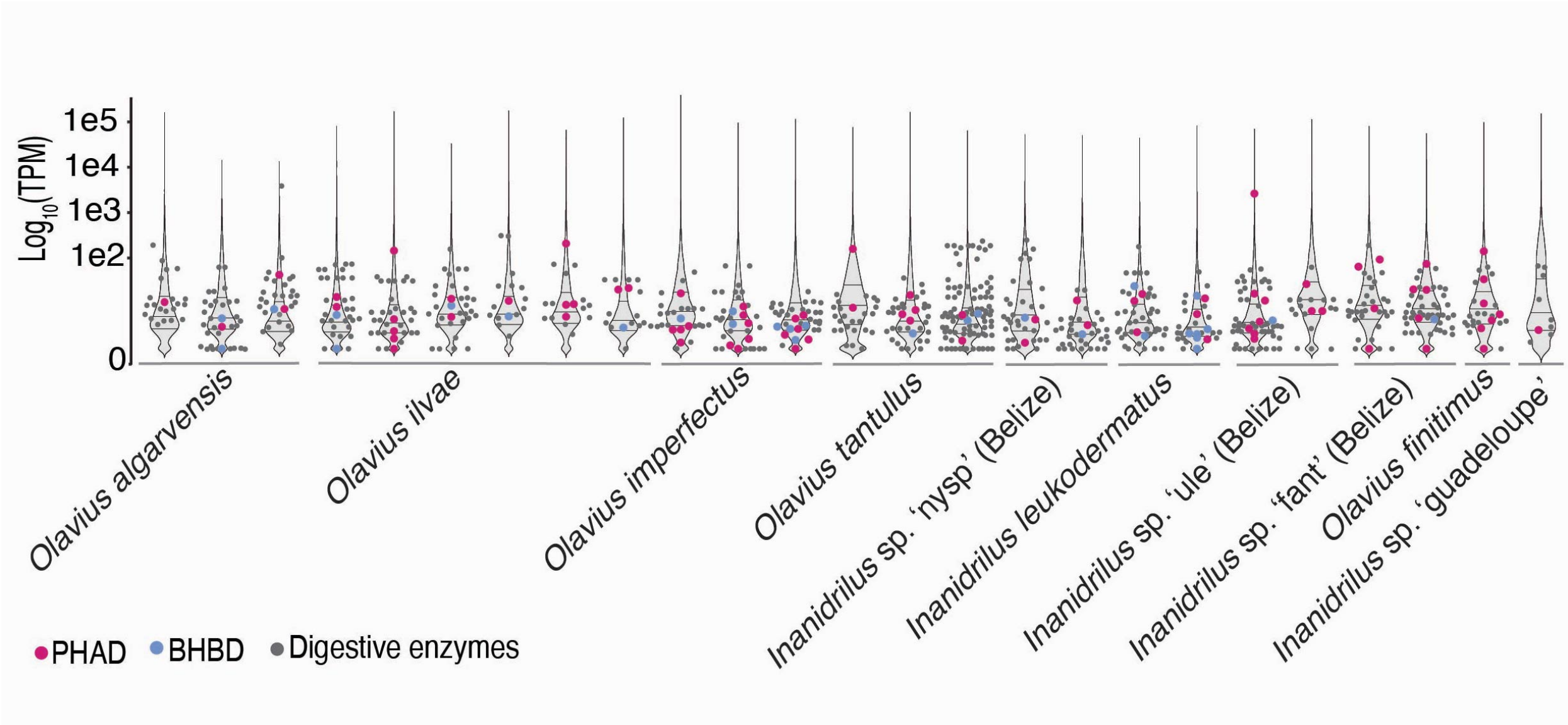

Figure 4 | Gutless oligochaetes express enzymes required for sPHA degradation. We quantified PHAD and BHBD transcript abundances in the ten species of the genus *Olavius* and *Inanidrilus* for which we had host-enriched transcriptomes (the transcriptomes from the other five gutless oligochaete species with PHADs were from total RNA (host and symbiont), and could therefore not be used to quantify host transcripts). Violin plots show assembled host transcriptomes of single individuals from ten species (shown as normalized transcription (log10(TPM)) per individual worm), with PHAD transcripts shown in pink and BHBD transcripts in blue. PHAD and BHBD transcript abundances are similar to those of other digestive enzymes (gray points), with BHBD detected in eight and PHAD in all ten host species examined. To estimate transcript abundances, we assembled one reference assembly per species and mapped the raw reads of the libraries from single individuals to the reference assembly, to obtain normalized transcript abundances.

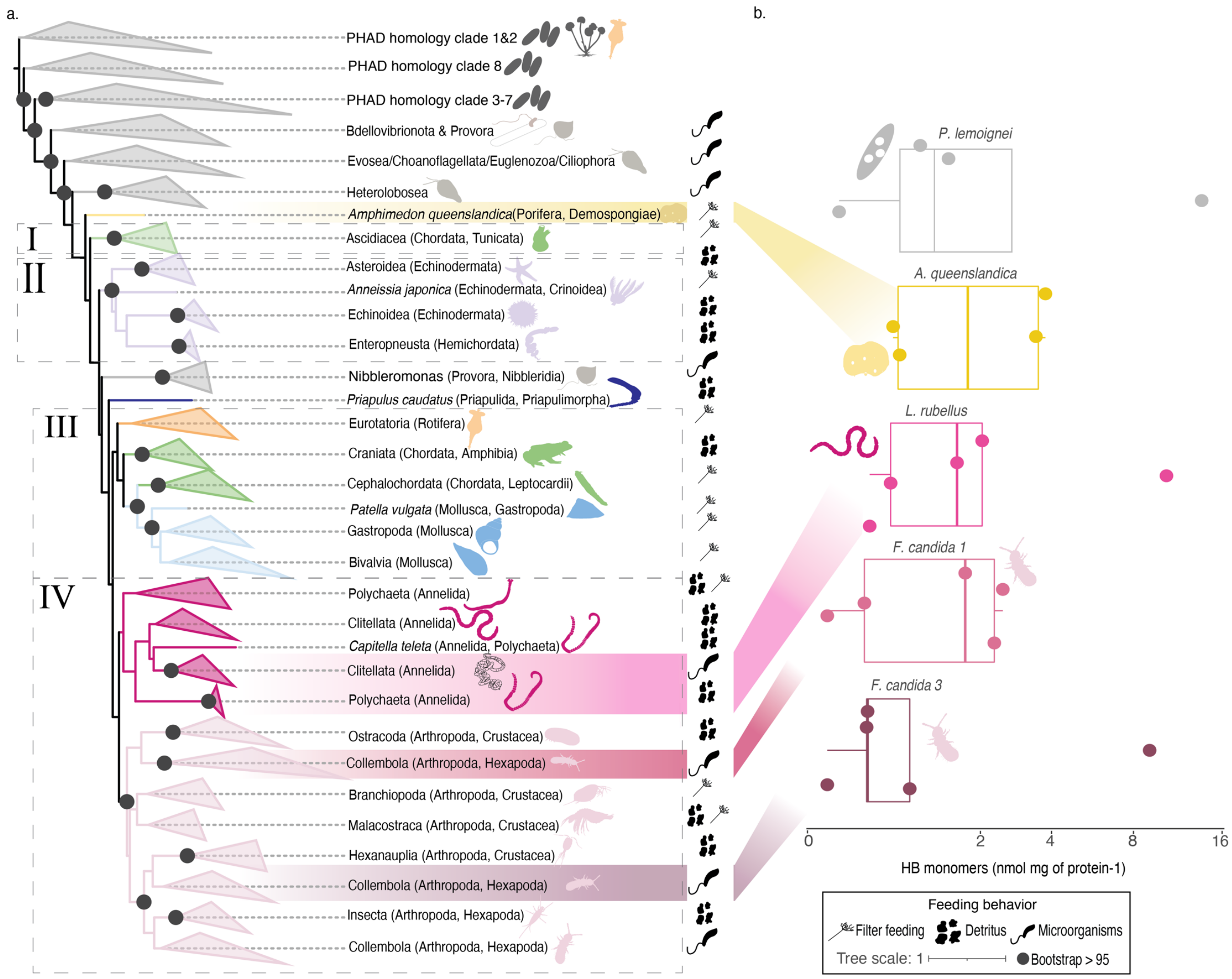

Figure 5 | PHADs are widespread across animals. **a,** Maximum-likelihood tree of 208 PHAD sequences from animals spanning nine phyla, calculated using IQ-TREE with ultrafast bootstrap support (1,000 replicates). The tree includes representatives from Porifera (yellow), Chordata (green), Hemichordata and Echinodermata (purple), Priapulida (dark blue), Rotifera (orange), Mollusca (blue), Annelida (pink), and Arthropoda (light pink); gutless oligochaete PHADs are highlighted in bright pink. Reconstruction of the feeding ecology of PHAD-bearing animals and protists indicates that these organisms may acquire sPHAs by consuming sPHA-producing microorganisms directly or indirectly through soil, sediment, or detritus. Icons denote feeding behavior, and color ranges correspond to the phylum of origin. Colored bars mark sequences that showed PHAD activity in our assays. **b,** Boxplots show the concentrations of HB monomers, measured by GC-MS, produced in PHAD enzyme assays using PHB. All tested enzymes, including those from the sponge *A. queenslandica* (yellow), the springtail *F. candida* (light pink, two homologs), and the earthworm *L. rubellus* (pink), generated measurable HB monomers. No HB monomers were detected in the non-sense vector negative control or polymer blank samples (see Supplementary Figure 12 & 13; assays done on PHB/PHV see Supplementary Figure 14).

Revised Supplementary Figures:

a.

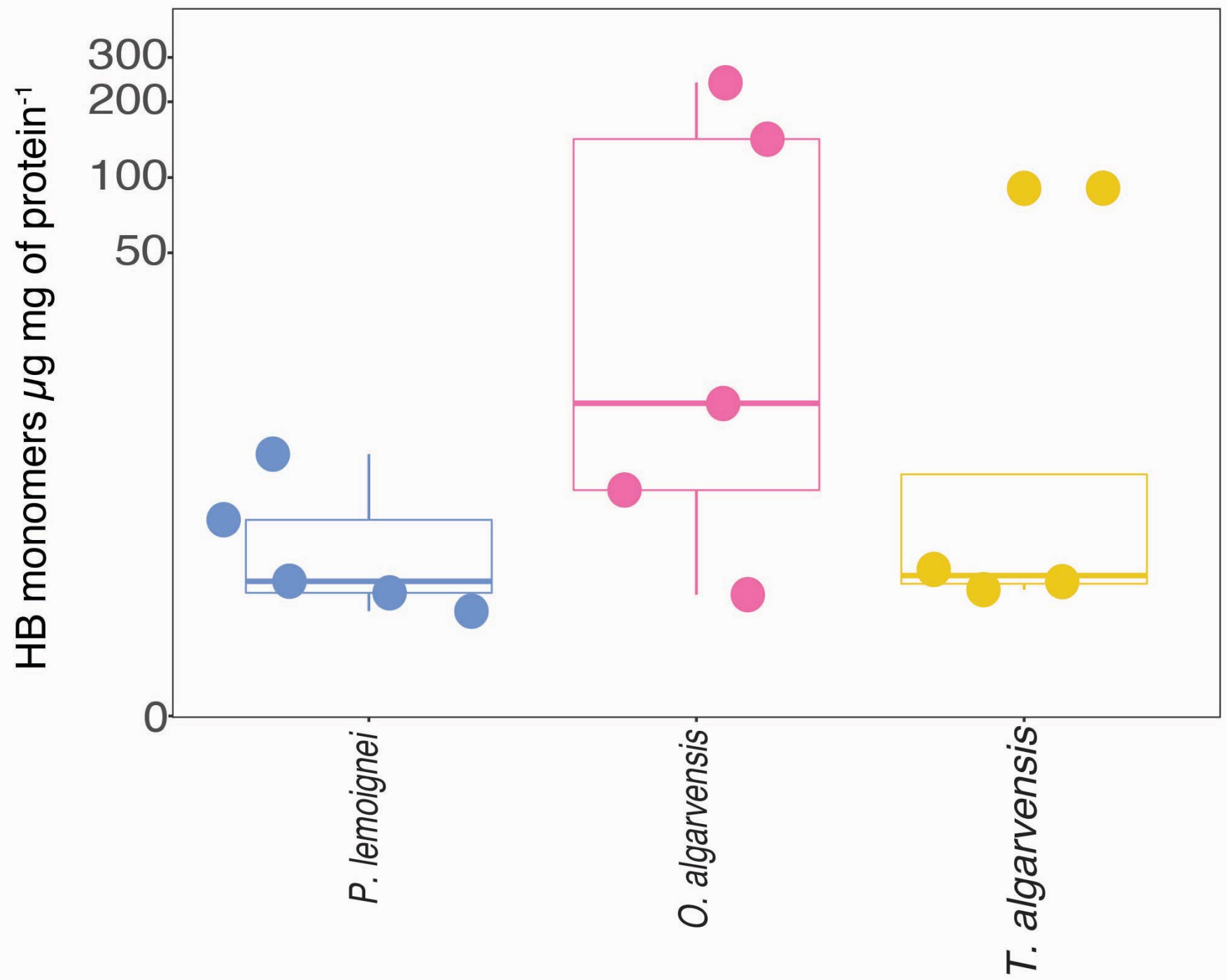

b.

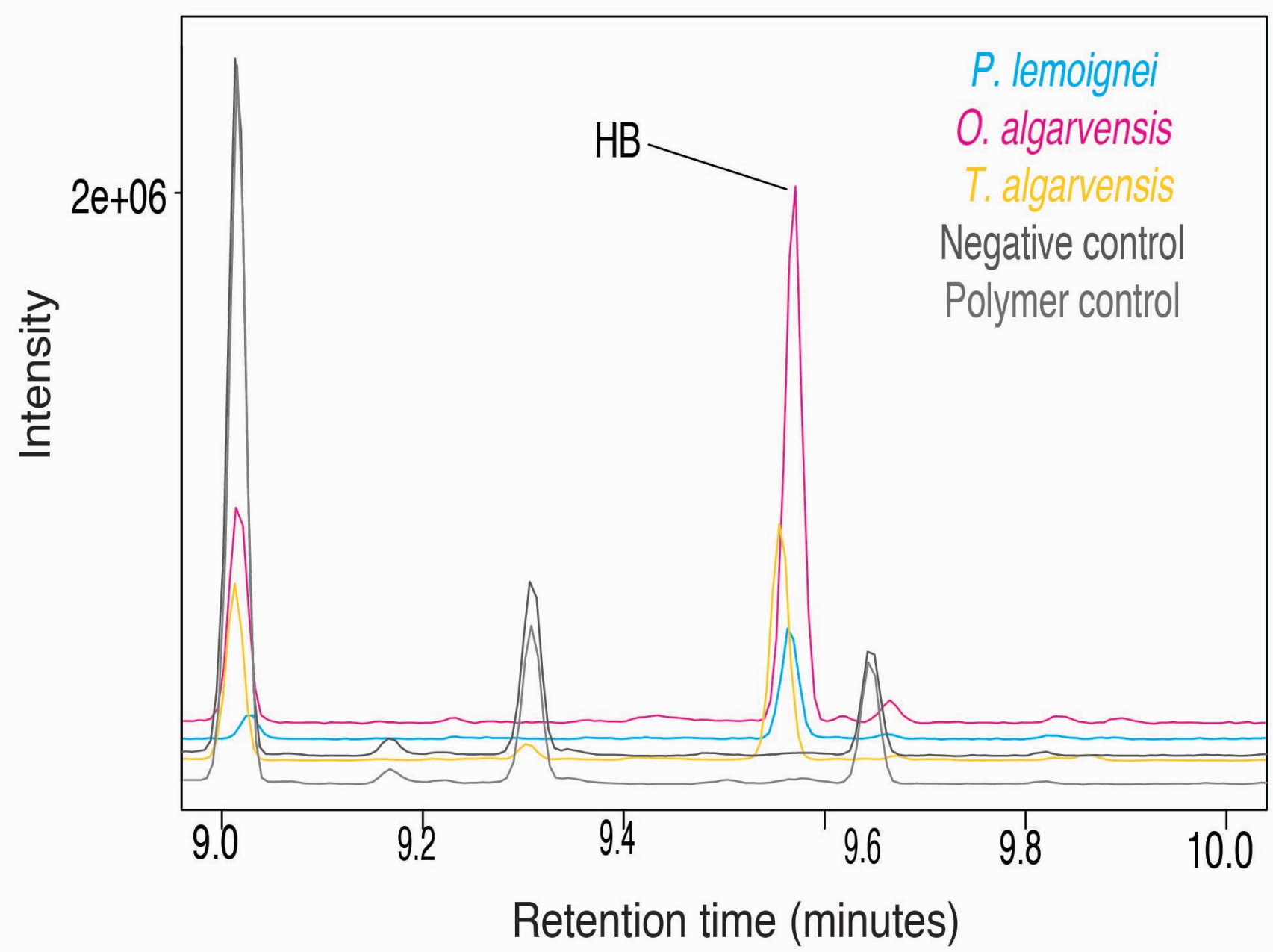

Supplementary Figure 2 | *O. algarvensis* PHAD degrades PHB/PHV into hydroxybutyrate (HB) monomers. a, b, GC-MS analyses of PHAD assays using PHB/PHV (n=5). **a,** Boxplots show the concentration of HB monomers as a function of enzyme activity. The *O. algarvensis* PHAD (pink) produced more HB monomers than its symbiont (*T. algarvensis*, yellow) and the bacterial PHAD used as a positive control (*P. lemoignei,* blue). **b,** Total ion chromatograms show the relative peak heights of the HB monomers in representative assays (9.56 min). No HB was detected in assays using a heat inactivated PHAD (dark gray) or the controls without the enzyme (light gray, Supplementary Figure 3). Hydroxyvalerate (HV) monomers were not detected because the PHB/PHV substrate contained only 3% HV, which was below the detection limit of this assay.

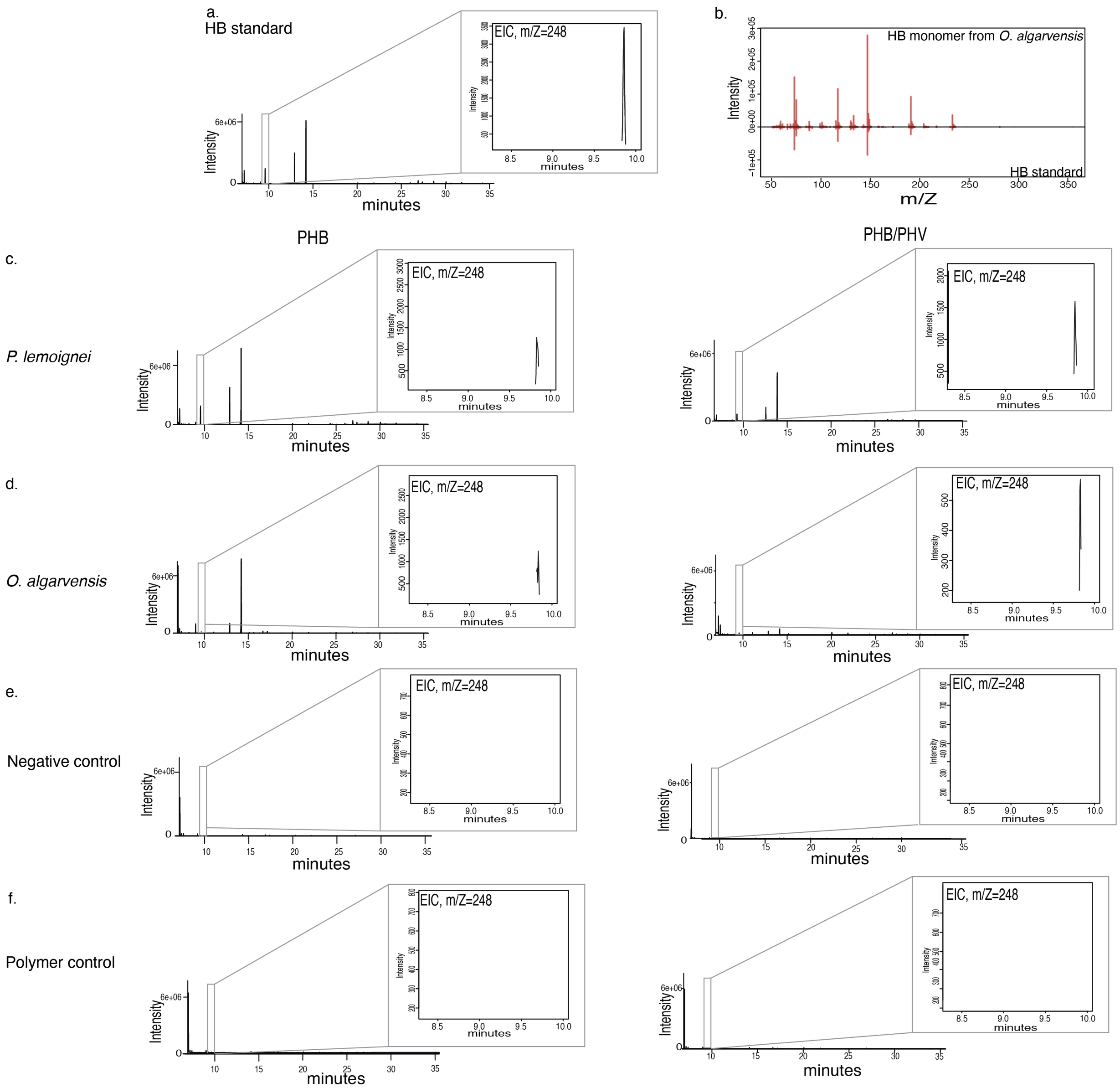

Supplementary Figure 3 | GC-MS identification of HB monomers from PHAD assays of PHB and PHB/PHV. a, Total ion chromatogram (TIC) of the HB standard showing a retention time of 9.56 min. The inset shows the extracted ion chromatograph (EIC) of the HB standard (m/z 248). **b,** Mass spectra demonstrating that HB produced in spot assays with heterologously expressed *O. algarvensis* PHAD match the mass spectra of the HB standard**. c, d,** TICs of PHAD assays using PHB and PHB/PHV for **c,** *P. lemoignei* and **d,** *O. algarvensis***.** Insets show EICs corresponding to the HB peak. **e, f,** No HB is detected in assays using **e,** heat inactivated PHADs or **f,** controls without the enzyme.

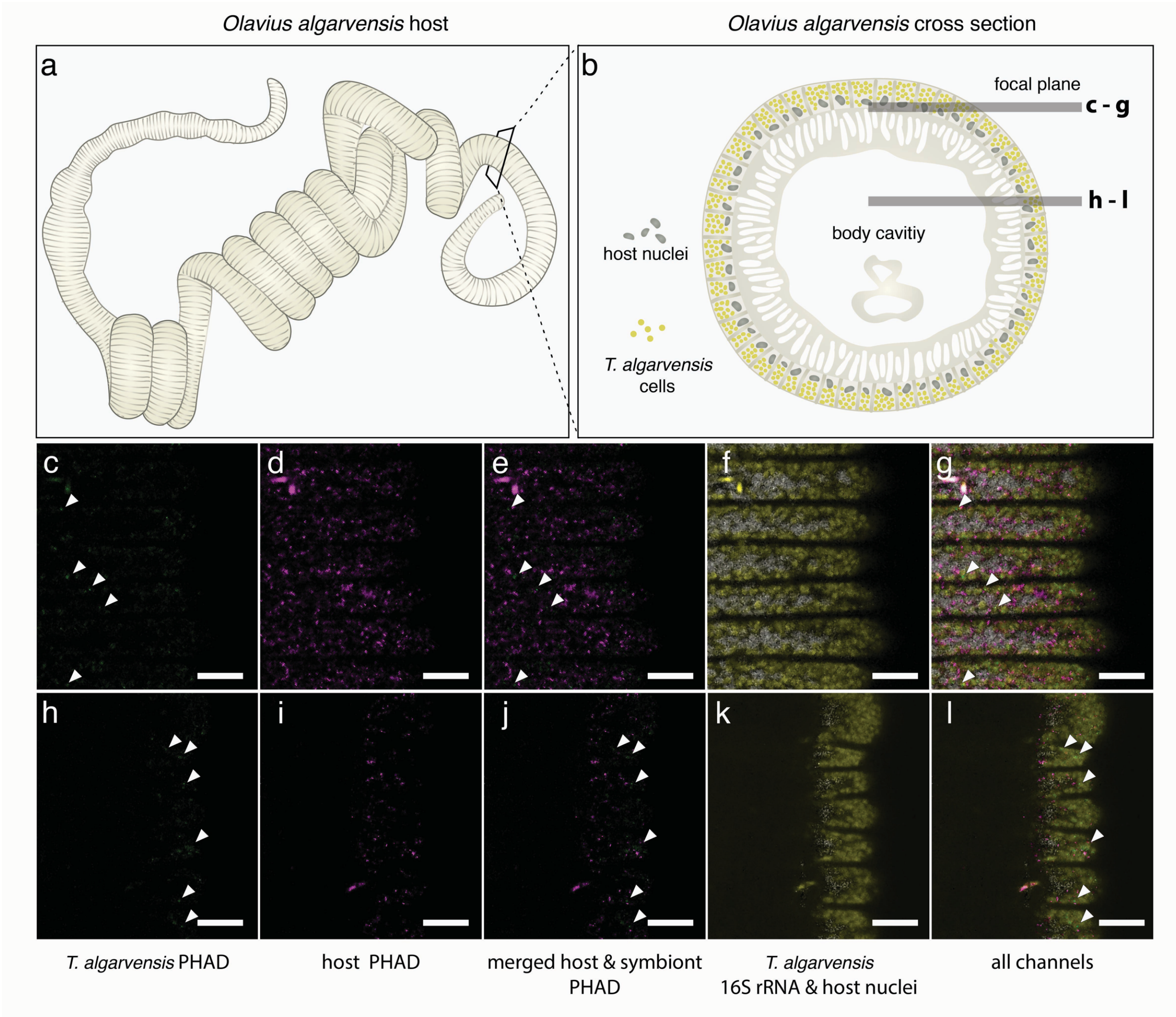

Supplementary Figure 7 | Localization of PHAD transcripts in *O. algarvensis* and its symbiont *T. algarvensis* using whole-mount HCR-FISH. **a,** Schematic drawing of the gutless oligochaete *O. algarvensis,* highlighting the region shown in **(b)**. **b**, Overview of focal planes captured in the confocal images presented in **Figure 3** and panels **c-l**. Symbionts (yellow) are located beneath the cuticle and above the host epidermal cells (gray). **c-g,** The upper focal plane captures the symbiont-rich region directly below the cuticle. **h-l,** The lower focal plane includes the symbiont region located on the right side of the images and the host coelom where no signals were observed. In **c - l**, host PHAD transcripts are shown in pink, symbiont PHAD in green, symbiont 16S rRNA in yellow, and host nuclei in grey. In the merged images (**e** and **j**), host and symbiont PHADs do not overlap, and no PHAD signals are visible in the coelom.

a.

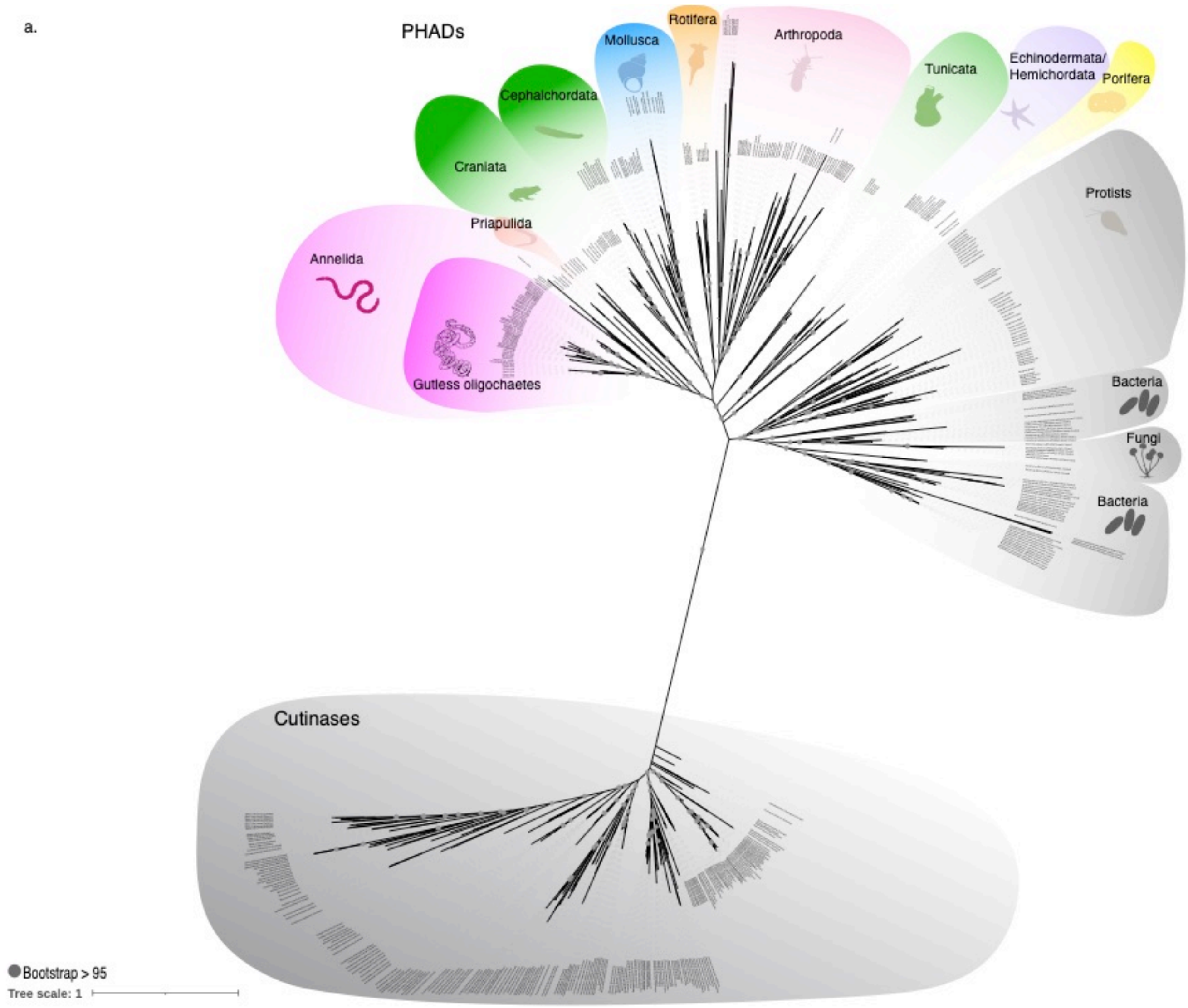
PHADs
Rotifera
Arthropoda
Mollusca
Cephalchordata
Craniata
Tunicata
Echinodermata/
Hemichordata
Porifera
Priapulida
Annelida
Gutless oligochaetes
Protists
Bacteria
Fungi
Bacteria
Cutinases
Bootstrap > 95
Tree scale: 1

b.

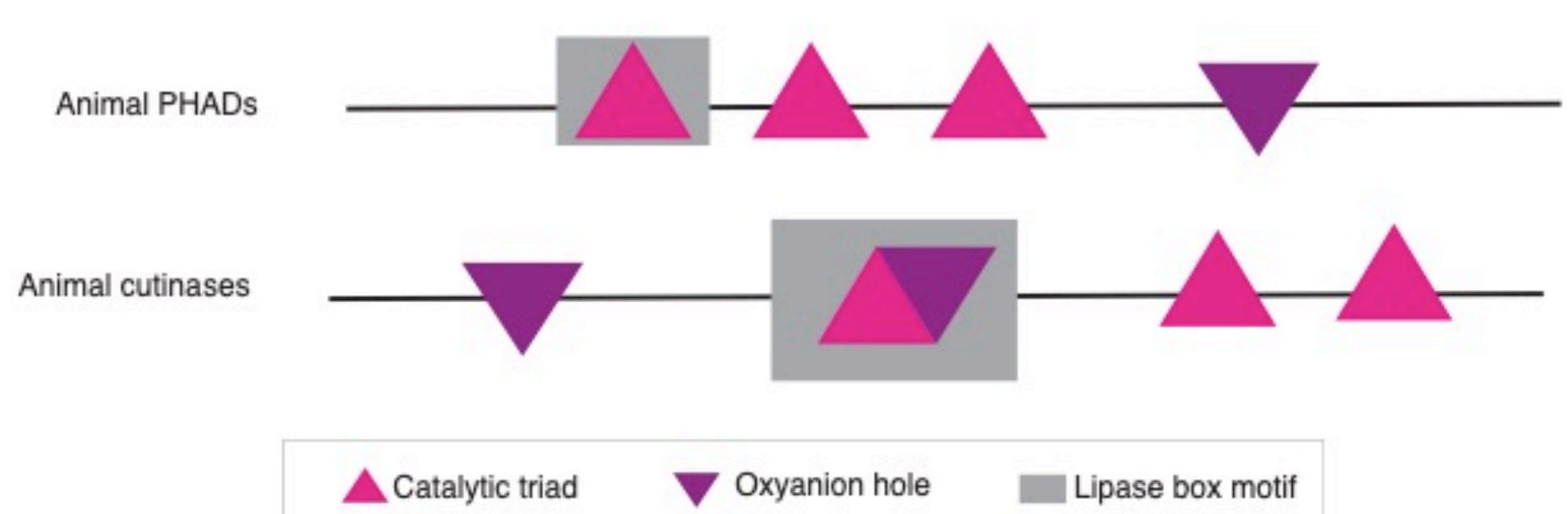
Animal PHADs
Animal cutinases
Catalytic triad
Oxyanion hole
Lipase box motif

Supplementary Figure 10 | Phylogenetic analyses distinguish animal PHADs from cutinases. a. Phylogenetic analysis shows that animal PHADs cluster with bacterial and fungal PHADs, whereas animal cutinases form a distinct and separate clade. Animal cutinase sequences were retrieved from the ESTHER database[16] and aligned together with fungal, bacterial, protist, and animal PHADs. An unrooted maximum likelihood tree was calculated from the alignment using IQ-TREE with ultrafast bootstrap support (1,000 replicates). PHADs, including those from animals (colored clades), cluster separately from the cutinase clade. **b**, Schematic representation of the primary structure alignment of animal PHADs and cutinases. The catalytic triad of cutinases and PHADs shows no conservation between cutinases and PHADs. In addition, the oxyanion hole in cutinases consists of two residues located at the N terminus and within the lipase box, whereas in animal PHADs the oxyanion hole is positioned behind the lipase box motif.

a.

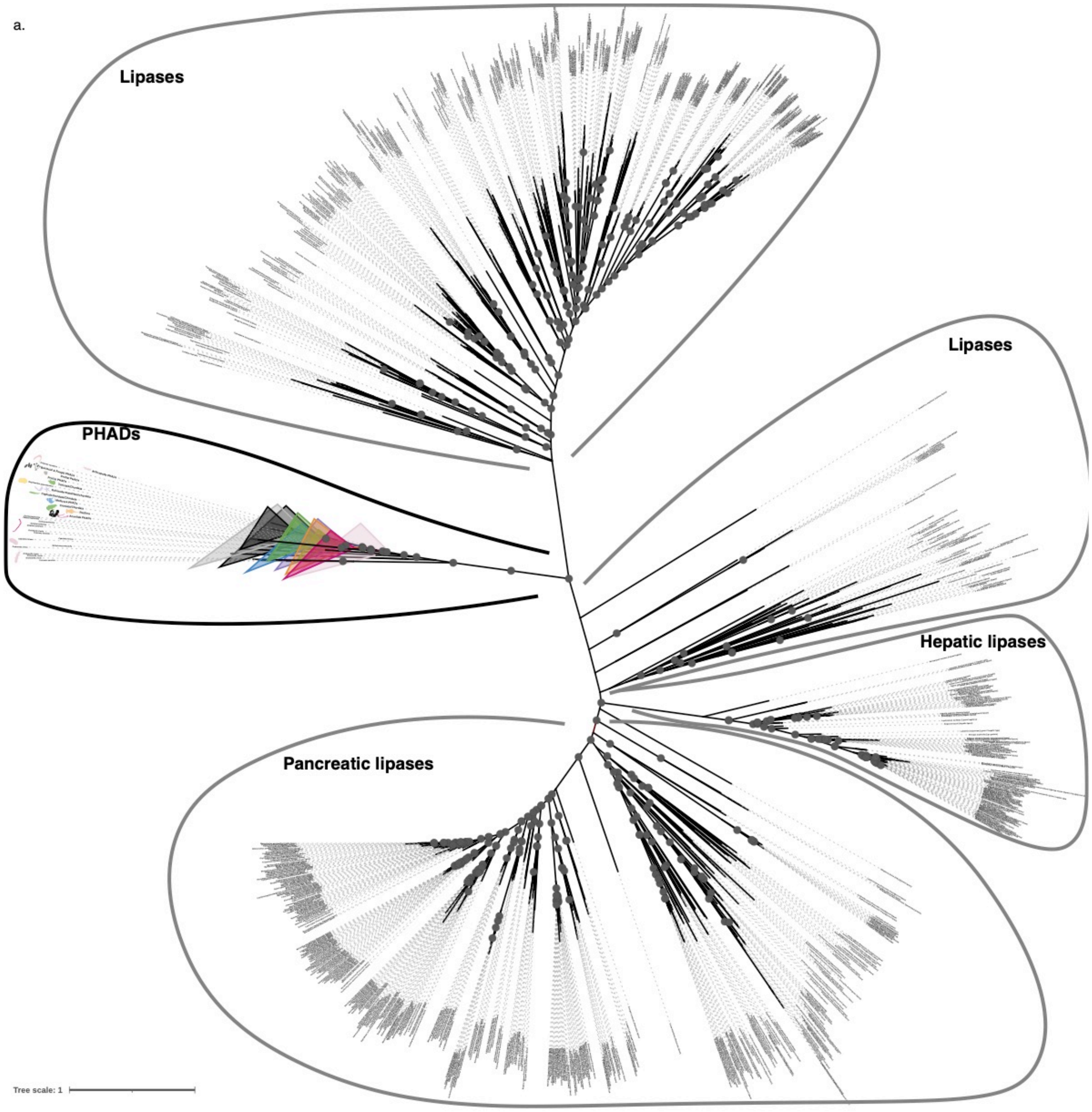

Lipases
Lipases
PHADs
Hepatic lipases
Pancreatic lipases
Tree scale: 1

b.

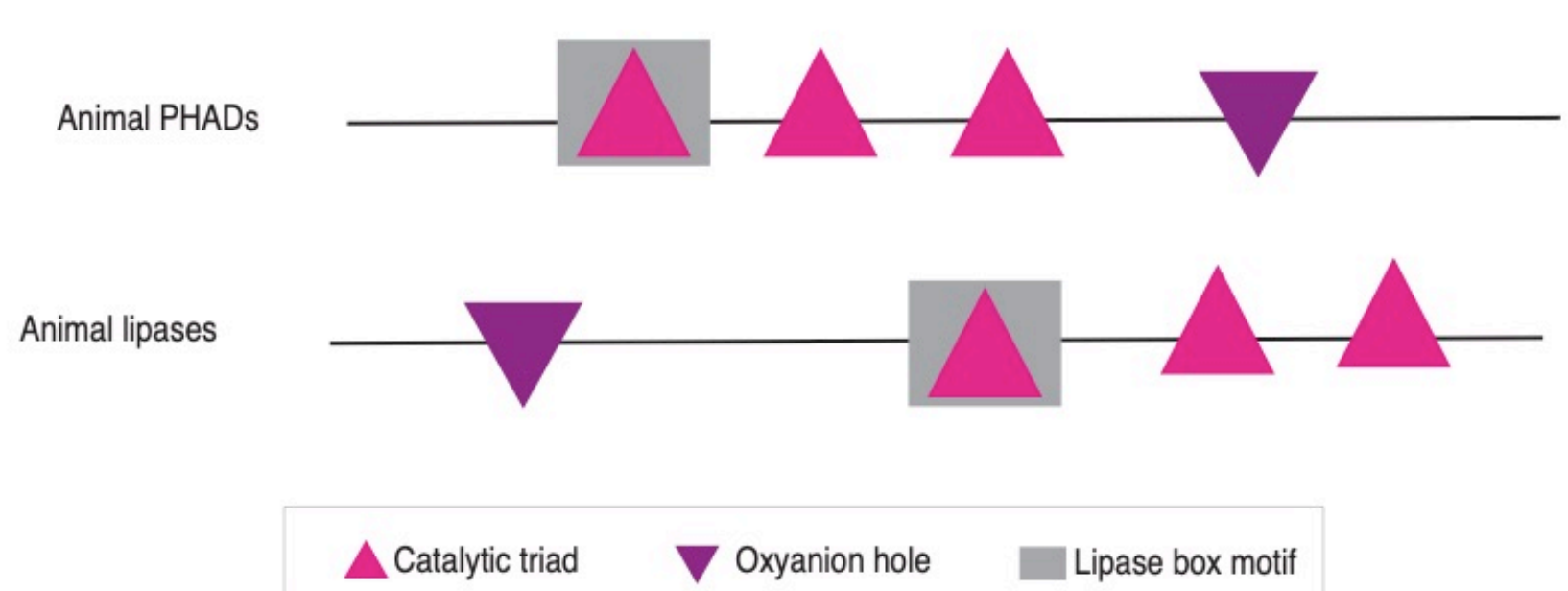

Animal PHADs
Animal lipases
Catalytic triad
Oxyanion hole
Lipase box motif

Supplementary Figure 11 | Phylogenetic analyses distinguish animal PHADs from lipases. **a,** Phylogenetic analysis shows that all animal PHADs cluster with bacterial and fungal PHADs, whereas lipases form distinct and separate clades. Animal lipase sequences were retrieved from the ESTHER database[16] and aligned together with fungal, bacterial, protist, and animal PHADs. An unrooted maximum likelihood tree was calculated from the alignment using IQ-TREE with ultrafast bootstrap support (1,000 replicates). Lipases group into four distinct clades, all clearly separated from PHADs, including animal PHADs (colored clades). **b,** Schematic representation of the primary structure alignment of animal PHADs and lipases. The catalytic triad shows no conservation between lipases and PHADs. In addition, the asparagine residue of the catalytic triad in PHADs is replaced by a glutamic acid residue in lipases, providing a clear structural distinction between the two enzyme classes.

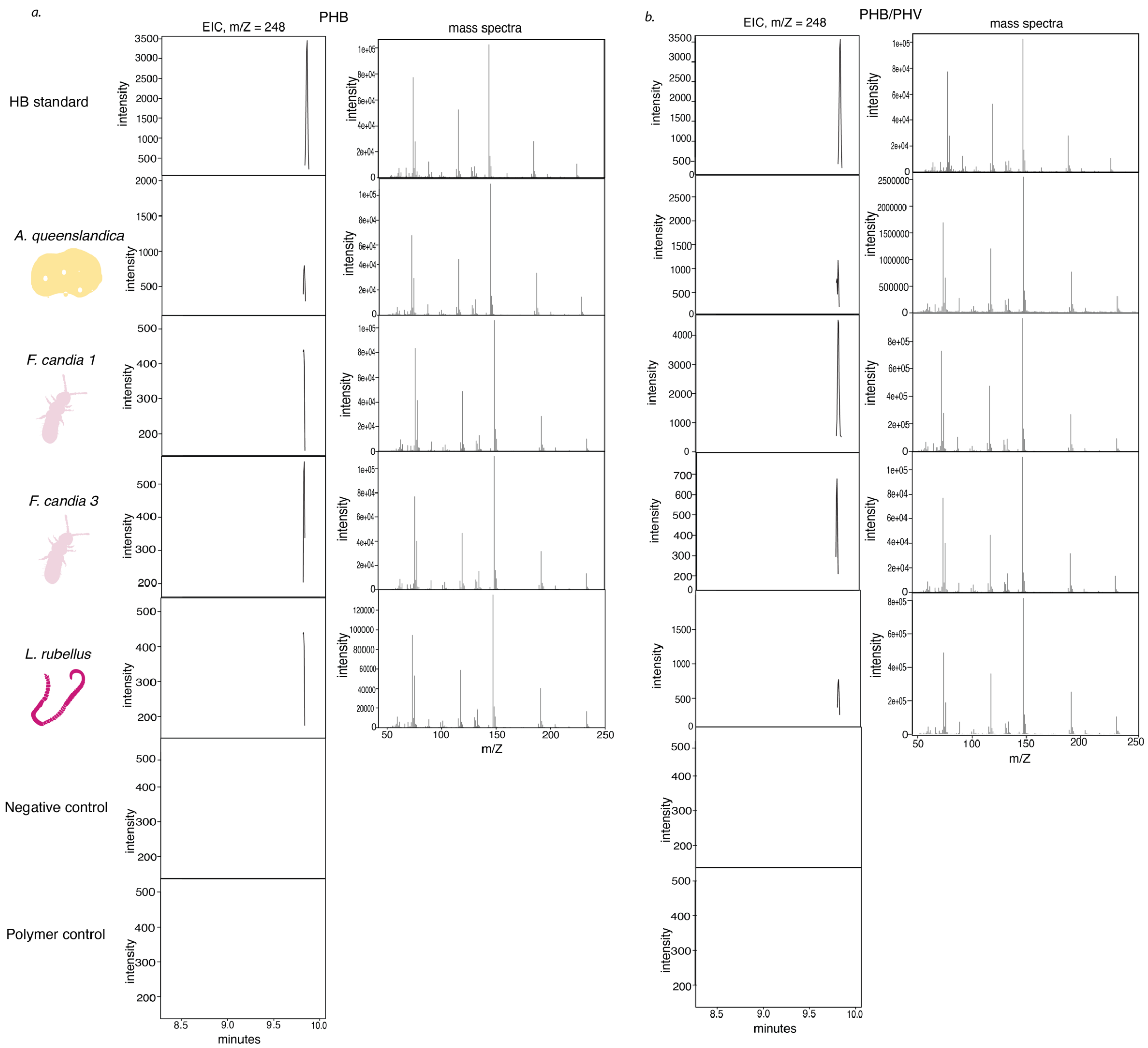

Supplementary Figure 12 | PHADs from terrestrial and marine animals degrade PHB and PHB/PHV into HB monomers. a,b, Chromatograms and corresponding mass spectra of the HB monomer peak detected in PHAD assays of the sponge *A. queenslandica*, the springtail *F. candia* (which has two PHAD homologs) and the earthworm *L. rubellus* using (**a**) PHB and (**b**) PHB/PHV. All detected HB monomers match the retention time (9.56 min) and mass spectra of the HB standard. No HB monomers were detected in assays conducted using a cell free expression with a non-sense vector (negative control) or the no-enzyme control (PHB or PHB/PHV without PHAD).

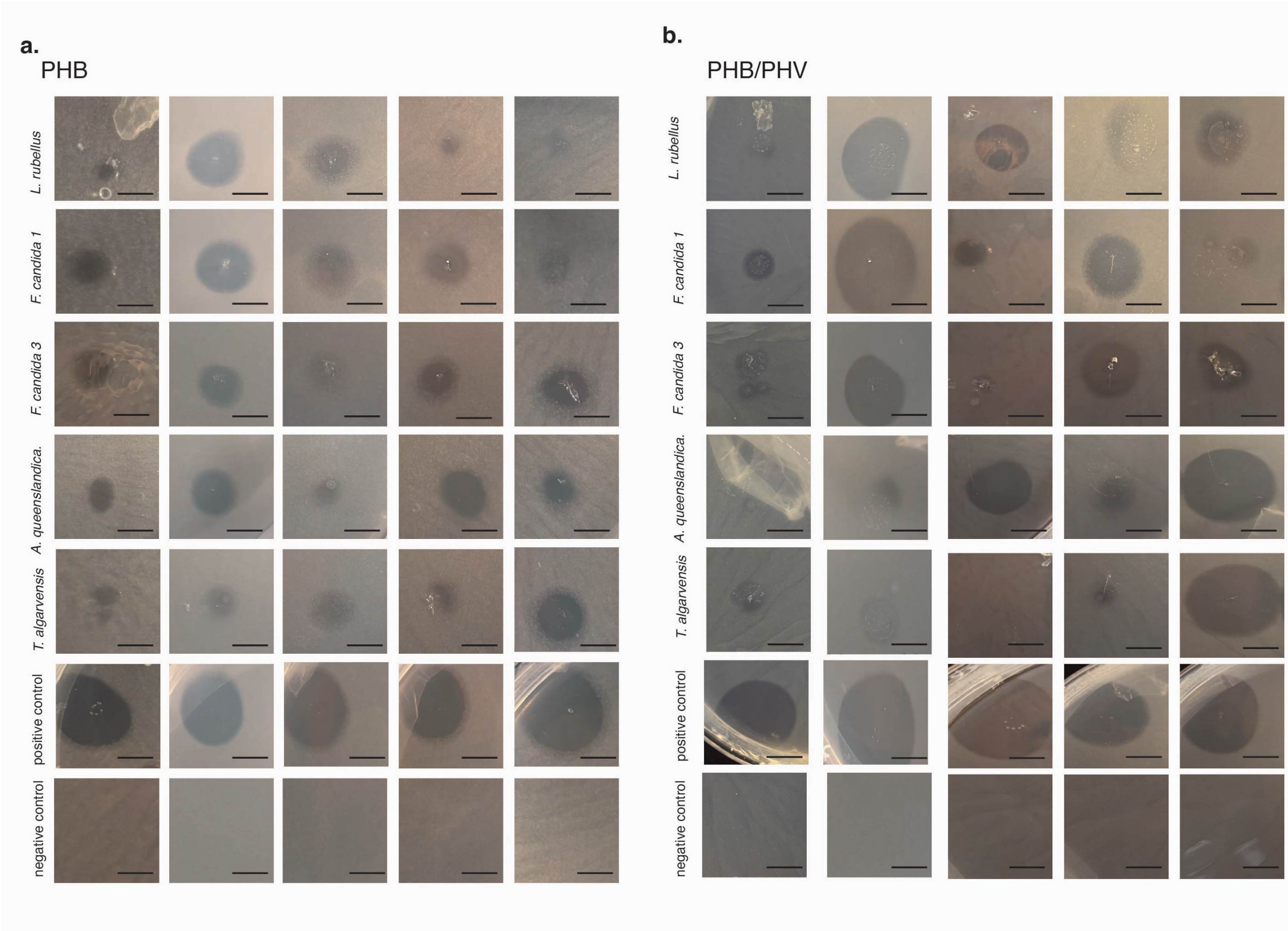

Supplementary Figure 13 | Animal PHADs degrade PHB and PHB/PHV in spot assays. Enzyme spot assays (n=5) of four animal PHADs and the symbiont *T. algarvensis* PHAD on **a**, PHB and **b,** PHB/PHV assay plates. Expression of a nonsense vector produces no clearance zone and thus indicates no enzymatic activity (negative control).

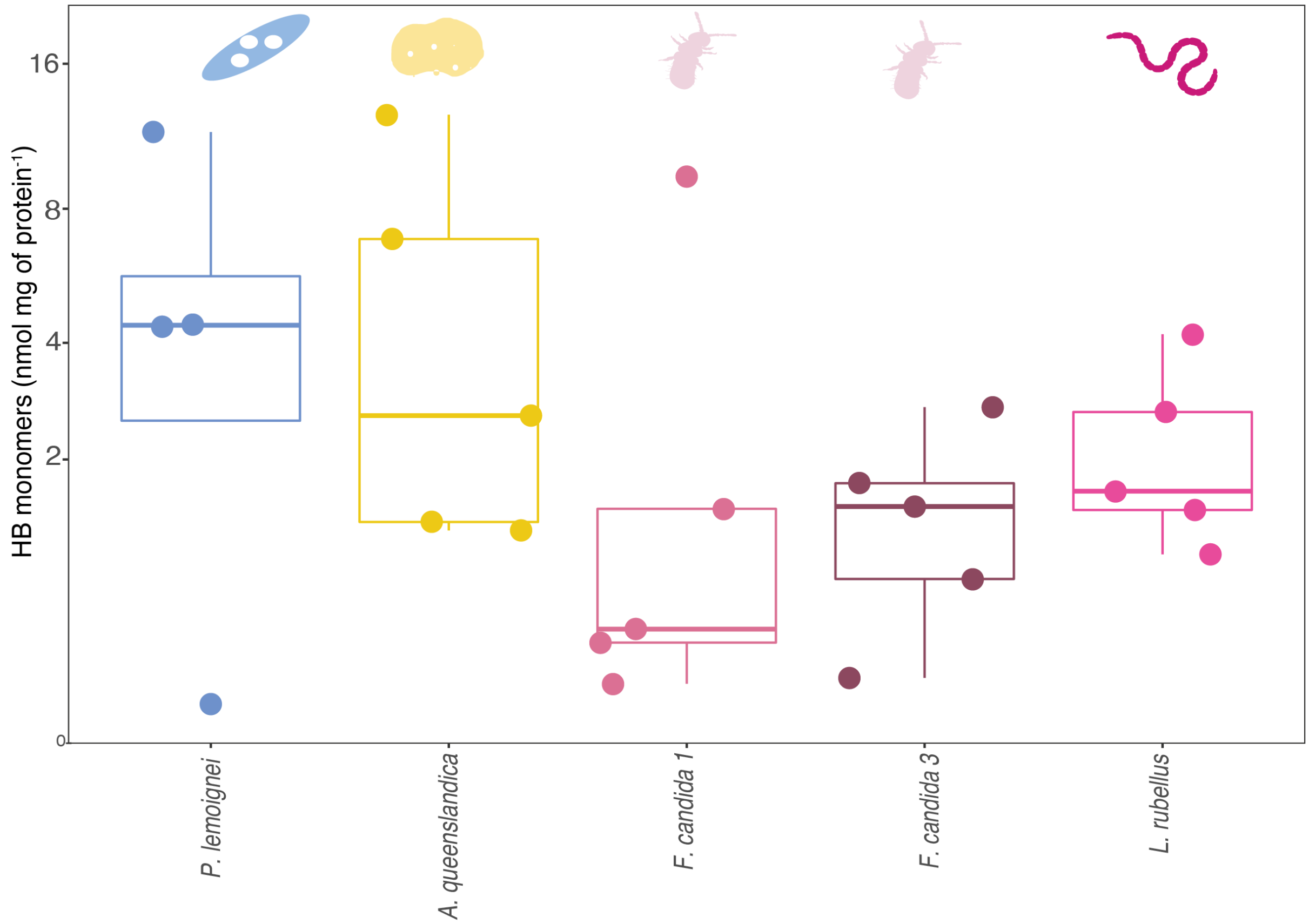

Supplementary Figure 14 | Animal PHADs degrade sPHA into its monomeric units. Boxplots show concentrations of HB monomers, measured by GC-MS, produced in PHAD enzyme assays using PHB/PHV. All tested enzymes generate measurable amounts of HB monomers, including PHADs from the sponge *A. queenslandica* (yellow), the springtail *F. candida* (light pink, two homologs), and the earthworm *L. rubellus* (pink). No HB monomers were detected using a nonsense vector (negative control) or no-enzyme control (see Supplementary Figure 12 & 13). Hydroxyvalerate (HV) monomers were not detected because the PHB/PHV substrate contained only 3% HV, which is below the detection limit of the assay.

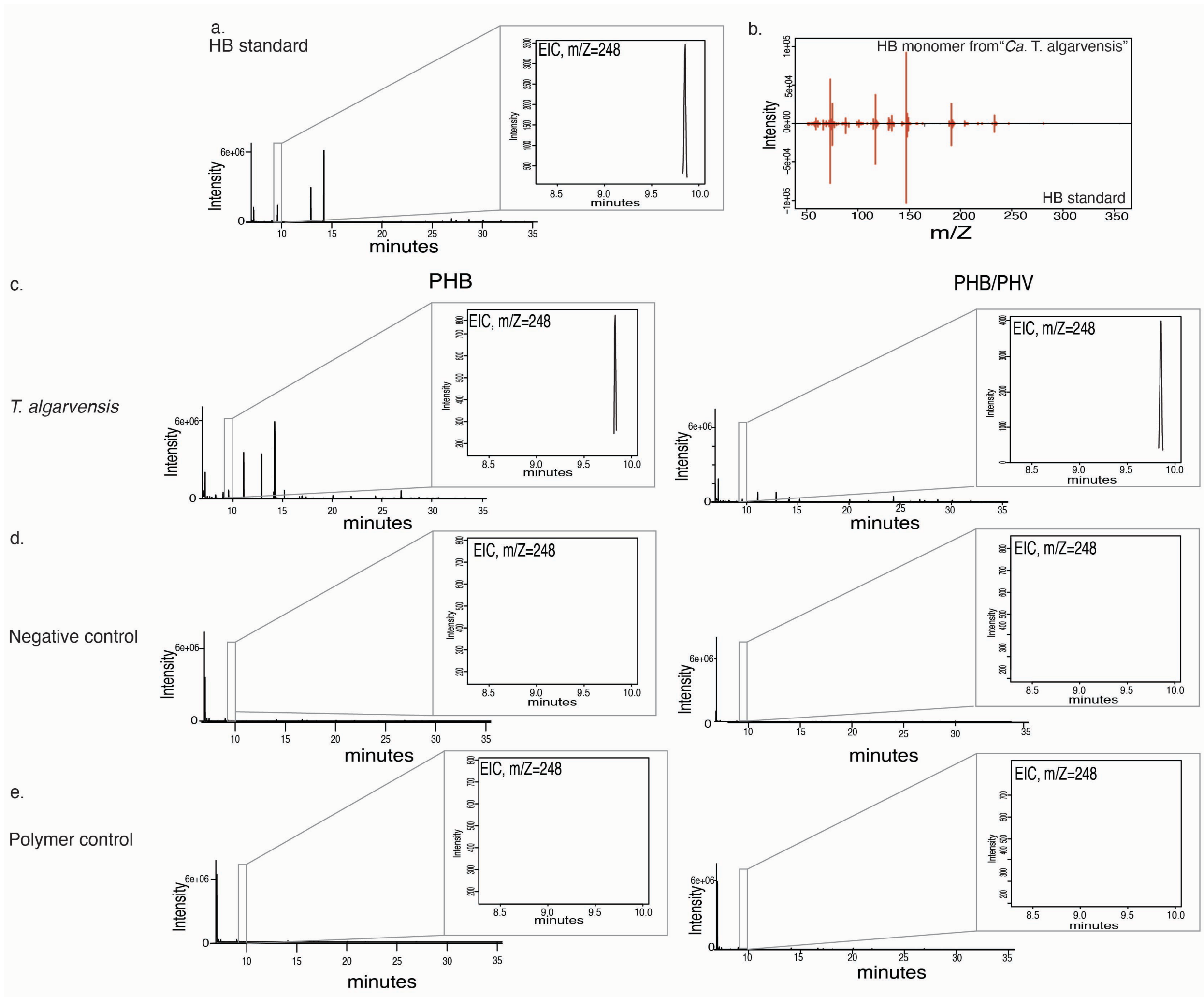

Supplementary Figure 22 | PHAD from *T. algarvensis* degrades PHB and PHB/PHV into HB monomers. a,c,d,e, GC-MS chromatograms of the HB standard, PHAD assays using PHB and PHB/PHV, the no-enzyme control and the negative control with a heat-killed PHAD. No HB monomers were detected in the two controls. Insets in (**a,c,d,e**) show extracted ion chromatograms (m/z 248) corresponding to the HB monomer. **b,** Fragmentation spectra show that HB monomers produced in assays with the *T. algarvensis* PHAD match the retention time (9.56 min) and mass spectrum of the HB standard.

References

1. Wippler, J., M. Kleiner, C. Lott, A. Gruhl, P.E. Abraham, R.J. Giannone, J.C. Young, R.L. Hettich and N. Dubilier, *Transcriptomic and proteomic insights into innate immunity and adaptations to a symbiotic lifestyle in the gutless marine worm* Olavius algarvensis. BMC Genomics, 2016. **17**(1): p. 1-19.
2. Knoll, M., T.M. Hamm, F. Wagner, V. Martinez and J. Pleiss, *The PHA depolymerase engineering database: a systematic analysis tool for the diverse family of polyhydroxyalkanoate (PHA) depolymerases.* BMC Bioinformatics, 2009. **10**: p. 1-8.
3. Saegusa, H., M. Shiraki, C. Kanai and T. Saito, *Cloning of an intracellular poly[d (−)-3-hydroxybutyrate] depolymerase gene from* Ralstonia eutropha *H16 and characterization of the gene product.* Journal of Bacteriology, 2001. **183**(1): p. 94-100.
4. Giere, O. and C. Erséus, *Taxonomy and new bacterial symbioses of gutless marine Tubificidae (Annelida, Oligochaeta) from the Island of Elba (Italy).* Organisms Diversity & Evolution, 2002. **2**(4): p. 289-297.
5. Koller, M. and A. Rodríguez-Contreras, *Techniques for tracing PHA-producing organisms and for qualitative and quantitative analysis of intra- and extracellular PHA.* Engineering in Life Sciences, 2015. **15**(6): p. 558-581.
6. Linton, E., A. Rahman, S. Viamajala, R.C. Sims and C.D. Miller, *Polyhydroxyalkanoate quantification in organic wastes and pure cultures using a single-step extraction and 1H NMR analysis.* Water Science and Technology, 2012. **66**(5): p. 1000-1006.
7. Kleiner, M., L. Polerecky, C. Lott, C. Bergin, S. Haeusler, M. Liebeke, C. Wentrup, N. Musat, M.M.M. Kuypers and N. Dubilier, *Mechanism of high energy efficiency of carbon fixation by sulfur-oxidizing symbionts revealed by single-cell analyses and metabolic modeling.* BioRxiv, 2023: p. 2023.11.25.568684.
8. Reusch, R.N., A.W. Sparrow and J. Gardiner, *Transport of poly-β-hydroxybutyrate in human plasma.* Biochimica et Biophysica Acta (BBA)-Lipids and Lipid Metabolism, 1992. **1123**(1): p. 33-40.
9. Jendrossek, D. and R. Handrick, *Microbial degradation of polyhydroxyalkanoates.* Annual Review of Microbiology, 2002. **56**: p. 403.
10. Dedkova, E.N. and L.A. Blatter, *Role of β-hydroxybutyrate, its polymer poly-β-hydroxybutyrate and inorganic polyphosphate in mammalian health and disease.* Frontiers in Physiology, 2014. **5**: p. 260.
11. Cullingford, E.T., T.C. Dolphin, K.K. Bhakoo, S. Peuchen, L. Canevary and B.J. Clark, *Molecular cloning of rat mitochondrial 3-hydroxy-3-methylglutaryl-CoA lyase and detection of the corresponding mRNA and of those encoding the remaining enzymes comprising the ketogenic 3-hydroxy-3-methylglutaryl-CoA cycle in central nervous system of suckling rat.* Biochemical Journal, 1998. **329**(2): p. 373-381.
12. Hegardt, F.G., *Mitochondrial 3-hydroxy-3-methylglutaryl-CoA synthase: a control enzyme in ketogenesis.* Biochemical Journal, 1999. **338**(3): p. 569-582.
13. Plesofsky-Vig, N., J. Vig and R. Brambl, *Phylogeny of the α-crystallin-related heat-shock proteins.* Journal of Molecular Evolution, 1992. **35**: p. 537-545.
14. Park, J. and J. Saier, MH, *Phylogenetic characterization of the MIP family of transmembrane channel proteins.* The Journal of Membrane Biology, 1996. **153**(3): p. 171-180.

15. Michellod, D., T. Bien, D. Birgel, M. Jensen, M. Kleiner, S. Fearn, C. Zeidler, H.R. Gruber-Vodicka, N. Dubilier and M. Liebeke, De novo *phytosterol synthesis in animals.* Science, 2023. **380**(6644): p. 520-526.
16. Hotelier, T., L. Renault, X. Cousin, V. Negre, P. Marchot and A. Chatonnet, *ESTHER, the database of the α/β-hydrolase fold superfamily of proteins.* Nucleic Acids Research, 2004. **32**(suppl_1): p. D145-D147.

Response to the Reviewers

Manuscript: "Animal degradation of microbial storage polyhydroxyalkanoates"

Authors: Caroline Zeidler[1], Harald R. Gruber-Vodicka[1,2], Dolma Michellod[1], Grace D'Angelo[1], Stefan Becker[3], Henry Berndt[2], Juliane Wippler[1], Marlene Violette[4], Manuel Kleiner[4], Alba Porta-Fidalgo[1], Rebekka S. Janke[1], Kristina Weinert[1], Manuel Liebeke[1,5], Nicole Dubilier[1*], Maggie Sogin[1,6*]

[1]Max Planck Institute for Marine Microbiology, Celsiusstraße 1, 28359 Bremen, Germany

[2] Zoological Institute, University of Kiel, 24118 Kiel, Germany

[3] MARUM Center for Marine Environmental Sciences, University of Bremen, 28359 Bremen, Germany

[4] North Carolina State University, Department of Plant and Microbial Biology, 27607 Raleigh, North Carolina, USA

[5] Institute of Human Nutrition and Food Science, Department of Metabolomics, University of Kiel, 24118 Kiel, Germany

[6] University of California at Merced, Merced, CA 95343, USA

*corresponding authors: **esogin@gmail.com, ndubilier@mpi-bremen.de**

Reviewer #1 (Remarks to the Author):

The authors addressed all of my previous concerns and took my comments to heart. I belive the enzymatic assay added to the results and now shows more convincingly that the enzymes are active against PHAs. I have no further comments to the authors. Thank you for a very interesting piece of work.

We thank the reviewer for their positive comments and the time and effort they put into reviewing our paper as it helped us improve the quality of our manuscript.

Reviewer #2 (Remarks to the Author):

The authors have largely revised the previously submitted manuscript and convincingly have addressed most of the concerns raised by the reviewer(s). The authors show that eukaryotes (here gutless worms) have (active) PHA depolymerases that contribute to the digestion and utilization of bacterial symbionts including the PHA moiety accumulated by the symbionts. This contribution substantially broadens our knowledge on PHA depolymerases and highlight that PHA degradation is not limited to prokaryotes and single cell microorganisms. I therefore support publication of this important contribution.

We thank the reviewer for the careful evaluation and for the valuable feedback provided during the review process. The insightful comments and suggestions were extremely helpful in improving the manuscript.

One minor suggestion:
Main text, lines 111-117 and Suppl. Text, lines 23-46: please provide also similarity values

(not only identity values) of the aligned proteins. Perhaps a suppl. Fig. showing the two aligned (domain) amino acid sequences would be also good.

We agree and added similarity values (main text lines 112 -114; Supplementary Text line 28). We further added a supplementary table (now Supplementary Table 1) that shows the alignment statistics of the 27 experimentally validated PHADs from the PHAD Engineering Database (DED)[1] with the *Olavius algarvensis* PHAD and those of its symbionts.

Reviewer #3 (Remarks to the Author):

I was reviewer # 3 last time and had criticized the lack of functional proof with respect to the predicted enzyme functions.
After reading through the manuscript and the response letter I can acknowledge that the authors have taken most of my comments into consideration and addressed them.
Using MS technologies but also halo formation and after overexpressing selected genes and enzymes they now have preliminary data that strongly suggest that the predicted enzymes are functional and involved in PHA transformation.

We thank the reviewer for the time and effort they spent reviewing the paper and providing thoughtful and constructive feedback.

While this is positive, strong and solid kinetic data on enzyme turnover would be favorable.
I would urge the authors to clearly state that the substrates converted might not be the natural substrates of the enzymes detected. It is well known that alpha-beta hydrolases are highly promiscuous enzymes and that they often randomly turn over – albeit at very low rates - a very wide range of different substrates. The literature indicates that some of these enzymes may have the potential to degrade over 80 different substrates. So it remains questionable if the PHAs are the native and given the small halo forming activities.
Nevertheless this is a very interesting and well written manuscript that makes an important contribution to the field.

We agree that kinetic data and a more detailed biochemical characterization would be valuable and represent an important direction for future work. However, such analyses are not required for the principal conclusion of this study, namely that animals possess enzymes capable of depolymerizing microbial storage PHAs into hydroxyalkanoate monomers. Using our newly developed GC-MS-based assays, we demonstrated this activity for PHADs from multiple animal lineages from both terrestrial and aquatic environments. Importantly, these assays showed that animal PHADs hydrolyze microbial sPHAs into hydroxyalkanoate monomers, the defining reaction of microbial PHADs. While other α/β-hydrolases, including lipases and cutinases, can hydrolyze a variety of polyester substrates, they have not been shown to catalyze this characteristic PHAD reaction[2,3].

We acknowledge that other α/β-hydrolases can exhibit substrate promiscuity and therefore cannot formally exclude the possibility that animal PHADs act on additional substrates. However, several lines of evidence support microbial sPHAs as biologically relevant substrates. First, animal PHADs cluster phylogenetically with experimentally validated bacterial, fungal, and protist PHADs. In addition, our phylogenetic analyses showed that PHADs form a distinct clade separate from cutinases and lipases, and that the catalytic triad and substrate-binding motifs of PHADs do not align with those of these related α/β-hydrolase families. Second, PHAD homologs occur across animal lineages whose feeding ecologies

provide regular access to microbial sPHAs through symbiosis, detritivory, bacterivory, or filter-feeding. Third, in the gutless annelid *Olavius algarvensis*, PHAD is expressed in host cells responsible for digesting sPHA-producing symbionts. Together, these findings support the interpretation that microbial sPHAs are physiologically relevant substrates of animal PHADs, while recognizing that animal PHADs may have additional substrates and that future kinetic analyses will help define substrate specificity in greater detail.

References

1. Knoll, M., T.M. Hamm, F. Wagner, V. Martinez and J. Pleiss, *The PHA depolymerase engineering database: a systematic analysis tool for the diverse family of polyhydroxyalkanoate (PHA) depolymerases.* BMC Bioinformatics, 2009. **10**: p. 1-8.
2. Masaki, K., N.R. Kamini, H. Ikeda and H. Iefuji, *Cutinase-like enzyme from the yeast* Cryptococcus *sp. strain S-2 hydrolyzes polylactic acid and other biodegradable plastics.* Applied Environmental Microbiology, 2005. **71**(11): p. 7548-50.
3. Tokiwa, Y. and T. Suzuki, *Hydrolysis of polyesters by lipases.* Nature, 1977. **270**(5632): p. 76-78.
